# Why Do Cuckolded Males Provide Paternal Care?

**DOI:** 10.1371/journal.pbio.1001520

**Published:** 2013-03-26

**Authors:** Ashleigh S. Griffin, Suzanne H. Alonzo, Charlie K. Cornwallis

**Affiliations:** 1 Edward Grey Institute, Department of Zoology, University of Oxford, Oxford, United Kingdom; 2 Department of Ecology and Evolutionary Biology, Yale University, New Haven, Connecticut, United States of America; 3 Department of Biology, Lund University, Lund, Sweden; University of Georgia, United States of America

## Abstract

In most species, males do not abandon offspring or reduce paternal care when they are cuckolded by other males. This apparent lack of adjustment of paternal investment with the likelihood of paternity presents a potential challenge to our understanding of what drives selection for paternal care. In a comparative analysis across birds, fish, mammals, and insects we identify key factors that explain why cuckolded males in many species do not reduce paternal care. Specifically, we show that cuckolded males only reduce paternal investment if both the costs of caring are relatively high and there is a high risk of cuckoldry. Under these circumstances, selection is expected to favour males that reduce paternal effort in response to cuckoldry. In many species, however, these conditions are not satisfied and tolerant males have outcompeted males that abandon young.

## Abstract 2

A comparative analysis across insects, birds, fish, and mammals reveals why it sometimes pays for males to care for the offspring of other males.

## Author Summary

In most species where it has been studied, males do not abandon or reduce paternal care when they are cuckolded by other males. These observations have presented a long-standing challenge to our understanding of what drives selection for paternal care. Our analysis of cuckolded fathers from 50 species of birds, fish, mammals, and insects, however, shows that sometimes it pays for males to stick around. In the case of humans and burying beetles, this is because females are relatively monogamous—by deserting, it is most likely the case that fathers will be deserting their own young. In species such as the chacma baboon, males face a significant risk of cuckoldry, and face potentially high penalties in terms of future breeding success by wasting precious resources on the young of other males. Unlike in humans, promiscuous females in these species will almost certainly lose the support of her mate in the effort to raise her young to adulthood.

## Introduction

Parental care is demanding: the effort it takes a typical garden bird to raise a clutch of chicks to adulthood is equivalent, in human terms, to cycling the Tour de France [1]. Intuition suggests that a male should only embark on this feat if he is the father of the chicks in his nest—if he has been cuckolded, he should avoid wasting resources on enhancing the reproductive success of his rivals and reduce paternal effort. Forty years of empirical research, however, have failed to provide consistent support for this prediction [2]. While there are exceptions, in the majority of species, it is reported that males do not significantly decrease care when cuckolded [2]–[5], challenging our understanding of how natural selection favours males that provide parental care.

If information about paternity is unavailable or unreliable, selection may favour males that continue to provide care to avoid potential costs of abandoning their own offspring [6],[7]. Consequently, attempts to test explanations for the lack of paternal care adjustment have focussed on the ability of males to accurately assess paternity, but this has yielded abundant unexplained variation between species [2]. Another explanation is that variation in adjustment reflects differences in the strength of selection on males to reduce paternal care in response to loss of paternity. Firstly, theory predicts that the behaviour of males should optimise lifetime reproductive success rather than just paternity [8],[9]. This is formalised in Hamilton's Rule, *rb*−*c*>0, where *r* is the relatedness between the caring male and offspring in this context, *b* is the fitness benefit of care to offspring, and *c* is the cost of care to future reproductive success of the male [10]. Cuckolded males are, therefore, predicted to be relatively tolerant of cuckoldry if paternal care does not reduce future reproductive success (low *c*) [8],[11],[12], and/or paternal care has little effect on offspring fitness (low *b*) [10],[13]. When *b* is low, variation in *r* has relatively weak effect on variation in selection (if we substitute *b* = 0 into *rb−c*>0, *rb* is always 0; if we substitute *b* = 1 into *rb−c*>0, *rb* varies from −1 to 1 depending on the value of *r*). Secondly, when there is little variation in the risk of cuckoldry between breeding attempts, or cuckoldry is rare, there will be weak selection for adjustment [11]. This is because rare or low variation in cuckoldry within males reduces the opportunity for selection to favour individuals that adjust paternal care [14]. Despite well-developed predictions about when cuckolded males should reduce care, our understanding of this problem remains limited because empirical studies have focused on reporting the presence or absence of adjustment without further formal analyses of causation.

## Results

We conducted a series of comparative meta-analyses to characterise the evolution of paternal care adjustment across species. We first quantified the strength of adjustment by calculating a standardised effect size (Pearson's correlation coefficient: r) from the statistics reported in 62 studies that measured the relationship between paternity and paternal care across 48 species of fish, insects, birds, and mammals. This effect size, r_Adjust_, is the correlation coefficient between paternal investment and paternity: positive values of r_Adjust_ indicate that reductions in paternal care are associated with loss of paternity; a value of zero indicates that investment in paternal care is independent of paternity; and negative values indicate paternal effort decreases with higher paternity.

**Figure 1:**
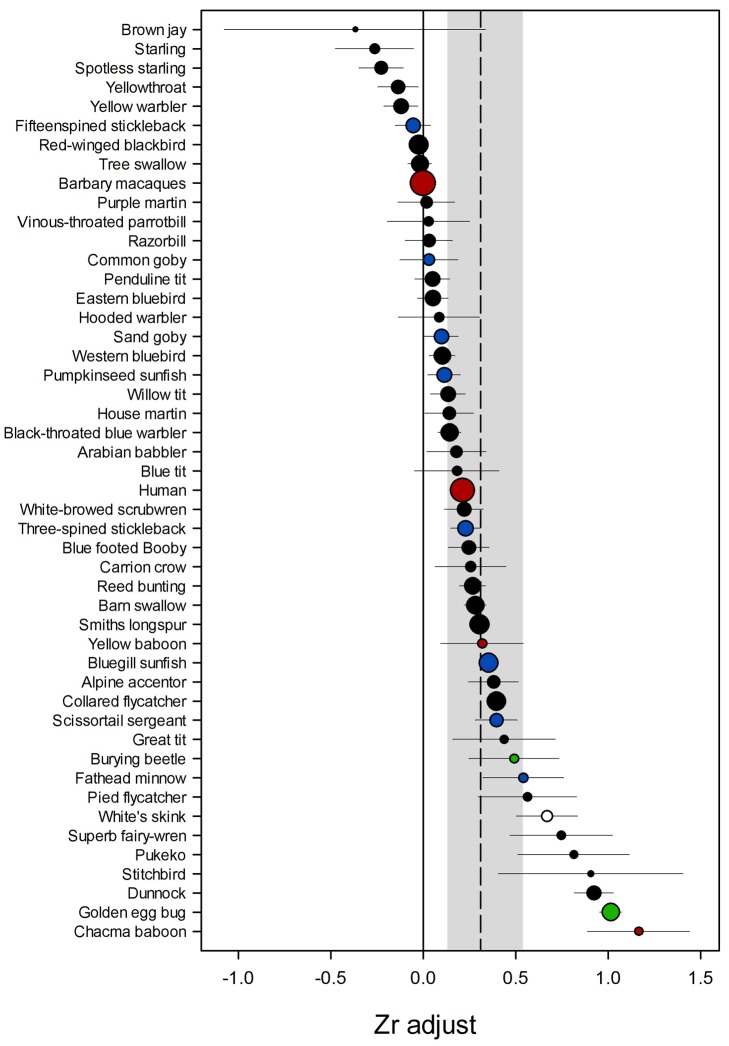
Meta-analysis of paternity on paternal care across species. Points indicate mean adjusted effect sizes (Zr_Adjust_) for each species with SE bars. The dashed line represents mean adjusted effect size of paternity on paternal care with 95% CI (grey box). Size of circle indicates the number of studies contributing to weighted mean (See Table S1); colour of circle indicates taxonomic group: black, birds; red, mammals; green, insects; blue, fish; white, reptile.

In contrast to the prevailing consensus across empirical studies, we found that males show a significant reduction in paternal care in response to female promiscuity across species. We found that r_Adjust_ was positive in 81% of species and was significantly greater than zero overall (r_Adjust_ mean effect = 0.35, 95% credible interval (CI) = 0.10–0.68, *p* = 0.02; Figure 1; Table 1), despite the fact that only 44% of studies in our dataset report a significant effect of paternity on paternal care (Table S1). The difference between our meta-analysis of the strength of adjustment and “vote-counting” of significant results, indicates that failure to detect weak effects (type II error) and large variation between studies (Table 1) may have contributed to an underestimate of the extent to which males respond to cuckoldry. We also found that experimental manipulation of a male's certainty of paternity did not cause greater adjustment in paternal care compared to observational studies (Table 1). Furthermore, r_Adjust_ was significantly greater than 0 across experimental and observational studies and across studies that had measured different cues and used different methodology (Table S6). Together these results suggest that the ability of females to conceal promiscuous mating from males has been overestimated and the reported failure of studies to detect male adjustment cannot solely be explained by inaccuracy of cues.

**Table 1:** Summary of five meta-analyses.

					Variation Explained by Random Effects (%)				
Response Variable	Explanatory Variable	*n* a	Posterior Mean r-Values (CI^b^)	*p* c	Class	Family	Species	Study	Unexplained
(A) r_Adjust_	Mean effect size	48/192	0.35 (0.10–0.68)	**0.02**	34.78	17.99	9.17	36.15	1.91
(B) r_Adjust_	Probability of care	—	0.45 (0.13–0.81)	**0.001**	35.75	17.13	11.71	33.68	2.23
	Amount of care	—	0.31 (0.001–0.67)	**0.04**	—	—	—	—	—
	Female access: yes	—	0.42 (0.10–0.75)	**0.01**	—	—	—	—	—
	Female access: no	—	0.31 (0.004–0.65)	**0.04**	—	—	—	—	—
	Competitor: yes	—	0.22 (−0.11 to 0.57)	0.15	—	—	—	—	—
	Competitor: no	—	0.31 (0.02–0.68)	**0.04**	—	—	—	—	—
	Genetic data: yes	—	0.29 (0.03–0.58)	**0.02**	—	—	—	—	—
	Genetic data: no	—	0.31 (0.01–0.66)	**0.04**	—	—	—	—	—
	Experimental	—	0.32 (0.01–0.69)	**0.04**	—	—	—	—	—
	Observation	—	0.32 (0.01–0.63)	**0.04**	—	—	—	—	—
	Within male tests	—	0.74 (0.36–1.17)	**0.003**	—	—	—	—	—
	Across male tests	—	0.31 (0.003–0.68)	**0.05**	—	—	—	—	—
(C) r_Adjust_	Zr_Benefit_ (*b*)	—	−0.03 (−0.12 to 0.07)	0.57	17.82	20.55	14.23	45.24	2.73
	Zr_Cost_ (*c*)	—	0.02 (−0.11 to 0.15)	0.77	—	—	—	—	—
	Multiple paternity (*r*)	—	0.02 (−0.07 to 0.10)	0.62	—	—	—	—	—
	Zr_Cost_: multiple paternity	—	0.11 (0.01–0.22)	**0.02**	—	—	—	—	—
(D) r_Benefit_	Mean effect size	34/109	0.47 (−0.27 to 0.87)	0.15	65.02	1.62	15.58	10.66	7.12
(E) r_Cost_	Mean effect size	24/45	0.26 (0.02–0.50)	**0.04**	42.44	15.36	9.33	7.9	24.97

(A) Overall strength of paternal care adjustment (r_Adjust_), (B) effect of methodology on r_Adjust_, (C) effect of multiple paternity (*r*), benefit of paternal care (*b:* r_Benefit_) and costs of paternal care (*c*: r_Cost_) on r_Adjust_, (D) overall benefit of paternal care on offspring fitness (r_Benefit_), and (E) the costs of care on male future reproductive success (r_Cost_).

a Species/effect sizes.

b CI = 95% CI.

c *p*-Value calculated in MCMCglmm = number of simulations greater than 0 corrected for number of MCMC samples. Bold type represents *p-*values<0.05.

In addition to the significant overall effect of paternity on paternal care, our analysis revealed high variation in the strength of adjustment among species (Figure 1; Table 1), even after accounting for the possible confounding effects of phylogenetic history and differences in methodology (Tables 1 and 6). We tested predictions a priori that males of some species have stronger paternal care adjustment than others because of differences in the costs and benefits of paternal care and the risk of cuckoldry. We estimated the cost of care (*c*) by calculating the effect size (Pearson's correlation coefficient: r) of paternal investment in a current breeding attempt on the probability of success in future breeding attempts (r_Cost_) from published test statistics (Table S2). A positive value of r_Cost_ indicates that the level of investment in care for offspring in a current breeding attempt results in a correlated reduction in future breeding success. We estimated the benefits of paternal care (*b*) by measuring both the effect size (Pearson's correlation coefficient: r) of male care on offspring fitness (r_Benefit_) from published test statistics and by obtaining data on the proportion of parental feeding visits performed by males from the literature (Table S3). A positive value for r_Benefit_ indicates that more paternal care results in higher offspring survival. Finally, we estimated variation in female promiscuity (*r*) from the proportion of broods containing offspring fathered by more than one male (Table S1).

**Figure 2:**
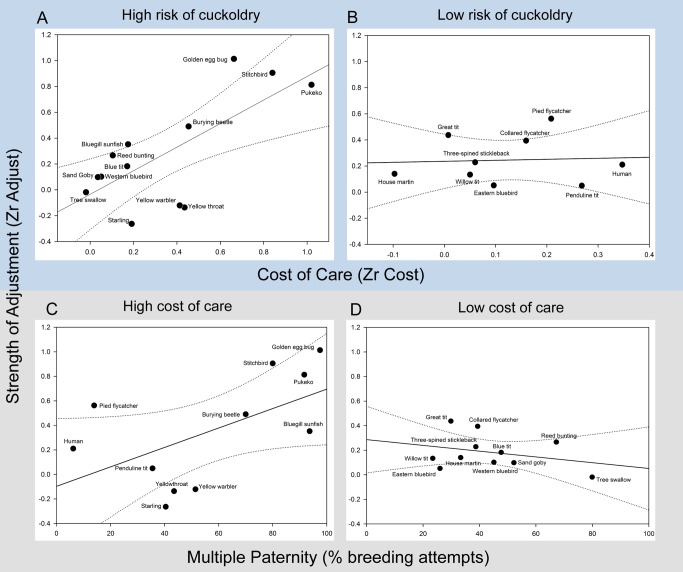
The strength of adjustment of paternal care is determined by factors affecting male reproductive success. The adjustment of paternal care is determined by an interaction between the risk of cuckoldry and the costs of care to future reproductive success. Zr_Adjust_ was positively related to Zr_Cost_ when multiple paternity was high ((B) multiple paternity is above the median), but not when multiple paternity was low ((A) multiple paternity less than or equal to the median). Similarly when opportunity costs to males of caring were high ((D) Zr_Cost_ greater than the median) Zr_Adjust_ increased with rates of multiple paternity, which was not the case when costs to males were low ((C) Zr_Cost_ less than the median). Regression lines are presented with 95% CIs. Zr_Cost_ and multiple paternity were analysed as continuous variables and have only been dichotomized for graphical purposes.

**Figure 3:**
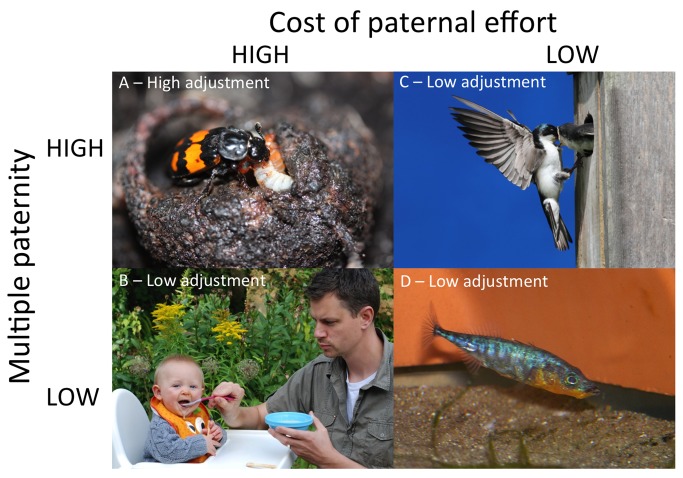
Four case studies of paternal care adjustment in response to female promiscuity. (A) Males of the burying beetle genus *Nicrophorus*, are unique among insects in the level of paternal care provided, and this care is costly: males reduce future reproductive success by caring for offspring in a current brood. Males also have a relatively high chance of being cuckolded. These two factors drive selection for male burying beetles to spend less time with their brood if they have been cuckolded. (B) Like burying beetles, male humans, *Homo sapiens*, reduce their chance of breeding successfully in future by providing paternal care, but relatively low risk of cuckoldry reduces selection for adjustment. (C) Paternal care can be extremely demanding in nesting birds, but in the case of the tree swallow, this energetic cost has relatively weak effect on the chance that males will breed successfully in future. Consequently, adjustment is relatively weak, despite the fact that females are relatively promiscuous. (D) This male three-spined stickleback, *Gasterosteus aculeatus*, cares for his eggs by defending them from predators and keeping them well-oxygenated by fanning, but he may also start to eat them if he is hungry. Compared to burying beetles, however, paternity has less influence on a male's care decisions because female promiscuity and the cost of caring are both relatively low in this species. Photos courtesy of P. Smiseth (burying beetle), E. Cornwallis (humans), S. Byland (tree swallow), and M. Head (three-spined stickleback).

We found that cuckolded males are significantly more likely to provide care when both the cost to future reproductive success is relatively low and cuckoldry is relatively rare (interaction between multiple paternity and r_Cost_ explained 13% of variation in r_Adjust_ across species (reduction in residual variation when interaction was included in model): parameter estimate (β) = 0.11, CI = 0.01–0.22, *p* = 0.02; Figures 2 and 3, Table 1). The importance of this interaction is evident from the fact that a failure to reduce care in response to cuckoldry may be favoured even in species with a high risk of cuckoldry if there is relatively little cost to future reproductive success (Figure 2). In this case, the advantage of saving resources from withholding care are less likely to outweigh the costs of abandoning a brood where the male may have achieved some paternity success. Conversely, males also continue to care where the costs to future reproductive success are relatively high if female promiscuity is rarely or never encountered (Figure 2). This is because selection will not have had the opportunity to equip males with the ability to detect loss of paternity accurately and/or respond appropriately. For most species, however, some degree of adjustment has been favoured (Figure 1) and our results suggest that this is because caring for current offspring is generally costly to future reproductive success (r_Cost_ is significantly above zero across species – r_Cost_ mean effect size and CI = 0.26 [0.02–0.50], *p* = 0.04; Table 1) and the chance of cuckoldry is sufficient to drive the evolution of a response.

In contrast, we found no evidence that variation in the benefit of paternal care on offspring fitness explains differences between species in adjustment. Males were no more likely to adjust in species with high r_Benefit_ relative to species with low r_Benefit_ (mean effect and CI = −0.03 [−0.12 to 0.07, *p* = 0.57; Table 1) and males that provided a greater proportion of total parental care were not more sensitive to paternity (mean effect and CI = −0.003 [−0.15 to 0.16], *p* = 0.95; Table S7). The effect of male care on offspring survival is relatively high across species (mean r_Benefit_ = 0.47 (−0.27 to 0.87; Table 1) and we speculate that within biparental or male-only care systems the effect of male care may be difficult to detect and measure (Table S4; Text S1). For example, the effect of male desertion is often not documented if offspring always die without male care.

The data we collected on r_Adjust_ comes from studies that measured the relationship between paternal care and paternity certainty both within and across males. It is therefore possible that our results are not only due to facultative adjustment of care, but also intrinsic differences between males. For example, a positive relationship between paternity and paternal care (positive r_Adjust_) may arise because poor quality males care less and are more often cuckolded [15], or because individual males adjust care in response to perceived paternity. To examine this possibility, we tested if our results differed according to whether r_Adjust_ was measured across males or within males across breeding attempts. Values of r_Adjust_ were significantly higher when changes within males were examined (Table 1), but the effect of r_Cost_ and rates of cuckoldry on r_Adjust_ were consistent across studies using between and within subject designs (Table S7(i)). Furthermore, estimates of r_Adjust_ were highly positively correlated across species where the relationship between paternity and paternal care had been measured both across and within males (Spearman's Rank Correlation: R_s_ = 1.00, *n* = 4, *p*<0.001, Pearson's correlation coefficient: R = 0.90). Together these results suggest that examining changes within males may facilitate detection of paternal care adjustment and verifies that males facultatively reduce care in response to lowered paternity confidence, especially in species where high rates of cuckoldry are combined with high costs of caring to future reproductive success.

## Discussion

In this study, we address problems arising from the lack of empirical tests of theoretical predictions about the evolution of paternal care adjustment. In particular, measuring variation in paternity and the costs and benefits of paternal care within species is a major undertaking and it remains a challenge to think of ways how these factors can be experimentally manipulated to test existing theoretical predictions. By adopting a comparative approach, we have been able to exploit variation between species and our results suggest several important considerations for future studies of paternal care adjustment. Firstly, our study provides some guidance about the characteristics of species best suited to studies of paternal care adjustment. Specifically, the expectation of adjustment should be lowered in species with either low costs of care or low promiscuity. Secondly, we suggest that studies more closely address theoretical predictions by linking measures of adjustment with measures of costs of care (none of the measures in our adjustment dataset were directly linked with the measures of costs [Tables S1 and S2]). Thirdly, the interaction between promiscuity and cost reported here could be tested empirically within a single species by characterising the relationship between the strength of adjustment and the residual reproductive value of males. This could be achieved by exploiting individual variation in factors such as male age, timing of breeding or quality, which are expected to correlate with residual reproductive value.

When we see males caring for the offspring of another male, it is possible to assume that they are doing so simply because of a failure to accurately determine paternity success. Although our analysis shows that males are not as constrained by lack of reliable cues to paternity as often thought, it remains the case that females seem to get away with promiscuous mating in some species, with cuckolded males continuing to provide care. Are cuckolded males that maintain care blissfully ignorant or selfless dupes? Our analyses suggest they are neither; instead, whether or not cuckolded males reduce paternal care is readily explained by the cost of paternal care and the risks of cuckoldry. More generally, cuckolded males provide a good example of how selection favouring tolerance can lead to the appearance of losing in an evolutionary arms race with cheats [16].

The finding that males show some degree of response to cuckoldry in the majority of species studied has implications for the evolution of paternal care more generally: we estimate that the extent to which females reduce paternal investment by engaging in promiscuous copulations is 12% on average within-species. By changing relatedness between nest mates, it has been shown that female promiscuity can drive transitions to and from cooperative breeding in birds [17]. By lowering relatedness between males and offspring, female promiscuity also has the potential to drive selection for reduced levels of paternal investment [18]–[20] and may ultimately cause the breakdown of biparental breeding systems [21],[22].

## Materials and Methods

### Data Collection

#### Adjustment of paternal care

We quantified the adjustment of paternal care by calculating the effect size of the relationship between paternal care and paternity (r_Adjust_) for all species with available data using the effect size calculator in metawin on test statistics reported in papers. We searched the Web of Science for published papers that included the keywords “care” and “paternity” and then conducted forward and backward searches through cited references of these papers (search performed on papers published up to and including 1 March 2011). We also contacted researchers within the field to check for availability of unpublished data. In total, we obtained 192 effect sizes from 62 papers representing 48 species, from 29 families and five different classes. For each effect size we recorded: (a) whether the amount or the probability (i.e., desertion) of paternal care was measured (two-level factor), (b) whether the data were observational or experimental (two-level factor), (c) whether molecular genetic techniques had to been used to assess parentage (yes/no: two-level factor), and (d) the potential cues available to males the study had measured, which included access to females during the fertile period (yes/no: two-level factor) and the presence of potential competitors (yes/no: two-level factor). For the species (*n* = 48) with data on the adjustment of paternal care we then collected information on the benefits of male care for offspring, the costs of care for male future reproductive success, and the risk of that females are promiscuous.

#### Measuring the benefit of male care for offspring

To assess the benefits of paternal care for offspring we calculated effect sizes from studies that measured the relationship between male care and offspring fitness (r_Benefit_; Figure S1). We located data by checking the references of the studies on the adjustment of paternal care, contacting authors working on target species, and searching the Web of Science. In the Web of Science we searched using the latin name of the species as a keyword and if the number of results were over 200 we also added the term “care OR paternal”. For each effect size we also recorded (a) if the probability or amount of paternal care had been measured (two-level factor), (b) whether the study was observational or experimental (two-level factor), and (c) how offspring fitness was measured (three-level factor: condition, survival, or recruitment).

#### Measuring the costs of care to male future reproductive success

To quantify the cost of paternal care to male future reproductive success (opportunity costs) we calculated the effect size of the relationship between male care in a current breeding attempt with measures of success in the future (r_Cost_; Figure S2). We located studies using exactly the same methods as those used to find studies on r_Benefit_. Once again we recorded (a) if the probability or amount of paternal care had been measured (two-level factor), (c) whether the study was observational or experimental (two-level factor), and (c) how male future success was measured (three-level factor: attracting mates, reproductive success once paired [number of offspring] or survival). We also recorded the type of care that males provided in studies measuring r_Benefit_, r_Cost_, and r_Adjust_, but it was not possible to analyze if this influenced effect sizes because it was confounded with taxonomic grouping. For example, only male fish care for offspring by fanning eggs.

#### Measuring the risk of female promiscuity

We quantified the probability that males care for unrelated offspring using data from molecular genetic studies that have measured levels of multiple paternity. Previously we have collected data on birds [17], but for other species we located data using the same methods as those used to find studies on the benefits and costs of paternal care. We defined multiple paternity as the percentage of families in the population that had offspring sired by more than one father (range = 6.1%–97.5%). We also collected data on the percent offspring sired by males other than the caring male (range = 5.3%–71.2%). However, we used the probability of males being cuckolded as our measure of relatedness to offspring because it captures variation in multiple paternity at the level of the population, more data were available and it is highly correlated to the percentage of offspring fathered by other males (Pearson's correlation coefficient = 0.84).

#### General statistical techniques

We analysed variation in r_Benefit_, r_Cost_, and r_Adjust_ with taxonomic random effect meta-analyses performed using Bayesian linear mixed models (BMM) with Markov chain Monte Carlo estimation in MCMCglmm, R version 2.13.1 [23],[24]. Effect sizes were transformed to Fisher's *Z* (*Zr*) before analysis
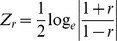
and weighted by the inverse variance to account for variation in sample size between studies. The variance associated with effect sizes was calculated as the reciprocal of the sum of the conditional variance,
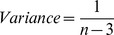
where *n* is the sample size of the study [25]. Prior to analyses covariate fixed effects were Z-transformed (mean = 0, standard deviation = 1) and two-level fixed effects were converted to binary coding −1, 1 so that the magnitude of parameter estimates could be compared and main effects could be interpreted in the presence of higher order interactions [26],[27].

The data in all analyses were from a taxonomically diverse range of species and for some species there were multiple studies and multiple effect sizes per study. We took into account the non-independence of data arising from multiple studies on the same species, and from the phylogenetic relationships between species by defining a nested random effects structure whereby study was nested within species, species was nested within family, and family was nested within class [23]. Only family and class were entered into models because in our dataset there was no replication at the level of genus and order.

We ran each analysis for 3,000,000 iterations with a burn-in of 2,500,000 and a thinning interval of 100. This generated 5,000 samples from each chain from which model statistics (deviance information criteria [DIC], posterior mean ± standard deviation [SD], posterior mode and 95% CIs [lower CI–upper CI]) were calculated. Terms were considered statistically significant when 95% CIs did not span 0 and pMCMC values calculated in MCMCglmm (number of simulated cases that are >0 or less than 0 corrected for finite number of MCMC samples) were less than 0.05 [24]. Initially we tried using two different priors. First, we used an inverse gamma prior that is commonly used for random effects (V = 1, nu = 0.002). Second, we ran models with parameter expanded priors (half-Cauchy priors following [28]: *V* = 1, *nu* = 1, alpha.mu = 0, alpha.V = 25∧2) due to some variance components being close to 0. The inverse gamma prior led to better convergence as measured by the Gelman-Rubin statistic (see below) and produced almost identical results to equivalent frequentist models run with ASReml-R version 3 [29]. We therefore used priors with V = 1, nu = 0.002 for all models. Missing values in explanatory variables were imputed using the mean of the missing variable (Z-transformed scale = 0) so that it was possible to compare different models using DIC [30].

We checked the convergence of each analysis using two diagnostic tests in the R package “coda” [31]. First, we ran each analysis three times and used the Gelman-Rubin statistic (potential scale reduction factor [PSR]) to compare within- and between-chain variance [32]. When convergence is met PSR<1.1 and in all our analyses PSR was less than 1.01. Second, we used Geweke's convergence diagnostic, which calculates Z scores from mean parameter estimates ± standard errors (SEs) generated from the first 10% and the last 50% of the chain [33]. If Z scores follow an asymptotically standard normal distribution the samples are considered to be drawn from a stationary distribution.

#### Tests for publication bias

We tested whether there was evidence for publication bias/small-study effects (smaller studies show greater effects than larger studies) in estimates of r_Benefit_, r_Cost_, and r_Adjust_ using trim and fill analysis and funnel plot asymmetry tests.

#### Trim and fill analysis

We conducted trim and fill analyses on r_Benefit_, r_Cost_, and r_Adjust_. It is currently not possible to implement trim and fill methods in the Bayesian mixed models that were used to conduct the meta-analyses. We therefore performed the trim and fill analysis using a random-effects meta-analysis with restricted maximum likelihood estimation (REML) in the R package “metaphor” [34]. It must be noted that this does not allow for the non-independence of multiple effect sizes per study and taxonomy to be taken into account, but still provides a test of the influence of small-sample effects across all data points on the mean effect size. For r_Benefit_ and r_Adjust_ the trim and fill analyses estimated that no points were missing due to small-study effects (Figure S3a and S3b). For r_Cost_ 6 points were estimated to be missing resulting in an estimated effect size of 0.12 (95% confidence limits: 0.03–0.22) rather than 0.19 (95% confidence limits: 0.11–0.26) (Figure S3c). Together these results indicate that at the level of individual studies r_Benefit_ and r_Adjust_ show little evidence of being influenced by small-study effects and that significance of the mean effect size of r_Cost_ is not changed by publication bias.

#### Funnel plot asymmetry

We carried out regression tests of funnel plot asymmetry using Egger's regression method on the full datasets used in the meta-analyses [35]–[37]. Egger's regression tests the relationship between the effect size and its associated SE, weighted by the inverse of the estimated effect size variance. In order to control for the non-independence of effect sizes from the same study and taxonomy we used Bayesian mixed models with the same random effects structure and settings (priors, iterations, burn-ins, thinning, and convergence testing) that were used for the meta-analyses. Effect sizes were entered as the response variable weighted by 1/se^2^ and se was entered as a covariate. For r_Benefit_, r_Cost_, and r_Adjust_ the confidence intervals of the relationship between effect size and SE all spanned 0 and pMCMC>0.80. This further indicates that small-sample effects did not have a strong influence on the estimation of r_Benefit_, r_Cost_, or r_Adjust_.

#### Specific analyses

We conducted five sets of meta-analyses: (1) First, we tested if variation in r_Benefit_ was explained by methodological (fixed effects: amount versus probability, observation versus experiment, measure of offspring fitness) and taxonomic/study effects (random effects) (Tables S4a–S4f); (2) Second, we tested if variation in r_Cost_ was explained by methodological effects (fixed effects: amount versus probability, observation versus experiment, measure of cost), the benefits males provide to offspring (r_Benefit_ and proportion of male care: covariate fixed effects) and taxonomic/study effects (random effects) (Tables S5a–S5h); (3) Third, we tested if r_Adjust_ was explained by methodological effects (fixed effects: amount versus probability, observation versus experiment, use of genetic methods, male access to females, presence of competing males) (Tables S6a–S6h); (4) Fourth, we tested if r_Adjust_ was explained by biological variables (fixed effects: r_Benefit_ [covariate], r_Cost_ [covariate], multiple paternity and multiple paternity [covariates] [23]), and taxonomic/study effects (random effects) after controlling for any methodological differences found in analyses 1–3 (Tables S7a–S7h); (5) Fifth, we tested if r_Adjust_ and the effects found in analyses 1–4 were influenced by phylogenetic history not accounted for by the nested taxonomic random effects structure. To do this we had to restrict the data to only birds (32 species) for which there is a phylogeny. We used a bird supertree [17] that was pruned to include only species for which there were data on r_Adjust_ using the “ape” package in R [38]. We used a Phylogenetic Bayesian mixed model (BPMM) that was exactly the same as in analysis 4, but instead of fitting a nested taxonomic random effects structure we fitted the phylogeny and study as random effects.

For analyses 1–4 we used the following procedure. First, we tested fixed effects individually to estimate the mean effect of each variable. Second, we ran full models to estimate the marginal effects of each variable controlling for the effects of all other variables. Third, we ran full models sequentially adding in all biologically relevant interactions (r_Cost_ * r_Benefit_ to test if adjustment is increased by the multiplicative effects of costs and benefits: r_Cost_ * multiple paternity to test if adjustment is greater when costs are high and there is a high chance that males are cuckolded: r_Benefit_ * multiple paternity to test if adjustment is higher in species where males confer greater benefits to offspring and the risk of cuckoldry is high) and then selected the best model using DIC values. For each set of analyses we present a model summary table that details each model fitted, its DIC value, and the percentage of variation in effect sizes explained by each random term (Tables S4a, S5a, S6a, and S7a). The percentage of variation explained by each random effect, in conjunction with the estimates of variance components, gives an indication of whether different taxonomic levels and different studies share a common effect size (see Horvathova et al. [39] for similar approach to quantifying heterogeneity). We then present tables for each analysis with estimates of all fixed and random effects (Tables S4b–S4f, S5b–S5h, S6b–S6h, S7a–S7h). In the main text we transform effect sizes from *Zr* back to *r* and present posterior means, credible intervals and pMCMC values.

## Supporting Information

Figure S1 **Variation in the benefit of paternal care for offspring fitness (effect size = Zr_Benefit_).** Colour codings: red, primates; black, birds; blue, fish; green, insects. Bars represent ± 1 SE and the size of the dots is equal to sample size (log(N)). Dashed line is the mean effect size and the grey region is the 95% CI calculated using a Bayesian mixed model (Table S4b).
(TIF)

Figure S2 **Variation in the costs of paternal care for male future reproductive success (Zr_Cost_).** Colour codings: red, primates; black, birds; blue, fish; green, insects. Bars represent ± 1 SE and the size of the dots is equal to sample size (log(N)). Dashed line is the mean effect size and the grey region is the 95% CI calculated using a Bayesian mixed model (Table S5b).
(TIF)

Figure S3 **Funnel plots for (a) r_Adjust_, (b) r_Benefit_, and (c) r_Cost_.** Filled circles indicate actual data points and open circles represent potential missing data points identified by the trim and fill analyses.
(TIF)

Figure S4 **The correspondence between adjustment of care by males (green), the risk cuckoldry (blue), and the costs of paternal care (red) across the phylogeny of birds.** Larger circles represented larger values. Blanks, no data available.
(TIF)

Table S1 **Data used for analysis of r_Adjust_.**
(DOCX)

Table S2 **Data used for analysis of r_Cost_.**
(DOCX)

Table S3 **Data used for analysis of r_Benefit_.**
(DOCX)

Table S4 **Meta-analysis of benefit: methodological effects.**
(DOCX)

Table S5 **Meta-analysis of cost: methodological effects.**
(DOCX)

Table S6 **Meta-analysis of adjustment: methodological effects.**
(DOCX)

Table S7 **Meta-analysis of adjustment: biological effects.**
(DOCX)

Table S8 **Phylogenetic meta-analysis of adjustment of paternal care across just birds.**
(DOCX)

Text S1 **Supplementary results.**
(DOCX)

## Abbreviations

CI: credible interval
SE: standard error
