# Supplementary figures and images for: Why Do Cuckolded Males Provide Paternal Care?
Source: PLoS Biol. 2013 Mar 26;11(3):e1001520. doi: 10.1371/journal.pbio.1001520 (PMC3608547; doi:10.1371/journal.pbio.1001520)

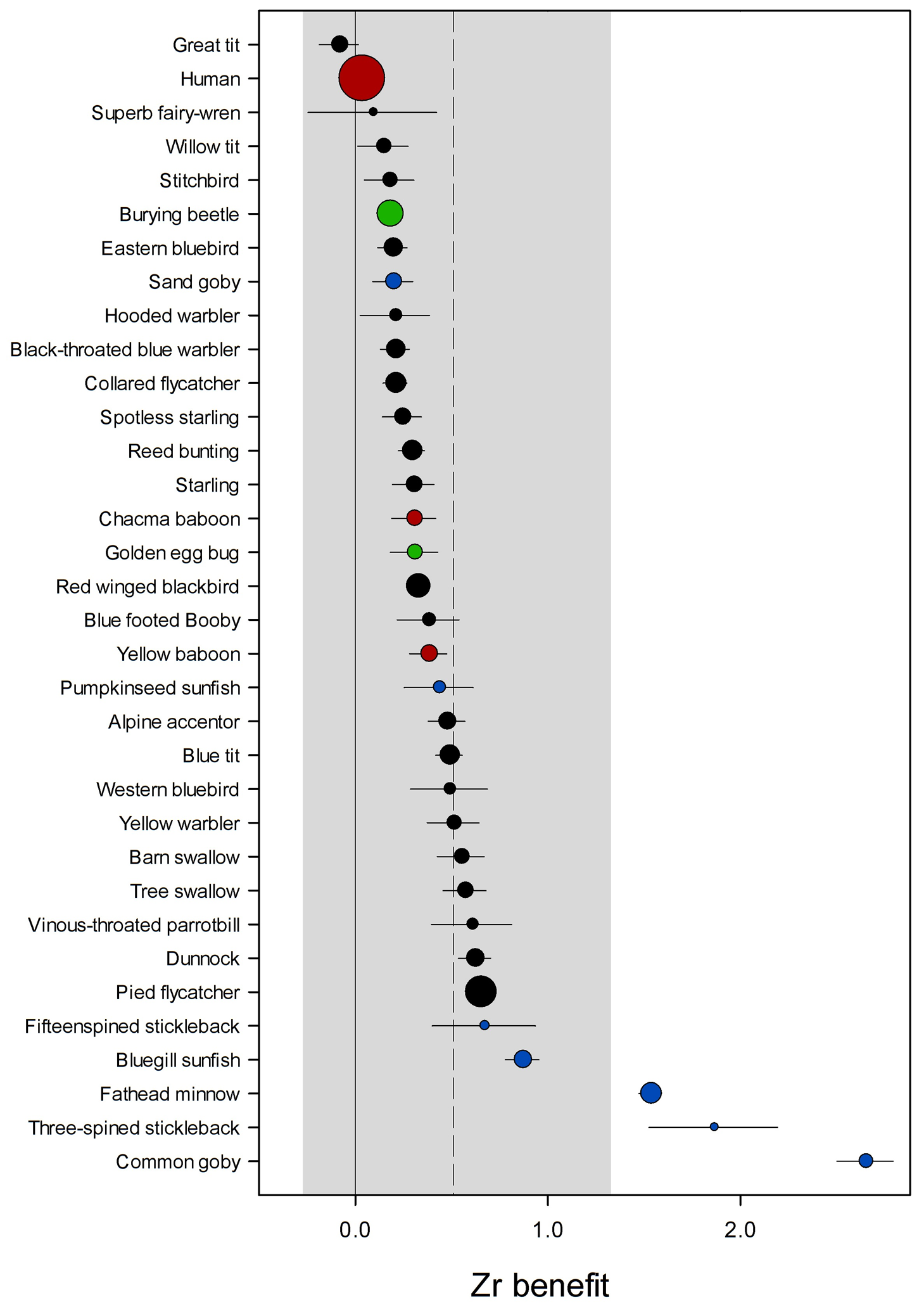

Supplement: Figure S1 — Variation in the benefit of paternal care for offspring fitness (effect size = ZrBenefit). Colour codings: red, primates; black, birds; blue, fish; green, insects. Bars represent ± 1 SE and the size of the dots is equal to sample size (log(N)). Dashed line is the mean effect size and the grey region is the 95% CI calculated using a Bayesian mixed model (Table S4b). (TIF) [file pbio.1001520.s001.tif]

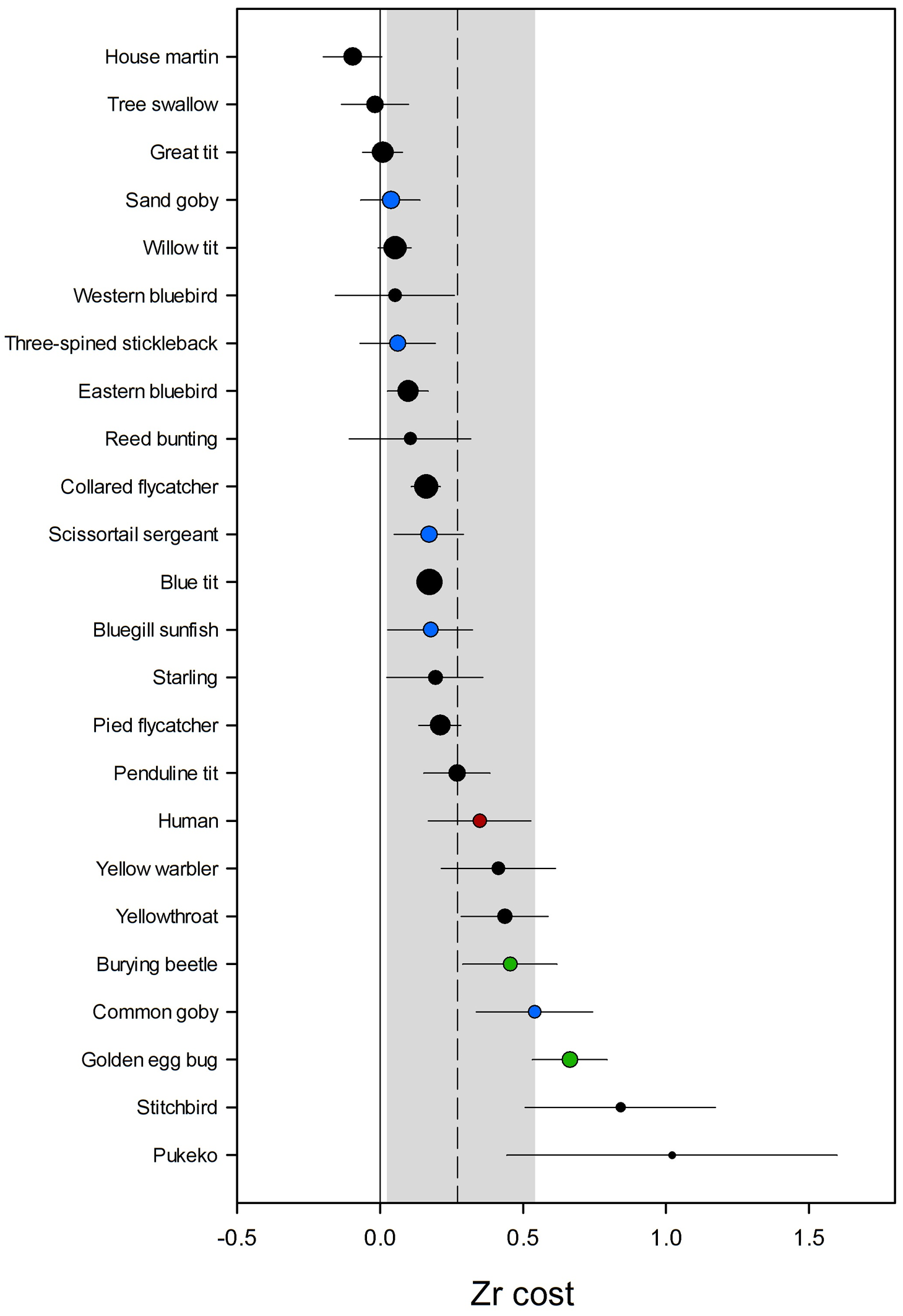

Supplement: Figure S2 — Variation in the costs of paternal care for male future reproductive success (ZrCost). Colour codings: red, primates; black, birds; blue, fish; green, insects. Bars represent ± 1 SE and the size of the dots is equal to sample size (log(N)). Dashed line is the mean effect size and the grey region is the 95% CI calculated using a Bayesian mixed model (Table S5b). (TIF) [file pbio.1001520.s002.tif]

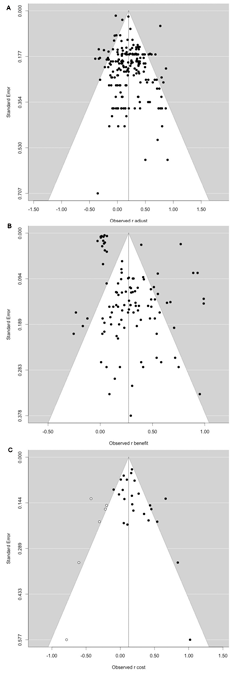

Supplement: Figure S3 — Funnel plots for (a) rAdjust, (b) rBenefit, and (c) rCost. Filled circles indicate actual data points and open circles represent potential missing data points identified by the trim and fill analyses. (TIF) [file pbio.1001520.s003.tif]

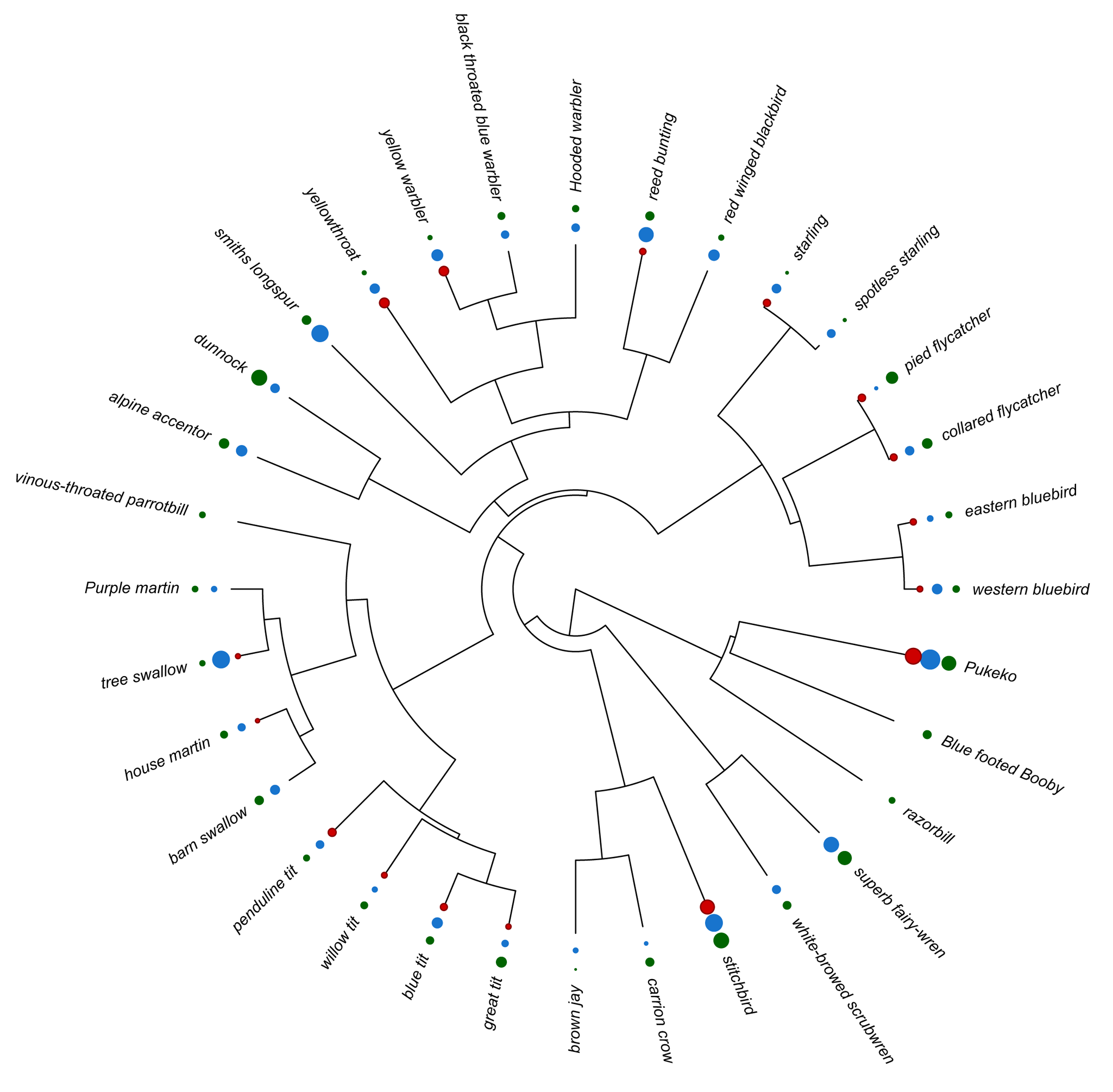

Supplement: Figure S4 — The correspondence between adjustment of care by males (green), the risk cuckoldry (blue), and the costs of paternal care (red) across the phylogeny of birds. Larger circles represented larger values. Blanks, no data available. (TIF) [file pbio.1001520.s004.tif]
