# Supplementary material for: Why Do Cuckolded Males Provide Paternal Care?
Source: PLoS Biol. 2013 Mar 26;11(3):e1001520. doi: 10.1371/journal.pbio.1001520 (PMC3608547; doi:10.1371/journal.pbio.1001520)
Supplement: Table S1 — Data used for analysis of rAdjust. (DOCX) [file pbio.1001520.s005.docx]

**Table S1: Data used for analysis of r_Adjust_**

| Class | Species | Common name | Breeding system | Adjust-ment reported^a^ | Amount or  Probability^b^ | Cues to paternity | | | | Multiple paternity | n | rAdjust | n | Mean rBenefit ^c^ | n | Mean  rCost^d^ | n |
| --- | --- | --- | --- | --- | --- | --- | --- | --- | --- | --- | --- | --- | --- | --- | --- | --- | --- |
|  |  |  |  |  |  | Expt Obs | Genetic data | Mating Access | No.  Males |  |  |  |  |  |  |  |  |
| Actinopterygii | *Abudefduf sexfasciatus* | Scissortail sergeant | Polygynandrous | No [1] | Amount | Expt | No | No | Yes | NA | NA | 0.25 | 27 | NA | NA | 0.17 | 70 |
| Actinopterygii | *Abudefduf sexfasciatus* | Scissortail sergeant | Polygynandrous | No [1] | Prob | Expt | No | No | Yes | NA | NA | 0.38 | 27 | NA | NA | 0.17 | 70 |
| Actinopterygii | *Abudefduf sexfasciatus* | Scissortail sergeant | Polygynandrous | Yes [1] | Amount | Expt | No | No | Yes | NA | NA | 0.50 | 27 | NA | NA | 0.17 | 70 |
| Actinopterygii | *Gasterosteus aculeatus* | Three-spined stickleback | Polygynandrous | No [2] | Prob | Expt | No | No | No | 38.70 [3,4] | 31 | 0.18 | 82 | 0.95 | 12 | 0.06 | 60 |
| Actinopterygii | *Gasterosteus aculeatus* | Three-spined stickleback | Polygynandrous | Yes [2] | Prob | Expt | No | No | No | 38.70 | 31 | 0.27 | 82 | 0.95 | 12 | 0.06 | 60 |
| Actinopterygii | *Lepomis gibbosus* | Pumpkinseed sunfish | Polygynandrous | No [5] | Amount | Obs | Yes | No | No | 93.33 [6] | 15 | -0.17 | 33 | 0.41 | 34 | NA | NA |
| Actinopterygii | *Lepomis gibbosus* | Pumpkinseed sunfish | Polygynandrous | No [5] | Amount | Expt | Yes | No | No | 93.33 | 15 | -0.05 | 18 | 0.41 | 34 | NA | NA |
| Actinopterygii | *Lepomis gibbosus* | Pumpkinseed sunfish | Polygynandrous | No [5] | Amount | Obs | Yes | No | No | 93.33 | 15 | 0.16 | 52 | 0.41 | 34 | NA | NA |
| Actinopterygii | *Lepomis gibbosus* | Pumpkinseed sunfish | Polygynandrous | Yes [5] | Amount | Obs | Yes | No | No | 93.33 | 15 | 0.41 | 33 | 0.41 | 34 | NA | NA |
| Actinopterygii | *Lepomis macrochirus* | Bluegill sunfish | Polygynandrous | No [7] | Amount | Expt | No | No | No | 93.65 [6,8,9] | 126 | 0.03 | 23 | 0.70 | 134 | 0.17 | 48 |
| Actinopterygii | *Lepomis macrochirus* | Bluegill sunfish | Polygynandrous | No [7] | Amount | Expt | No | No | No | 93.65 | 126 | 0.23 | 25 | 0.70 | 134 | 0.17 | 48 |
| Actinopterygii | *Lepomis macrochirus* | Bluegill sunfish | Polygynandrous | No [10] | Prob | Obs | Yes | No | No | 93.65 | 126 | 0.01 | 38 | 0.70 | 134 | 0.17 | 48 |
| Actinopterygii | *Lepomis macrochirus* | Bluegill sunfish | Polygynandrous | No [10] | Prob | Obs | Yes | No | No | 93.65 | 126 | 0.29 | 38 | 0.70 | 134 | 0.17 | 48 |
| Actinopterygii | *Lepomis macrochirus* | Bluegill sunfish | Polygynandrous | Yes [10] | Prob | Obs | Yes | No | No | 93.65 | 126 | 0.45 | 38 | 0.70 | 134 | 0.17 | 48 |
| Actinopterygii | *Lepomis macrochirus* | Bluegill sunfish | Polygynandrous | Yes [10] | Prob | Obs | Yes | No | No | 93.65 | 126 | 0.47 | 38 | 0.70 | 134 | 0.17 | 48 |
| Actinopterygii | *Lepomis macrochirus* | Bluegill sunfish | Polygynandrous | Yes [10] | Prob | Obs | Yes | No | No | 93.65 | 126 | 0.61 | 38 | 0.70 | 134 | 0.17 | 48 |
| Actinopterygii | *Lepomis macrochirus* | Bluegill sunfish | Polygynandrous | Yes [11] | Amount | Expt | Yes | No | No | 93.65 | 126 | 0.37 | 54 | 0.70 | 134 | 0.17 | 48 |
| Actinopterygii | *Lepomis macrochirus* | Bluegill sunfish | Polygynandrous | Yes [11] | Amount | Expt | No | No | Yes | 93.65 | 126 | 0.40 | 54 | 0.70 | 134 | 0.17 | 48 |
| Actinopterygii | *Lepomis macrochirus* | Bluegill sunfish | Polygynandrous | No [12] | Amount | Obs | Yes | No | No | 93.65 | 126 | 0.17 | 38 | 0.70 | 134 | 0.17 | 48 |
| Actinopterygii | *Lepomis macrochirus* | Bluegill sunfish | Polygynandrous | Yes [12] | Amount | Obs | Yes | No | No | 93.65 | 126 | 0.35 | 38 | 0.70 | 134 | 0.17 | 48 |
| Actinopterygii | *Lepomis macrochirus* | Bluegill sunfish | Polygynandrous | Yes [12] | Amount | Obs | Yes | No | No | 93.65 | 126 | 0.49 | 38 | 0.70 | 134 | 0.17 | 48 |
| Actinopterygii | *Pimephales promelas* | Fathead minnow | Polygynandrous | Yes [13] | Prob | Expt | No | No | No | 16.70 [14] | 42 | 0.49 | 24 | 0.91 | 300 | NA | NA |
| Actinopterygii | *Pomatoschistus microps* | Common goby | Polygynandrous | No[15] | Amount | Expt | No | No | Yes | NA | NA | -0.10 | 9 | 0.99 | 50 | 0.49 | 27 |
| Actinopterygii | *Pomatoschistus microps* | Common goby | Polygynandrous | No [15] | Prob | Expt | No | No | Yes | NA | NA | 0.00 | 19 | 0.99 | 50 | 0.49 | 27 |
| Actinopterygii | *Pomatoschistus microps* | Common goby | Polygynandrous | No [15] | Amount | Expt | No | No | Yes | NA | NA | 0.15 | 15 | 0.99 | 50 | 0.49 | 27 |
| Actinopterygii | *Pomatoschistus minutus* | Sand goby | Polygynandrous | No [16] | Prob | Expt | No | No | Yes | 52.20 [17] | 23 | -0.16 | 24 | 0.19 | 96 | 0.04 | 96 |
| Actinopterygii | *Pomatoschistus minutus* | Sand goby | Polygynandrous | No [16] | Prob | Expt | No | No | Yes | 52.20 | 23 | 0.07 | 29 | 0.19 | 96 | 0.04 | 96 |
| Actinopterygii | *Pomatoschistus minutus* | Sand goby | Polygynandrous | No [16] | Prob | Expt | Yes | No | Yes | 52.20 | 23 | 0.10 | 29 | 0.19 | 96 | 0.04 | 96 |
| Actinopterygii | *Pomatoschistus minutus* | Sand goby | Polygynandrous | No [16] | Prob | Expt | No | No | Yes | 52.20 | 23 | 0.19 | 23 | 0.19 | 96 | 0.04 | 96 |
| Actinopterygii | *Pomatoschistus minutus* | Sand goby | Polygynandrous | No [16] | Prob | Expt | No | No | Yes | 52.20 | 23 | 0.34 | 20 | 0.19 | 96 | 0.04 | 96 |
| Actinopterygii | *Spinachia spinachia* | Fifteen-spined  stickleback | Polygynandrous | No [18] | Amount | Expt | No | No | Yes | 13.64 [19] | 44 | -0.12 | 29 | 0.58 | 17 | NA | NA |
| Actinopterygii | *Spinachia spinachia* | Fifteen-spined  stickleback | Polygynandrous | No [18] | Amount | Expt | No | No | Yes | 13.64 | 44 | -0.08 | 29 | 0.58 | 17 | NA | NA |
| Actinopterygii | *Spinachia spinachia* | Fifteen-spined  stickleback | Polygynandrous | No [18] | Amount | Expt | No | No | Yes | 13.64 | 44 | -0.03 | 29 | 0.58 | 17 | NA | NA |
| Actinopterygii | *Spinachia spinachia* | Fifteen-spined  stickleback | Polygynandrous | No [18] | Amount | Expt | No | No | Yes | 13.64 | 44 | 0.01 | 29 | 0.58 | 17 | NA | NA |
| Aves | *Agelaius phoeniceus* | Red-winged blackbird | Polygynous | No [20] | Amount | Obs | Yes | No | No | 49.17 [20,21,22,23,24,25] | 423 | -0.08 | 48 | 0.31 | 613 | NA | NA |
| Aves | *Agelaius phoeniceus* | Red-winged blackbird | Polygynous | No [20] | Amount | Obs | Yes | No | No | 49.17 | 423 | -0.06 | 78 | 0.31 | 613 | NA | NA |
| Aves | *Agelaius phoeniceus* | Red-winged blackbird | Polygynous | No [20] | Amount | Obs | Yes | No | No | 49.17 | 423 | -0.05 | 111 | 0.31 | 613 | NA | NA |
| Aves | *Agelaius phoeniceus* | Red-winged blackbird | Polygynous | No [20] | Amount | Obs | Yes | No | No | 49.17 | 423 | -0.02 | 114 | 0.31 | 613 | NA | NA |
| Aves | *Agelaius phoeniceus* | Red-winged blackbird | Polygynous | No [20] | Amount | Obs | Yes | No | No | 49.17 | 423 | 0.07 | 76 | 0.31 | 613 | NA | NA |
| Aves | *Alca torda* | Razorbill | Pair | No [26] | Amount | Obs | No | No | Yes | NA | NA | -0.10 | 18 | NA | NA | NA | NA |
| Aves | *Alca torda* | Razorbill | Pair | No[26] | Amount | Obs | No | No | Yes | NA | NA | 0.00 | 22 | NA | NA | NA | NA |
| Aves | *Alca torda* | Razorbill | Pair | No [26] | Amount | Obs | No | No | Yes | NA | NA | 0.15 | 24 | NA | NA | NA | NA |
| Aves | *Calcarius pictus* | Smith’s longspur | Polygynandrous | No [27] | Amount | Obs | Yes | No | No | 77.42 [27] | 13 | 0.07 | 10 | NA | NA | NA | NA |
| Aves | *Calcarius pictus* | Smith’s longspur | Polygynandrous | No [27] | Amount | Obs | Yes | No | No | 77.42 | 13 | 0.07 | 10 | NA | NA | NA | NA |
| Aves | *Calcarius pictus* | Smith’s longspur | Polygynandrous | No [27] | Prob | Obs | Yes | No | No | 77.42 | 13 | 0.14 | 39 | NA | NA | NA | NA |
| Aves | *Calcarius pictus* | Smith’s longspur | Polygynandrous | No [27] | Amount | Obs | Yes | No | No | 77.42 | 13 | 0.14 | 10 | NA | NA | NA | NA |
| Aves | *Calcarius pictus* | Smith’s longspur | Polygynandrous | No [27] | Amount | Obs | Yes | No | No | 77.42 | 13 | 0.21 | 38 | NA | NA | NA | NA |
| Aves | *Calcarius pictus* | Smith’s longspur | Polygynandrous | No [27] | Amount | Obs | Yes | No | No | 77.42 | 13 | 0.25 | 29 | NA | NA | NA | NA |
| Aves | *Calcarius pictus* | Smith’s longspur | Polygynandrous | No [27] | Amount | Obs | Yes | No | No | 77.42 | 13 | 0.25 | 29 | NA | NA | NA | NA |
| Aves | *Calcarius pictus* | Smith’s longspur | Polygynandrous | Yes [27] | Amount | Obs | Yes | No | No | 77.42 | 13 | 0.27 | 38 | NA | NA | NA | NA |
| Aves | *Calcarius pictus* | Smith’s longspur | Polygynandrous | Yes [27] | Amount | Obs | Yes | No | No | 77.42 | 13 | 0.27 | 39 | NA | NA | NA | NA |
| Aves | *Calcarius pictus* | Smith’s longspur | Polygynandrous | Yes [27] | Amount | Obs | Yes | No | No | 77.42 | 13 | 0.31 | 38 | NA | NA | NA | NA |
| Aves | *Calcarius pictus* | Smith’s longspur | Polygynandrous | Yes [27] | Amount | Obs | Yes | No | No | 77.42 | 13 | 0.32 | 39 | NA | NA | NA | NA |
| Aves | *Calcarius pictus* | Smith’s longspur | Polygynandrous | [27] | Amount | Obs | Yes | No | No | 77.42 | 13 | 0.33 | 39 | NA | NA | NA | NA |
| Aves | *Calcarius pictus* | Smith’s longspur | Polygynandrous | Yes [27] | Amount | Obs | Yes | No | No | 77.42 | 13 | 0.34 | 29 | NA | NA | NA | NA |
| Aves | *Calcarius pictus* | Smith’s longspur | Polygynandrous | Yes [27] | Amount | Obs | Yes | No | No | 77.42 | 13 | 0.35 | 38 | NA | NA | NA | NA |
| Aves | *Calcarius pictus* | Smith’s longspur | Polygynandrous | Yes [27] | Amount | Obs | Yes | No | No | 77.42 | 13 | 0.36 | 29 | NA | NA | NA | NA |
| Aves | *Calcarius pictus* | Smith’s longspur | Polygynandrous | Yes [27] | Amount | Obs | Yes | No | No | 77.42 | 13 | 0.37 | 29 | NA | NA | NA | NA |
| Aves | *Calcarius pictus* | Smith’s longspur | Polygynandrous | Yes [27] | Amount | Obs | Yes | No | No | 77.42 | 13 | 0.40 | 29 | NA | NA | NA | NA |
| Aves | *Calcarius pictus* | Smith’s longspur | Polygynandrous | Yes [27] | Amount | Obs | Yes | No | No | 77.42 | 13 | 0.55 | 11 | NA | NA | NA | NA |
| Aves | *Calcarius pictus* | Smith’s longspur | Polygynandrous | Yes [27] | Amount | Obs | Yes | No | No | 77.42 | 13 | 0.58 | 11 | NA | NA | NA | NA |
| Aves | *Corvus corone* | Carrion crow | Cooperative | No [28] | Amount | Obs | Yes | No | No | 15.79 [29,30] | 19 | 0.25 | 30 | NA | NA | NA | NA |
| Aves | *Cyanistes caeruleus* | Blue tit | Pair | No [31] | Amount | Obs | Yes | No | No | 47.68 [32,33,34,35,36,37,38,39,40,41,42] | 690 | 0.18 | 22 | 0.45 | 214 | 0.17 | 673 |
| Aves | *Cyanocorax morio* | Brown jay | Cooperative | No [43] | Amount | Obs | Yes | No | No | 22.22 [44] | 18 | -0.35 | 5 | NA | NA | NA | NA |
| Aves | *Delichon urbica* | House martin | Pair | No [45] | Amount | Obs | Yes | No | No | 33.33 [46,47] | 39 | -0.07 | 10 | NA | NA | -0.10 | 98 |
| Aves | *Delichon urbica* | House martin | Pair | No [45] | Amount | Obs | Yes | No | No | 33.33 | 39 | -0.03 | 10 | NA | NA | -0.10 | 98 |
| Aves | *Delichon urbica* | House martin | Pair | No [45] | Amount | Obs | Yes | No | No | 33.33 | 39 | 0.01 | 10 | NA | NA | -0.10 | 98 |
| Aves | *Delichon urbica* | House martin | Pair | No [45] | Amount | Obs | Yes | No | No | 33.33 | 39 | 0.20 | 10 | NA | NA | -0.10 | 98 |
| Aves | *Delichon urbica* | House martin | Pair | No [45] | Amount | Obs | Yes | No | No | 33.33 | 39 | 0.31 | 10 | NA | NA | -0.10 | 98 |
| Aves | *Delichon urbica* | House martin | Pair | No [45] | Amount | Obs | Yes | No | No | 33.33 | 39 | 0.41 | 10 | NA | NA | -0.10 | 98 |
| Aves | *Dendroica caerulescens* | Black-throated  blue warbler | Pair | No [48] | Amount | Obs | Yes | No | No | 34.19 [49] | 117 | -0.23 | 24 | 0.20 | 182 | NA | NA |
| Aves | *Dendroica caerulescens* | Black-throated  blue warbler | Pair | No [48] | Amount | Obs | Yes | No | No | 34.19 | 117 | -0.13 | 19 | 0.20 | 182 | NA | NA |
| Aves | *Dendroica caerulescens* | Black-throated  blue warbler | Pair | No [48] | Amount | Obs | Yes | No | No | 34.19 | 117 | 0.01 | 10 | 0.20 | 182 | NA | NA |
| Aves | *Dendroica caerulescens* | Black-throated  blue warbler | Pair | No [48] | Amount | Obs | Yes | No | No | 34.19 | 117 | 0.08 | 51 | 0.20 | 182 | NA | NA |
| Aves | *Dendroica caerulescens* | Black-throated  blue warbler | Pair | No [48] | Amount | Obs | Yes | No | No | 34.19 | 117 | 0.10 | 45 | 0.20 | 182 | NA | NA |
| Aves | *Dendroica caerulescens* | Black-throated  blue warbler | Pair | No [48] | Amount | Obs | Yes | No | No | 34.19 | 117 | 0.16 | 9 | 0.20 | 182 | NA | NA |
| Aves | *Dendroica caerulescens* | Black-throated  blue warbler | Pair | No [48] | Amount | Obs | Yes | No | No | 34.19 | 117 | 0.29 | 19 | 0.20 | 182 | NA | NA |
| Aves | *Dendroica caerulescens* | Black-throated  blue warbler | Pair | No [48] | Amount | Obs | Yes | No | No | 34.19 | 117 | 0.34 | 22 | 0.20 | 182 | NA | NA |
| Aves | *Dendroica caerulescens* | Black-throated  blue warbler | Pair | Yes [48] | Amount | Obs | Yes | No | No | 34.19 | 117 | 0.34 | 30 | 0.20 | 182 | NA | NA |
| Aves | *Dendroica caerulescens* | Black-throated  blue warbler | Pair | Yes [48] | Amount | Obs | Yes | No | No | 34.19 | 117 | 0.38 | 29 | 0.20 | 182 | NA | NA |
| Aves | *Dendroica petechia* | Yellow warbler | Pair | No [50] | Amount | Obs | Yes | No | No | 51.41 [50,51,52] | 142 | -0.19 | 31 | 0.47 | 58 | 0.39 | 28 |
| Aves | *Dendroica petechia* | Yellow warbler | Pair | No [50] | Amount | Obs | Yes | No | No | 51.41 | 142 | -0.16 | 31 | 0.47 | 58 | 0.39 | 28 |
| Aves | *Dendroica petechia* | Yellow warbler | Pair | No [50] | Amount | Obs | Yes | No | No | 51.41 | 142 | -0.16 | 31 | 0.47 | 58 | 0.39 | 28 |
| Aves | *Dendroica petechia* | Yellow warbler | Pair | No [50] | Amount | Obs | Yes | No | No | 51.41 | 142 | 0.03 | 31 | 0.47 | 58 | 0.39 | 28 |
| Aves | *Emberiza schoeniclus* | Reed bunting | Pair | No [53] | Amount | Obs | Yes | No | No | 67.24 [54,55,56,57] | 312 | -0.16 | 31 | 0.28 | 224 | 0.10 | 25 |
| Aves | *Emberiza schoeniclus* | Reed bunting | Pair | No [53] | Amount | Obs | Yes | No | No | 67.24 | 312 | -0.09 | 10 | 0.28 | 224 | 0.10 | 25 |
| Aves | *Emberiza schoeniclus* | Reed bunting | Pair | No [53] | Amount | Obs | Yes | No | No | 67.24 | 312 | -0.03 | 12 | 0.28 | 224 | 0.10 | 25 |
| Aves | *Emberiza schoeniclus* | Reed bunting | Pair | No [53] | Amount | Obs | Yes | No | No | 67.24 | 312 | 0.02 | 31 | 0.28 | 224 | 0.10 | 25 |
| Aves | *Emberiza schoeniclus* | Reed bunting | Pair | No [53] | Amount | Obs | Yes | No | No | 67.24 | 312 | 0.11 | 10 | 0.28 | 224 | 0.10 | 25 |
| Aves | *Emberiza schoeniclus* | Reed bunting | Pair | Yes [55] | Amount | Obs | Yes | No | No | 67.24 | 312 | 0.79 | 12 | 0.28 | 224 | 0.10 | 25 |
| Aves | *Emberiza schoeniclus* | Reed bunting | Pair | Yes [58] | Amount | Obs | Yes | No | No | 67.24 | 312 | 0.26 | 51 | 0.28 | 224 | 0.10 | 25 |
| Aves | *Emberiza schoeniclus* | Reed bunting | Pair | Yes [58] | Amount | Obs | Yes | No | No | 67.24 | 312 | 0.71 | 14 | 0.28 | 224 | 0.10 | 25 |
| Aves | *Emberiza schoeniclus* | Reed bunting | Pair | Yes [58] | Amount | Obs | Yes | No | No | 67.24 | 312 | 0.74 | 10 | 0.28 | 224 | 0.10 | 25 |
| Aves | *Emberiza schoeniclus* | Reed bunting | Pair | Yes [58] | Amount | Obs | Yes | No | No | 67.24 | 312 | 0.80 | 10 | 0.28 | 224 | 0.10 | 25 |
| Aves | *Emberiza schoeniclus* | Reed bunting | Pair | Yes [58] | Amount | Obs | Yes | No | No | 67.24 | 312 | 0.86 | 10 | 0.28 | 224 | 0.10 | 25 |
| Aves | *Ficedula albicollis* | Collared flycatcher | Pair | Yes [59] | Amount | Expt | No | Yes | No | 39.35 [60,61,62,63] | 216 | 0.34 | 53 | 0.20 | 265 | 0.16 | 398 |
| Aves | *Ficedula albicollis* | Collared flycatcher | Pair | Yes [59] | Amount | Expt | No | Yes | No | 39.35 | 216 | 0.36 | 53 | 0.20 | 265 | 0.16 | 398 |
| Aves | *Ficedula albicollis* | Collared flycatcher | Pair | Yes [59] | Amount | Expt | No | Yes | No | 39.35 | 216 | 0.36 | 53 | 0.20 | 265 | 0.16 | 398 |
| Aves | *Ficedula albicollis* | Collared flycatcher | Pair | Yes [59] | Amount | Expt | No | Yes | No | 39.35 | 216 | 0.37 | 53 | 0.20 | 265 | 0.16 | 398 |
| Aves | *Ficedula albicollis* | Collared flycatcher | Pair | Yes [64] | Amount | Expt | No | Yes | No | 39.35 | 216 | 0.34 | 48 | 0.20 | 265 | 0.16 | 398 |
| Aves | *Ficedula albicollis* | Collared flycatcher | Pair | No [64] | Amount | Expt | No | Yes | No | 39.35 | 216 | 0.38 | 21 | 0.20 | 265 | 0.16 | 398 |
| Aves | *Ficedula albicollis* | Collared flycatcher | Pair | Yes [64] | Amount | Expt | No | Yes | No | 39.35 | 216 | 0.38 | 48 | 0.20 | 265 | 0.16 | 398 |
| Aves | *Ficedula albicollis* | Collared flycatcher | Pair | Yes [64] | Amount | Expt | No | Yes | No | 39.35 | 216 | 0.40 | 27 | 0.20 | 265 | 0.16 | 398 |
| Aves | *Ficedula albicollis* | Collared flycatcher | Pair | Yes [64] | Amount | Expt | No | Yes | No | 39.35 | 216 | 0.47 | 21 | 0.20 | 265 | 0.16 | 398 |
| Aves | *Ficedula albicollis* | Collared flycatcher | Pair | Yes [64] | Amount | Expt | No | Yes | No | 39.35 | 216 | 0.47 | 27 | 0.20 | 265 | 0.16 | 398 |
| Aves | *Ficedula hypoleuca* | Pied flycatcher | Pair | Yes [65] | Amount | Expt | Yes | Yes | No | 13.91 [66,67,68,69,70,71,72,73] | 460 | 0.51 | 17 | 0.57 | 3999 | 0.21 | 184 |
| Aves | *Geothlypis trichas* | Yellowthroat | Pair | No [74] | Amount | Obs | Yes | No | No | 43.48 [75] | 138 | -0.33 | 32 | NA | NA | 0.41 | 46 |
| Aves | *Geothlypis trichas* | Yellowthroat | Pair | No [74] | Amount | Obs | Yes | No | No | 43.48 | 138 | -0.31 | 33 | NA | NA | 0.41 | 46 |
| Aves | *Geothlypis trichas* | Yellowthroat | Pair | No [74] | Amount | Obs | Yes | No | No | 43.48 | 138 | 0.37 | 12 | NA | NA | 0.41 | 46 |
| Aves | *Geothlypis trichas* | Yellowthroat | Pair | No [74] | Amount | Obs | Yes | No | No | 43.48 | 138 | 0.37 | 12 | NA | NA | 0.41 | 46 |
| Aves | *Hirundo rustica* | Barn swallow | Pair | No [76] | Amount | Obs | Yes | No | No | 42.22 [77,78,79,80] | 334 | -0.40 | 32 | 0.50 | 70 | NA | NA |
| Aves | *Hirundo rustica* | Barn swallow | Pair | No [79] | Amount | Obs | Yes | No | No | 42.22 | 334 | 0.00 | 47 | 0.50 | 70 | NA | NA |
| Aves | *Hirundo rustica* | Barn swallow | Pair | No [79] | Amount | Obs | Yes | No | No | 42.22 | 334 | 0.23 | 47 | 0.50 | 70 | NA | NA |
| Aves | *Hirundo rustica* | Barn swallow | Pair | Yes [79] | Amount | Obs | Yes | No | No | 42.22 | 334 | 0.90 | 6 | 0.50 | 70 | NA | NA |
| Aves | *Hirundo rustica* | Barn swallow | Pair | Yes [81] | Amount | Expt | No | Yes | No | 42.22 | 334 | 0.34 | 38 | 0.50 | 70 | NA | NA |
| Aves | *Hirundo rustica* | Barn swallow | Pair | Yes [81] | Amount | Expt | No | Yes | No | 42.22 | 334 | 0.37 | 38 | 0.50 | 70 | NA | NA |
| Aves | *Hirundo rustica* | Barn swallow | Pair | Yes [81] | Amount | Expt | No | Yes | No | 42.22 | 334 | 0.56 | 38 | 0.50 | 70 | NA | NA |
| Aves | *Hirundo rustica* | Barn swallow | Pair | Yes [81] | Amount | Expt | No | Yes | No | 42.22 | 334 | 0.68 | 38 | 0.50 | 70 | NA | NA |
| Aves | *Malurus cyaneus* | Superb fairy-wren | Cooperative | Yes [82] | Amount | Obs | Yes | No | No | 69.28 [83] | 293 | 0.63 | 16 | 0.09 | 12 | NA | NA |
| Aves | *Notiomystis cincta* | Stitchbird | Polygynandrous | Yes [84] | Amount | Obs | No | No | Yes | 80.00 [85,86] | 71 | 0.72 | 7 | 0.17 | 64 | 0.69 | 12 |
| Aves | *Paradoxornis webbianus* | Vinous-throated parrotbill | Pair | No [87] | Amount | Obs | Yes | No | No | 26.00 [88] | 50 | 0.03 | 23 | 0.54 | 26 | NA | NA |
| Aves | *Parus major* | Great tit | Pair | Yes [89] | Amount | Obs | Yes | No | No | 29.86 [37,39,90,91,92] | 422 | 0.41 | 16 | -0.09 | 100 | 0.01 | 202 |
| Aves | *Poecile montanus* | Willow tit | Pair | No [93] | Amount | Obs | Yes | No | No | 23.44 [93,94] | 64 | 0.08 | 36 | 0.14 | 62 | 0.05 | 296 |
| Aves | *Poecile montanus* | Willow tit | Pair | No [93] | Amount | Obs | Yes | No | No | 23.44 | 64 | 0.09 | 39 | 0.14 | 62 | 0.05 | 296 |
| Aves | *Poecile montanus* | Willow tit | Pair | No [93] | Amount | Obs | Yes | No | No | 23.44 | 64 | 0.23 | 39 | 0.14 | 62 | 0.05 | 296 |
| Aves | *Porphyrio porphyrio* | Pukeko | Polygynandrous | No [95] | Amount | Obs | Yes | No | No | 91.67 [95] | 12 | 0.50 | 6 | NA | NA | 0.77 | 6 |
| Aves | *Porphyrio porphyrio* | Pukeko | Polygynandrous | Yes [95] | Amount | Obs | Yes | No | No | 91.67 | 12 | 0.80 | 8 | NA | NA | 0.77 | 6 |
| Aves | *Progne subis* | Purple martin | Pair | No [96] | Amount | Obs | Yes | No | No | 24.39 [97,98] | 41 | 0.04 | 15 | NA | NA | NA | NA |
| Aves | *Progne subis* | Purple martin | Pair | No [96] | Amount | Obs | Yes | No | No | 24.39 | 41 | 0.16 | 15 | NA | NA | NA | NA |
| Aves | *Progne subis* | Purple martin | Pair | No [96] | Amount | Obs | Yes | No | No | 24.39 | 41 | -0.15 | 15 | NA | NA | NA | NA |
| Aves | *Prunella collaris* | Alpine accentor | Polygynandrous | No [99] | Amount | Obs | Yes | No | No | 48.84 [99,100] | 43 | 0.11 | 24 | 0.44 | 114 | NA | NA |
| Aves | *Prunella collaris* | Alpine accentor | Polygynandrous | No [99] | Amount | Obs | No | Yes | No | 48.84 | 43 | 0.39 | 20 | 0.44 | 114 | NA | NA |
| Aves | *Prunella collaris* | Alpine accentor | Polygynandrous | Yes [99] | Prob | Obs | No | Yes | No | 48.84 | 43 | 0.75 | 14 | 0.44 | 114 | NA | NA |
| Aves | *Prunella modularis* | Dunnock | Polygynandrous | Yes [101] | Amount | Expt | No | Yes | No | 40.00 [102] | 45 | 0.55 | 17 | 0.55 | 145 | NA | NA |
| Aves | *Prunella modularis* | Dunnock | Polygynandrous | Yes [101] | Amount | Expt | No | Yes | No | 40.00 | 45 | 0.67 | 17 | 0.55 | 145 | NA | NA |
| Aves | *Prunella modularis* | Dunnock | Polygynandrous | Yes [101] | Amount | Expt | No | Yes | No | 40.00 | 45 | 0.70 | 17 | 0.55 | 145 | NA | NA |
| Aves | *Prunella modularis* | Dunnock | Polygynandrous | Yes [101] | Prob | Expt | Yes | No | No | 40.00 | 45 | 0.79 | 18 | 0.55 | 145 | NA | NA |
| Aves | *Prunella modularis* | Dunnock | Polygynandrous | Yes [101] | Amount | Expt | No | Yes | No | 40.00 | 45 | 0.87 | 23 | 0.55 | 145 | NA | NA |
| Aves | *Remiz pendulinus* | Penduline tit | Pair | No [103] | Prob | Obs | Yes | No | No | 35.51 [103,104] | 107 | 0.05 | 114 | NA | NA | 0.26 | 78 |
| Aves | *Sericornis frontalis* | White-browed scrubwren | Cooperative | No [105] | Amount | Obs | Yes | No | No | 37.25 [106] | 32 | -0.12 | 26 | NA | NA | NA | NA |
| Aves | *Sericornis frontalis* | White-browed scrubwren | Polygynandrous | No [105] | Amount | Obs | Yes | No | No | 37.25 | 32 | 0.05 | 15 | NA | NA | NA | NA |
| Aves | *Sericornis frontalis* | White-browed scrubwren | Polygynandrous | Yes [105] | Amount | Obs | Yes | No | No | 37.25 | 32 | 0.38 | 26 | NA | NA | NA | NA |
| Aves | *Sericornis frontalis* | White-browed scrubwren | Polygynandrous | No [105] | Amount | Obs | Yes | No | No | 37.25 | 32 | 0.40 | 11 | NA | NA | NA | NA |
| Aves | *Sericornis frontalis* | White-browed scrubwren | Polygynandrous | Yes [105] | Amount | Obs | Yes | No | No | 37.25 | 32 | 0.53 | 16 | NA | NA | NA | NA |
| Aves | *Sialia mexicana* | Western bluebird | Cooperative | No [107] | Amount | Expt | Yes | No | No | 45.10 [108] | 51 | 0.13 | 8 | 0.45 | 28 | 0.05 | 26 |
| Aves | *Sialia mexicana* | Western bluebird | Cooperative | No [109] | Amount | Expt | No | Yes | No | 45.10 | 51 | -0.11 | 18 | 0.45 | 28 | 0.05 | 26 |
| Aves | *Sialia mexicana* | Western bluebird | Cooperative | No [109] | Amount | Expt | Yes | No | No | 45.10 | 51 | 0.00 | 42 | 0.45 | 28 | 0.05 | 26 |
| Aves | *Sialia mexicana* | Western bluebird | Cooperative | No [109] | Amount | Expt | No | Yes | No | 45.10 | 51 | 0.01 | 19 | 0.45 | 28 | 0.05 | 26 |
| Aves | *Sialia mexicana* | Western bluebird | Cooperative | No [109] | Amount | Expt | No | Yes | No | 45.10 | 51 | 0.09 | 30 | 0.45 | 28 | 0.05 | 26 |
| Aves | *Sialia mexicana* | Western bluebird | Cooperative | No [109] | Amount | Expt | Yes | No | No | 45.10 | 51 | 0.16 | 42 | 0.45 | 28 | 0.05 | 26 |
| Aves | *Sialia mexicana* | Western bluebird | Cooperative | No [109] | Amount | Expt | No | Yes | No | 45.10 | 51 | 0.19 | 28 | 0.45 | 28 | 0.05 | 26 |
| Aves | *Sialia mexicana* | Western bluebird | Cooperative | No [109] | Amount | Expt | No | Yes | No | 45.10 | 51 | 0.27 | 28 | 0.45 | 28 | 0.05 | 26 |
| Aves | *Sialia sialis* | Eastern bluebird | Pair | No [110] | Amount | Expt | No | Yes | No | 26.00 [111,112] | 100 | -0.11 | 15 | 0.19 | 177 | 0.10 | 200 |
| Aves | *Sialia sialis* | Eastern bluebird | Pair | No [110] | Amount | Expt | No | Yes | No | 26.00 | 100 | -0.03 | 15 | 0.19 | 177 | 0.10 | 200 |
| Aves | *Sialia sialis* | Eastern bluebird | Pair | No [113] | Amount | Expt | No | Yes | No | 26.00 | 100 | -0.08 | 21 | 0.19 | 177 | 0.10 | 200 |
| Aves | *Sialia sialis* | Eastern bluebird | Pair | No [113] | Amount | Expt | No | Yes | No | 26.00 | 100 | 0.08 | 38 | 0.19 | 177 | 0.10 | 200 |
| Aves | *Sialia sialis* | Eastern bluebird | Pair | No [113] | Amount | Expt | Yes | No | No | 26.00 | 100 | 0.12 | 21 | 0.19 | 177 | 0.10 | 200 |
| Aves | *Sialia sialis* | Eastern bluebird | Pair | No [113] | Amount | Expt | No | Yes | No | 26.00 | 100 | 0.16 | 38 | 0.19 | 177 | 0.10 | 200 |
| Aves | *Sturnus unicolor* | Spotless starling | Polygynous | No [114] | Amount | Obs | Yes | No | No | 36.49 [114,115,116] | 211 | -0.33 | 48 | 0.24 | 101 | NA | NA |
| Aves | *Sturnus unicolor* | Spotless starling | Polygynous | No [114] | Amount | Obs | Yes | No | No | 36.49 | 211 | 0.00 | 15 | 0.24 | 101 | NA | NA |
| Aves | *Sturnus unicolor* | Spotless starling | Polygynous | No [114] | Amount | Obs | Yes | No | No | 36.49 | 211 | 0.01 | 8 | 0.24 | 101 | NA | NA |
| Aves | *Sturnus vulgaris* | Starling | Polygynous | No [117] | Amount | Obs | No | Yes | No | 40.40 [118,119,120] | 99 | -0.31 | 17 | 0.29 | 89 | 0.19 | 38 |
| Aves | *Sturnus vulgaris* | Starling | Polygynous | No [117] | Amount | Obs | No | Yes | No | 40.40 | 99 | -0.15 | 8 | 0.29 | 89 | 0.19 | 38 |
| Aves | *Sula nebouxii* | Blue-footed Booby | Pair | No [121] | Amount | Expt | No | Yes | No | NA | NA | 0.06 | 13 | 0.36 | 42 | NA | NA |
| Aves | *Sula nebouxii* | Blue-footed Booby | Pair | No [121] | Amount | Expt | No | Yes | No | NA | NA | 0.06 | 22 | 0.36 | 42 | NA | NA |
| Aves | *Sula nebouxii* | Blue-footed Booby | Pair | No [121] | Amount | Expt | No | Yes | No | NA | NA | 0.19 | 19 | 0.36 | 42 | NA | NA |
| Aves | *Sula nebouxii* | Blue-footed Booby | Pair | Yes [121] | Prob | Expt | No | Yes | No | NA | NA | 0.47 | 33 | 0.36 | 42 | NA | NA |
| Aves | *Tachycineta bicolor* | Tree swallow | Pair | No [110] | Amount | Expt | No | Yes | No | 79.94 [122,123,124,125,126] | 319 | -0.24 | 46 | 0.51 | 83 | -0.02 | 75 |
| Aves | *Tachycineta bicolor* | Tree swallow | Pair | No [110] | Amount | Expt | No | Yes | No | 79.94 | 319 | -0.16 | 43 | 0.51 | 83 | -0.02 | 75 |
| Aves | *Tachycineta bicolor* | Tree swallow | Pair | No [127] | Amount | Expt | No | Yes | No | 79.94 | 319 | -0.14 | 27 | 0.51 | 83 | -0.02 | 75 |
| Aves | *Tachycineta bicolor* | Tree swallow | Pair | No[127] | Amount | Expt | No | Yes | No | 79.94 | 319 | 0.03 | 27 | 0.51 | 83 | -0.02 | 75 |
| Aves | *Tachycineta bicolor* | Tree swallow | Pair | No[127] | Amount | Expt | No | Yes | No | 79.94 | 319 | 0.07 | 27 | 0.51 | 83 | -0.02 | 75 |
| Aves | *Tachycineta bicolor* | Tree swallow | Pair | No[127] | Amount | Expt | No | Yes | No | 79.94 | 319 | 0.08 | 27 | 0.51 | 83 | -0.02 | 75 |
| Aves | *Tachycineta bicolor* | Tree swallow | Pair | No[127] | Amount | Expt | No | Yes | No | 79.94 | 319 | 0.10 | 25 | 0.51 | 83 | -0.02 | 75 |
| Aves | *Tachycineta bicolor* | Tree swallow | Pair | No[127] | Amount | Expt | No | Yes | No | 79.94 | 319 | 0.38 | 25 | 0.51 | 83 | -0.02 | 75 |
| Aves | *Turdoides squamiceps* | Arabian babbler | Cooperative | No [128] | Amount | Obs | No | No | Yes | 9.09 [129] | 44 | 0.15 | 21 | NA | NA | NA | NA |
| Aves | *Turdoides squamiceps* | Arabian babbler | Cooperative | No [128] | Amount | Obs | Yes | No | No | 9.09 | 44 | 0.20 | 21 | NA | NA | NA | NA |
| Aves | *Wilsonia citrina* | Hooded warbler | Pair | No [130] | Amount | Obs | Yes | No | No | 35.30 [130] | 119 | 0.08 | 12 | 0.20 | 34 | NA | NA |
| Aves | *Wilsonia citrina* | Hooded warbler | Pair | No [130] | Amount | Obs | Yes | No | No | 35.30 | 119 | -0.09 | 12 | 0.20 | 34 | NA | NA |
| Insecta | *Nicrophorus tomentosus* | Burying beetle | Polygynandrous | No [131] | Amount | Obs | Yes | No | No | 70.00 [131] | 10 | 0.14 | 10 | 0.17 | 1170 | 0.43 | 40 |
| Insecta | *Nicrophorus tomentosus* | Burying beetle | Polygynandrous | Yes [131] | Amount | Obs | Yes | No | No | 70.00 | 10 | 0.77 | 10 | 0.17 | 1170 | 0.43 | 40 |
| Insecta | *Phyllomorpha laciniata* | Golden egg bug | Polygynandrous | Yes [132] | Prob | Obs | Yes | No | No | 97.50 [133] | 40 | 0.77 | 293 | 0.30 | 69 | 0.58 | 61 |
| Mammalia | *Homo sapiens* | Human | Pair | Yes [134] | Amount | Obs | No | No | No | 6.16 [135,136] | 12059 | 0.29 | 60 | 0.03 | 183106 | 0.33 | 34 |
| Mammalia | *Homo sapiens* | Human | Pair | Yes [134] | Amount | Obs | No | No | No | 6.16 | 12059 | 0.36 | 60 | 0.03 | 183106 | 0.33 | 34 |
| Mammalia | *Homo sapiens* | Human | Pair | Yes [137] | Amount | Obs | No | No | No | 6.16 | 12059 | 0.23 | 206 | 0.03 | 183106 | 0.33 | 34 |
| Mammalia | *Homo sapiens* | Human | Pair | Yes [138] | Amount | Obs | No | Yes | Yes | 6.16 | 12059 | 0.20 | 1984 | 0.03 | 183106 | 0.33 | 34 |
| Mammalia | *Homo sapiens* | Human | Pair | Yes [139] | Amount | Obs | No | No | No | 6.16 | 12059 | 0.35 | 32 | 0.03 | 183106 | 0.33 | 34 |
| Mammalia | *Macaca sylvanus* | Barbary macaque | Polygynandrous | No[140] | Amount | Obs | Yes | No | No | 86.67 [141,142] | 25 | -0.03 | 2781 | NA | NA | NA | NA |
| Mammalia | *Macaca sylvanus* | Barbary macaque | Polygynandrous | No [143] | Amount | Obs | Yes | No | No | 86.67 | 25 | 0.09 | 816 | NA | NA | NA | NA |
| Mammalia | *Papio cynocephalus* | Yellow baboon | Polygynandrous | No [144] | Amount | Obs | Yes | No | No | 86.32 [145] | 205 | 0.31 | 23 | 0.36 | 111 | NA | NA |
| Mammalia | *Papio ursinus* | Chacma baboon | Polygynandrous | Yes [146] | Amount | Obs | No | Yes | No | 87.87 [147,148] | 37 | 0.82 | 16 | 0.29 | 80 | NA | NA |
| Reptilia | *Egernia whitii* | White's skink | Pair | Yes [149] | Prob | Obs | Yes | No | No | 34.07 [149] | 91 | 0.58 | 39 | NA | NA | NA | NA |

a. Indicates whether or not the source study found a significant (P>0.05) positive relationship between paternity and paternal care; reference from which rAdjust was calculated.

b. Did the source study measure how paternity effected the probability that a male would provide paternal care or did they measure how paternity effected the amount of care provided

c. See Table S2 for references

d. See Table S3 for references

**References for Table S1**

1. Manica A (2004) Parental fish change their cannibalistic behaviour in response to the cost-to-benefit ratio of parental care. Anim. Behav. 67: 1015-1021.

2. Mehlis M, Bakker T, Engqvist L, Frommen J (2010) To eat or not to eat: egg-based assessment of paternity triggers fine-tuned decisions about filial cannibalism. Proc. R. Soc., Ser. B 277: 2627-2635.

3. Rico C, Kuhnlein U, Fitzgerald GJ (1992) Male reproductive tactics in the threespine stickleback-an evaluation by DNA fingerprinting. Mol. Ecol. 1: 79-87.

4. Largiader CR, Fries V, Bakker TCM (2001) Genetic analysis of sneaking and egg-thievery in a natural population of the three-spined stickleback (*Gasterosteus aculeatus* L.). Heredity 86: 459-468.

5. Rios-Cardenas O, Webster AS (2005) Paternity and paternal effort in the pumpkinseed sunfish. Behav. Ecol. 16: 914-921.

6. Neff BD, Clare EL (2008) Temporal variation in cuckoldry and paternity in two sunfish species (*Lepomis* spp.) with alternative reproductive tactics. Can. J. Zool. 86: 92-98.

7. Magee SE, Neff BD (2006) Temporal variation in decisions about parental care in bluegill, *Lepomis macrochirus.* Ethology 112: 1000-1007.

8. Fu P, Neff BD, Gross MR (2001) Tactic-specific success in sperm competition. Proc. R. Soc., Ser. B 268: 1105-1112.

9. Neff BD (2001) Genetic paternity analysis and breeding success in bluegill sunfish (*Lepomis macrochiros*). J. Hered 92: 111-119.

10. Neff BD (2003) Paternity and condition affect cannibalistic behavior in nest-tending bluegill sunfish. Behav. Ecol. Sociobiol. 54: 377-384.

11. Neff BD (2003) Decisions about parental care in response to perceived paternity. Nature 422: 716-719.

12. Neff BD, Gross MR (2001) Dynamic adjustment of parental care in response to perceived paternity. Proc. R. Soc., Ser. B 268: 1559-1565.

13. Green WW, Mirza RS, Pyle GG (2008) Kin recognition and cannibalistic behaviours by adult male fathead minnows (*Pimephales promelas*). Naturwissenschaften 95: 269-272.

14. Bessert ML, Brozek J, Orti G (2007) Impact of nest substrate limitations on patterns of illegitimacy in the fathead minnow, *Pimephales promelas* (Cypriniformes : Cyprinidae). J. Hered.98: 716-722.

15. Svensson O, Magnhagen C, Forsgren E, Kvarnemo C (1998) Parental behaviour in relation to the occurrence of sneaking in the common goby. Anim. Behav. 56: 175-179.

16. Svensson O, Kvarnemo C (2007) Parasitic spawning in sand gobies: an experimental assessment of nest-opening size, sneaker male cues, paternity, and filial cannibalism. Behav. Ecol. 18: 410-419.

17. Jones AG, Walker D, Kvarnemo C, Lindstrom K, Avise JC (2001) How cuckoldry can decrease the opportunity for sexual selection: data and theory from a genetic parentage analysis of the sand goby, *Pomatoschistus minutus*. PNAS 98: 9151-9156.

18. Ostlund-Nilsson S (2002) Does paternity or paternal investment determine the level of paternal care and does female choice explain egg stealing in the fifteen-spined stickleback? Behav. Ecol. 13: 188-192.

19. Jones AG, Ostlund-Nilsson S, Avise JC (1998) A microsatellite assessment of sneaked fertilizations and egg thievery in the fifteenspine stickleback. Evolution 52: 848-858.

20. Westneat DF (1995) Paternity and paternal behavior in the red-winged blackbird, *Agelaius phoeniceus*. Anim. Behav. 49: 21-35.

21. Gray EM (1996) Female control of offspring paternity in a western population of red-winged blackbirds (*Agelaius phoeniceus*). Behav. Ecol. Sociobiol. 38: 267-278.

22. Weatherhead PJ, Boag PT (1995) Pair and extra-pair mating success relative to male quality in red-winged blackbirds. Behav. Ecol. Sociobiol. 37: 81-91.

23. Westneat DF (1993) Temporal patterns of within-pair copulations, male mate-guarding, and extra-pair events in eastern red-winged blackbirds (*Agelaius phoeniceus*). Behaviour 124: 267-290.

24. Westneat DF, Gray EM (1998) Breeding synchrony and extrapair fertilizations in two populations of red-winged blackbirds. Behav. Ecol. 9: 456-464.

25. Westneat DF, Mays HL (2005) Tests of spatial and temporal factors influencing extra-pair paternity in red-winged blackbirds. Mol. Ecol. 14: 2155-2167.

26. Wagner RH (1992) Confidence of paternity and paternal effort in razorbills. Auk 109: 556-562.

27. Briskie JV, Montgomerie R, Poldmaa T, Boag PT (1998) Paternity and paternal care in the polygynandrous Smith's longspur. Behav. Ecol. Sociobiol. 43: 181-190.

28. Canestrari D, Marcos JM, Baglione V (2005) Effect of parentage and relatedness on the individual contribution to cooperative chick care in carrion crows *Corvus corone* *corone*. Behav. Ecol. Sociobiol. 57: 422-428.

29. Baglione V.

30. Baglione V, Marcos J, Canestrari D (2002) Cooperatively breeding groups of Carrion Crow (*Corvus corone corone*) in northern Spain. The Auk 119: 799.

31. Leech DI, Rowe LV, Hartley IR (2006) Experimental evidence for adjustment of parental investment in relation to brood sex ratio in the blue tit. Anim. Behav. 72: 1301-1307.

32. Charmantier A, Blondel J (2003) A contrast in extra-pair paternity levels on mainland and island populations of mediterranean blue tits. Ethology 109: 351-363.

33. Charmantier A, Perret P (2004) Manipulation of nest-box density affects extra-pair paternity in a population of blue tits (*Parus caeruleus*). Behav. Ecol. Sociobiol. 56: 360-365.

34. Delhey K, Johnsen A, Peters A, Andersson S, Kempenaers B (2003) Paternity analysis reveals opposing selection pressures on crown coloration in the blue tit (*Parus caeruleus*). Proc. R. Soc., Ser. B 270: 2057-2063.

35. Dreiss AN, Silva N, Richard M, Moyen F, Thery M, et al. (2008) Condition-dependent genetic benefits of extrapair fertilization in female blue tits *Cyanistes caeruleus*. J. Evol. Biol. 21: 1814-1822.

36. Foerster K, Delhey K, Johnsen A, Lifjeld JT, Kempenaers B (2003) Females increase offspring heterozygosity and fitness through extra-pair matings. Nature 425: 714-717.

37. Gullberg A, Tegelstrom H, Gelter HP (1992) DNA fingerprinting reveals multiple paternity in families of great and blue tits (*Parus major* and *P. caeruleus*). Hereditas 117: 103-108.

38. Kempenaers B, Verheyren GR, Dhondt AA (1997) Extrapair paternity in the blue tit (*Parus caeruleus*): female choice, male characteristics, and offspring quality. Behav. Ecol. 8: 481-492.

39. Krokene C, Rigstad K, Dale M, Lifjeld JT (1998) The function of extrapair paternity in blue tits and great tits: good genes or fertility insurance? Behav. Ecol. 9: 649-656.

40. Leech DI, Hartley IR, Stewart IRK, Griffith SC, Burke T (2001) No effect of parental quality or extrapair paternity on brood sex ratio in the blue tit (*Parus caeruleus*). Behav. Ecol. 12: 674-680.

41. Magrath MJL, Vedder O, van der Velde M, Komdeur J (2009) Maternal effects contribute to the superior performance of extra-pair offspring. Curr. Biol. 19: 792-797.

42. Vedder O, Magrath MJL, Harts AMF, Schut E, van der Velde M, et al. (2010) Reduced extrapair paternity in response to experimental stimulation of earlier incubation onset in blue tits. Behav. Ecol. 21: 9-15.

43. Williams DA, Hale AM (2008) Investment in nesting activities and patterns of extra- and within-group genetic paternity in a cooperatively breeding bird. Condor 110: 13-23.

44. Williams DA (2004) Female control of reproductive skew in cooperatively breeding brown jays (*Cyanocorax morio*). Behav. Ecol. Sociobiol. 55: 370-380.

45. Whittingham LA, Lifjeld JT (1995) High paternal investment in unrelated young - extra-pair paternity and male parental care in house martins. Behav. Ecol. Sociobiol. 37: 103-108.

46. Riley HT, Bryant DM, Carter RE, Parkin DT (1995) Extra-pair fertilizations and paternity defense in-house martins, *Delichon Urbica*. Anim. Behav. 49: 495-509.

47. Whittingham LA, Lifjeld JT (1995) Extra-pair fertilizations increase the opportunity for sexual selection in the monogamous house martin, *Delichon urbica*. J. Avian Biol. 26: 283-288.

48. Chuang-Dobbs HC, Webster MS, Holmes RT (2001) Paternity and parental care in the black-throated blue warbler, *Dendroica caerulescens*. Anim. Behav. 62: 83-92.

49. Holmes RT, Rodenhouse NL, Sillett TS (2005) Black-throated blue warbler (*Dendroica caerulescens*). The Birds of North America Online: Ithaca: Cornell Lab of Ornithology.

50. Yezerinac SM, Weatherhead PJ, Boag PT (1996) Cuckoldry and lack of parentage-dependent paternal care in yellow warblers: A cost-benefit approach. Anim. Behav. 52: 821-832.

51. Lowther PE, Celada C, Klein NK, Rimmer CC, Spector DA (1999) Yellow warbler (*Dendroica petachia*). The Birds of North America Online: Ithaca: Cornell Lab of Ornithology.

52. Yezerinac SM, Gibbs HL, Briskie JV, Whittam R, Montgomerie R (1999) Extrapair paternity in a far northern population of Yellow Warblers *Dendroica petechia*. J. Avian Biol. 30: 234-237.

53. Bouwman KM, Lessells CM, Komdeur J (2005) Male reed buntings do not adjust parental effort in relation to extrapair paternity. Behav. Ecol. 16: 499-506.

54. Bouwman KM, Burke T, Komdeur J (2006) How female reed buntings benefit from extra-pair mating behaviour: testing hypotheses through patterns of paternity in sequential broods. Mol. Ecol. 15: 2589-2600.

55. Dixon A, Ross D, Omalley SLC, Burke T (1994) Paternal investment inversely related to degree of extra-pair paternity in the reed bunting. Nature 371: 698-700.

56. Kleven O, Lifjeld JT (2005) No evidence for increased offspring heterozygosity from extrapair mating in the reed bunting (*Emberiza schoeniclus*). Behav. Ecol. 16: 561-565.

57. Suter SM, Keiser M, Feignoux R, Meyer DR (2007) Reed bunting females increase fitness through extra-pair mating with genetically dissimilar males. Proc. R. Soc., Ser. B 274: 2865-2871.

58. Suter SM, Bielanska J, Rothlin-Spillmann S, Strambini L, Meyer DR (2009) The cost of infidelity to female reed buntings. Behav. Ecol. 20: 601-608.

59. Sheldon BC, Rasanen K, Dias PC (1997) Certainty of paternity and paternal effort in the collared flycatcher. Behav. Ecol. 8: 421-428.

60. Krist M, Nadvornik P, Uvirova L, Bures S (2005) Paternity covaries with laying and hatching order in the collared flycatcher, *Ficedula albicollis*. Behav. Ecol. Sociobiol. 59: 6-11.

61. Rosivall B, Szollosi E, Hasselquist D, Torok J (2009) Effects of extrapair paternity and sex on nestling growth and condition in the collared flycatcher, *Ficedula albicollis*. Anim. Behav. 77: 611-617.

62. Sheldon BC, Ellegren H (1999) Sexual selection resulting from extrapair paternity in collared flycatchers. Anim. Behav. 57: 285-298.

63. Wilk T, Cichon M, Wolff K (2008) Lack of evidence for improved immune response of extra-pair nestlings in collared flycatcher *Ficedula albicollis*. J. Avian Biol. 39: 546-552.

64. Sheldon BC, Ellegren H (1998) Paternal effort related to experimentally manipulated paternity of male collared flycatchers. Proc. R. Soc., Ser. B 265: 1737-1742.

65. Lifjeld JT, Slagsvold T, Ellegren H (1998) Experimentally reduced paternity affects paternal effort and reproductive success in pied flycatchers. Anim. Behav. 55: 319-329.

66. Brun J, Winkel W, Epplen JT, Lubjuhn T (1996) Parentage analyses in the pied flycatcher Ficedula hypoleuca at the western boundary of its central European range. J. Ornithol. 137: 435-446.

67. Ellegren H, Lifjeld JT, Slagsvold T, Primmer CR (1995) Handicapped males and extrapair paternity in pied flycatchers: A study using microsatellite markers. Mol. Ecol. 4: 739-744.

68. Gelter HP, Tegelstrom H (1992) High-Frequency of Extra-pair paternity in swedish pied flycatchers revealed by allozyme electrophoresis and DNA fingerprinting. Behav. Ecol. Sociobiol. 31: 1-7.

69. Lehtonen TK, Lindstrom K (2009) Females decide whether size matters: plastic mate preferences tuned to the intensity of male-male competition. Behav. Ecol. 20: 195-199.

70. Lifjeld JT, Slagsvold T, Lampe HM (1991) Low-frequency of extra-pair paternity in pied flycatchers revealed by DNA fingerprinting. Behav. Ecol. Sociobiol. 29: 95-101.

71. Lubjuhn T, Winkel W, Epplen JT, Brun J (2000) Reproductive success of monogamous and polygynous pied flycatchers (*Ficedula hypoleuca*). Behav. Ecol. Sociobiol. 48: 12-17.

72. Moreno J, Martinez JG, Morales J, Lobato E, Merino S, et al. (2010) Paternity loss in relation to male age, territorial behaviour and stress in the pied flycatcher. Ethology 116: 76-84.

73. Ratti O, Hovi M, Lundberg A, Tegelstrom H, Alatalo RV (1995) Extra-Pair Paternity and Male Characteristics in the Pied Flycatcher. Behav. Ecol. Sociobiol. 37: 419-425.

74. Peterson KA, Thusius KJ, Whittingham LA, Dunn PO (2001) Allocation of male parental care in relation to paternity within and among broods of the common yellowthroat (*Geothlypis trichas*). Ethology 107: 573-586.

75. Abroe B, Garvin JC, Pedersen MC, Whittingham LA, Dunn PO (2007) Brood sex ratios are related to male size but not to attractiveness in common yellowthroats (*Geothlypis trichas*). Auk 124: 176-184.

76. Maguire S, Safran RJ (2010) Morphological and genetic predictors of parental care in the North American barn swallow, *Hirundo rustica erythrogaster*. J. Avian Biol. 41: 74-82.

77. Kleven O, Jacobsen F, Izadnegahdar R, Robertson RJ, Lifjeld JT (2006) No evidence of paternal genetic contribution to nestling cell-mediated immunity in the North American barn swallow. Anim. Behav. 71: 839-845.

78. Kojima W, Kitamura W, Kitajima S, Ito Y, Ueda K, et al. (2009) Female barn swallows gain indirect but not direct benefits through social mate choice. Ethology 115: 939-947.

79. Moller AP, Tegelstrom H (1997) Extra-pair paternity and tail ornamentation in the barn swallow, *Hirundo rustica*. Behav. Ecol. Sociobiol. 41: 353-360.

80. Primmer CR, Moller AP, Ellegren H (1995) Resolving genetic-relationships with microsatellite markers - a parentage testing system for the swallow, *Hirundo rustica*. Mol. Ecol. 4: 493-498.

81. Moller AP (1988) Paternity and paternal care in the swallow, *Hirundo rustica*. Anim. Behav. 36: 996-1005.

82. Dunn PO, Cockburn A (1996) Evolution of male parental care in a bird with almost complete cuckoldry. Evolution 50: 2542-2548.

83. Mulder RA, Dunn PO, Cockburn A, Lazenbycohen KA, Howell MJ (1994) Helpers liberate female fairy-wrens from constraints on extra-pair mate choice. Proc. R. Soc., Ser. B 255: 223-229.

84. Ewen JG, Armstrong DP (2000) Male provisioning is negatively correlated with attempted extrapair copulation frequency in the stitchbird (or hihi). Anim. Behav. 60: 429-433.

85. Ewen JG, Armstrong DP, Lambert DM (1999) Floater males gain reproductive success through extrapair fertilizations in the stitchbird. Anim. Behav. 58: 321-328.

86. Castro I, Mason KM, Armstrong DP, Lambert DM (2004) Effect of extra-pair paternity on effective population size in a reintroduced population of the endangered hihi, and potential for behavioural management. Conserv. Genet. 5: 381-393.

87. Lee JW, Kim HY, Hatchwell BJ (2010) Parental provisioning behaviour in a flock-living passerine, the Vinous-throated Parrotbill *Paradoxornis webbianus*. J. Ornithol. 151: 483-490.

88. Lee JW, Kim MS, Burke T, Hatchwell BJ (2009) Extrapair paternity in a flock-living passerine, the vinous-throated parrotbill *Paradoxornis webbianus*. J. Avian Biol. 40: 469-474.

89. Lubjuhn T, Curio E, Muth SC, Brun J, Epplen JT (1993) Influence of extra-pair paternity on parental care in great tits (*Parus major*). In: Pena SDJ, Chakraborty R, Epplen JT, Jeffreys AJ, editors. DNA Fingerprinting : State of the Science. Basel: Birkhauser Verlag. pp. 379-385.

90. Lubjuhn T, Strohbach S, Brun J, Gerken T, Epplen JT (1999) Extra-pair paternity in great tits (*Parus major*) - A long term study. Behaviour 136: 1157-1172.

91. Otter KA, Stewart IRK, McGregor PK, Terry AMR, Dabelsteen T, et al. (2001) Extra-pair paternity among Great Tits *Parus major* following manipulation of male signals. J. Avian Biol. 32: 338-344.

92. Verboven N, Mateman AC (1997) Low frequency of extra-pair fertilizations in the Great Tit *Parus major* revealed by DNA fingerprinting. J. Avian Biol. 28: 231-239.

93. Rytkonen S, Kvist L, Mikkonen R, Orell M (2007) Intensity of nest defence is not related to degree of paternity in the willow tit *Parus montanus*. J. Avian Biol. 38: 273-277.

94. Orell M, Rytkonen S, Launonen V, Welling P, Koivula K, et al. (1997) Low frequency extra-pair paternity in the Willow Tit *Parus montanus* as revealed by DNA fingerprinting. Ibis 139: 562-566.

95. Jamieson IG, Quinn JS, Rose PA, White BN (1994) Shared paternity among non-relatives is a result of an egalitarian mating system in a communally breeding bird, the pukeko. Proc. R. Soc., Ser. B 257: 271-277.

96. Wagner RH, Schug MD, Morton ES (1996) Confidence of paternity, actual paternity and parental effort by purple martins. Anim. Behav. 52: 123-132.

97. Wagner RH, Schug MD, Morton ES (1996) Condition dependent control of paternity by female purple martins: Implications for coloniality. Behav. Ecol. Sociobiol. 38: 379-389.

98. Morton ES, Forman L, Braun M (1990) Extrapair fertilizations and the evolution of colonial breeding in purple martins. Auk 107: 275-283.

99. Hartley IR, Davies NB, Hatchwell BJ, Desrochers A, Nebel D, et al. (1995) The polygynandrous mating system of the alpine accentor, *Prunella collaris* .2. multiple paternity and parental effort. Anim. Behav. 49: 789-803.

100. Heer L (1996) Cooperative breeding by Alpine accentors Prunella collaris: Polygynandry, territoriality and multiple paternity. Journal Fur Ornithologie 137: 35-51.

101. Davies NB, Hatchwell BJ, Robson T, Burke T (1992) Paternity and parental effort in dunnocks *Prunella modularis* - how good are male chick-feeding rules. Anim. Behav. 43: 729-745.

102. Burke T, Davies NB, Bruford MW, Hatchwell BJ (1989) Parental care and mating-behavior of polyandrous dunnocks *Prunella modularis* related to paternity by DNA fingerprinting. Nature 338: 249-251.

103. van Dijk RE, Meszaros LA, van der Velde M, Szekely T, Pogany A, et al. (2010) Nest desertion is not predicted by cuckoldry in the Eurasian penduline tit. Behav. Ecol. Sociobiol. 64: 1425-1435.

104. Schleicher B, Hoi H, Valera F, HoiLeitner M (1997) The importance of different paternity guards in the polygynandrous penduline tit (*Remiz pendulinus*). Behaviour 134: 941-959.

105. Whittingham LA, Dunn PO (1998) Male parental effort and paternity in a variable mating system. Anim. Behav. 55: 629-640.

106. Whittingham LA, Dunn PO, Magrath RD (1997) Relatedness, polyandry and extra-group paternity in the cooperatively-breeding white-browed scrubwren (*Sericornis frontalis*). Behav. Ecol. Sociobiol. 40: 261-270.

107. Dickinson JL, Weathers WW (1999) Replacement males in the western bluebird: opportunity for paternity, chick-feeding rules, and fitness consequences of male paternal care. Behav. Ecol. Sociobiol. 45: 201-209.

108. Dickinson JL, Akre JJ (1998) Extrapair paternity, inclusive fitness, and within-group benefits of helping in western bluebirds. Mol. Ecol. 7: 95-105.

109. Dickinson JL (2003) Male share of provisioning is not influenced by actual or apparent loss of paternity in western bluebirds. Behav. Ecol. 14: 360-366.

110. Kempenaers B, Lanctot RB, Robertson RJ (1998) Certainty of paternity and paternal investment in eastern bluebirds and tree swallows. Anim. Behav. 55: 845-860.

111. Stewart SLM, Westneat DF, Ritchison G (2010) Extra-pair paternity in eastern bluebirds: effects of manipulated density and natural patterns of breeding synchrony. Behav. Ecol. Sociobiol. 64: 463-473.

112. Meek SB, Robertson RJ, Boag PT (1994) Extrapair paternity and intraspecific brood parasitism in eastern bluebirds revealed by DNA-Fingerprinting. Auk 111: 739-744.

113. MacDougall-Shackleton EA, Robertson RJ (1998) Confidence of paternity and paternal care by eastern bluebirds. Behav. Ecol. 9: 201-205.

114. Garcia-Vigon E, Veiga JP, Cordero PJ (2009) Male feeding rate and extrapair paternity in the facultatively polygynous spotless starling. Anim. Behav. 78: 1335-1341.

115. Garcia-Vigon E, Cordero PJ, Veiga JP (2008) Exogenous testosterone in female spotless starlings reduces their rate of extrapair offspring. Anim. Behav. 76: 345-353.

116. Cordero PJ, Veiga JP, Moreno J, Parkin DT (2003) Extra-pair paternity in the facultatively polygynous spotless starling, *Sturnus unicolor*. Behav. Ecol. Sociobiol. 54: 1-6.

117. Eens M, Pinxten R (1995) Intersexual conflicts over copulations in the European starling - evidence for the female mate guarding hypothesis. Behav. Ecol. Sociobiol. 36: 71-81.

118. Loyau A, Moureau B, Richard M, Christe P, Heeb P, et al. (2005) Cross-amplification of polymorphic microsatellites reveals extra-pair paternity and brood parasitism in *Sturnus vulgaris*. Mol. Ecol. Notes 5: 135-139.

119. Pinxten R, Eens M, Verheyen RF (1993) Male and Female nest attendance during incubation in the facultatively polygynous european starling. Ardea 81: 125-133.

120. Smith HG, Sandell MI (1998) Intersexual competition in a polygynous mating system. Oikos 83: 484-495.

121. Osorio-Beristain H, Drummond H (2001) Male boobies expel eggs when paternity is in doubt. Behav. Ecol. 12: 16-21.

122. Whittingham LA, Dunn PO, Stapleton MK (2006) Repeatability of extra-pair mating in tree swallows. Mol. Ecol. 15: 841-849.

123. Stapleton MK, Kleven O, Lifjeld JT, Robertson RJ (2007) Female tree swallows (*Tachycineta bicolor*) increase offspring heterozygosity through extrapair mating. Behav. Ecol. Sociobiol. 61: 1725-1733.

124. O'Brien EL, Dawson RD (2007) Context-dependent genetic benefits of extra-pair mate choice in a socially monogamous passerine. Behav. Ecol. Sociobiol. 61: 775-782.

125. Conrad KF, Johnston PV, Crossman C, Kempenaers B, Robertson RJ, et al. (2001) High levels of extra-pair paternity in an isolated, low-density, island population of tree swallows (*Tachycineta bicolor*). Mol. Ecol. 10: 1301-1308.

126. Barber CA, Robertson RJ, Boag PT (1996) The high frequency of extra pair paternity in tree swallows is not an artifact of nestboxes. Behav. Ecol. Sociobiol. 38: 425-430.

127. Whittingham LA, Dunn PO, Robertson RJ (1993) Confidence of paternity and male parental care - an experimental study in tree swallows. Anim. Behav. 46: 139-147.

128. Wright J, Parker P, Lundy K (1999) Relatedness and chick-feeding effort in the cooperatively breeding Arabian babbler. Anim. Behav. 58: 779-785.

129. Lundy KJ, Parker PG, Zahavi A (1998) Reproduction by subordinates in cooperatively breeding Arabian babblers is uncommon but predictable. Behav. Ecol. Sociobiol. 43: 173-180.

130. Stutchbury BJ, Rhymer J, Morton E (1994) Extrapair paternity in hooded warblers. Behav. Ecol. 5: 384-392.

131. Scott MP, Williams SM (1993) Comparative reproductive success of communally breeding burying beetles as assessed by PCR with randomly amplified polymorphic DNA. PNAS 90: 2242-2245.

132. Garcia-Gonzalez F, Nunez Y, Ponz F, Roldan ERS, Gomendio M (2005) Paternity analysis in the golden egg bug using AFLPs: do the males preferentially accept their true genetic offspring? Ecol. Ent. 30: 444-455.

133. Tay WT, Miettinen M, Kaitala A (2003) Do male golden egg bugs carry eggs they have fertilized? A microsatellite analysis. Behav. Ecol. 14: 481-485.

134. Alvergne A, Faurie C, Raymond M (2009) Father-offspring resemblance predicts paternal investment in humans. Anim. Behav. - 78: - 69.

135. Anderson KG (2006) How well does paternity confidence match actual paternity? Evidence from worldwide nonpaternity rates. Curr. Anthropol. 47: 513-520.

136. Voracek M, Haubner T, Fisher ML (2008) Recent decline in nonpaternity rates: a cross-temporal meta-analysis. Psychol. Rep. 103: 799-811.

137. Anderson KG, Kaplan H, Lam D, Lancaster J (1999) Paternal care by genetic fathers and stepfathers II: reports by Xhosa high school students. Evolution and Human Behavior 20: 433-451.

138. Anderson KG, Kaplan H, Lancaster JB (2007) Confidence of paternity, divorce, and investment in children by Albuquerque men. Evol. Hum. Behav. 28: 1-10.

139. Marlowe F (1999) Male care and mating effort among Hadza foragers. Behav. Ecol. Sociobiol. 46: 57-64.

140. Paul A, Kuester J, Arnemann J (1996) The Sociobiol. of male-infant interactions in Barbary macaques, *Macaca sylvanus*. Anim. Behav. 51: 155-170.

141. Brauch K, Hodges K, Engelhardt A, Fuhrmann K, Shaw E, et al. (2008) Sex-specific reproductive behaviours and paternity in free-ranging Barbary macaques (Macaca sylvanus). Behav. Ecol. Sociobiol. 62: 1453-1466.

142. Kummerli R, Martin RD (2005) Male and female reproductive success in *Macaca sylvanus* in Gibraltar: No evidence for rank dependence. Int. J. Primatol. 26: 1229-1249.

143. Paul A, Kuester J, Arnemann J (1992) DNA fingerprinting reveals that infant care by male Barbary macaques (*Macaca sylvanus*) is not paternal investment. Folia Primatol. 58: 93-98.

144. Nguyen N, Van Horn R, Alberts S, Altmann J (2009) "Friendships" between new mothers and adult males: adaptive benefits and determinants in wild baboons (*Papio cynocephalus*). J. Avian Biol. 63: 1344.

145. Alberts SC, Buchan JC, Altmann J (2006) Sexual selection in wild baboons: from mating opportunities to paternity success. Anim. Behav. 72: 1177-1196.

146. Anderson CM (1992) Male investment under changing conditions among chacma baboons at Suikerbosrand. Am. J. Phys. Anthropol. 87: 479-496.

147. Huchard E, Alvergne A, Fejan D, Knapp LA, Cowlishaw G, et al. (2010) More than friends? Behavioural and genetic aspects of heterosexual associations in wild chacma baboons. Behav. Ecol. Sociobiol. 64: 769-781.

148. Moscovice LR, Di Fiore A, Crockford C, Kitchen DM, Wittig R, et al. (2010) Hedging their bets? Male and female chacma baboons form friendships based on likelihood of paternity. Anim. Behav. 79: 1007-1015.

149. While GM, Uller T, Wapstra E (2009) Family conflict and the evolution of sociality in reptiles. Behav. Ecol. 20: 245-250.
