# Supplementary material for: Why Do Cuckolded Males Provide Paternal Care?
Source: PLoS Biol. 2013 Mar 26;11(3):e1001520. doi: 10.1371/journal.pbio.1001520 (PMC3608547; doi:10.1371/journal.pbio.1001520)
Supplement: Table S2 — Data used for analysis of rCost. (DOCX) [file pbio.1001520.s006.docx]

**Table S2: Data used for analysis of r_Cost_**

| Species | Common name | n | r cost | Amount or Probability | Experiment or Observation | Care measure | Cost measure | Reference |
| --- | --- | --- | --- | --- | --- | --- | --- | --- |
| Abudefduf sexfasciatus | Scissortail sergeant | 70 | 0.17 | Probability | Experiment | Male Care vs No male care | Future reproductive success | [1] |
| Cyanistes caeruleus | Blue tit | 168 | 0.13 | Amount | Experiment | Brood size | Survival | [2] |
| Cyanistes caeruleus | Blue tit | 13 | 0.56 | Amount | Experiment | Brood size | Future reproductive success | [2] |
| Cyanistes caeruleus | Blue tit | 15 | 0.04 | Amount | Experiment | Brood size | Future reproductive success | [3] |
| Cyanistes caeruleus | Blue tit | 229 | 0.16 | Amount | Experiment | Brood size | Survival | [3] |
| Cyanistes caeruleus | Blue tit | 111 | 0.14 | Amount | Experiment | Brood size | Survival | [4] |
| Cyanistes caeruleus | Blue tit | 137 | 0.23 | Amount | Experiment | Brood size | Future reproductive success | [4] |
| Delichon urbica | House martin | 98 | -0.10 | Probability | Observation | Double vs single brood | Survival | [5] |
| Dendroica petechia | Yellow warbler | 28 | 0.39 | Amount | Observation | Feeding | Survival | [6] |
| Emberiza schoeniclus | Reed bunting | 25 | 0.10 | Amount | Observation | Feeding | Survival | [7] |
| Ficedula albicollis | Collared flycatcher | 110 | 0.35 | Amount | Experiment | Brood size | Future reproductive success | [8] |
| Ficedula albicollis | Collared flycatcher | 288 | 0.09 | Amount | Experiment | Brood size | Survival | [9] |
| Ficedula hypoleuca | Pied flycatcher | 111 | 0.17 | Amount | Experiment | Brood size | Survival | [10] |
| Ficedula hypoleuca | Pied flycatcher | 73 | 0.26 | Amount | Experiment | Brood size | Survival | [11] |
| Gasterosteus aculeatus | Three-spined stickleback | 20 | -0.26 | Amount | Experiment | Fanning | Mating opportunity | [12] |
| Gasterosteus aculeatus | Three-spined stickleback | 20 | 0.05 | Amount | Experiment1 | Fanning | Mating opportunity | [12] |
| Gasterosteus aculeatus | Three-spined stickleback | 20 | 0.38 | Amount | Experiment | Fanning | Mating opportunity | [12] |
| Geothlypis trichas | Yellowthroat | 24 | 0.36 | Amount | Observation | Feeding | Future reproductive success | [13] |
| Geothlypis trichas | Yellowthroat | 22 | 0.46 | Amount | Observation | Feeding | Future reproductive success | [13] |
| Homo sapiens | Human | 34 | 0.33 | Amount | Observation | Brood size | Mating opportunity | [14] |
| Lepomis macrochirus | Bluegill sunfish | 41 | 0.08 | Amount | Observation | Care duration | Future reproductive success | [15] |
| Lepomis macrochirus | Bluegill sunfish | 7 | 0.69 | Amount | Observation | Care duration | Future reproductive success | [15] |
| Nicrophorus vespilloides^1^ | Burying beetle | 40 | 0.43 | Amount | Experiment1 | Brood size | Future reproductive success | [16] |
| Notiomystis cincta | Stitchbird | 12 | 0.69 | Amount | Observation | Feeding | Mating opportunity | [17] |
| Parus major | Great tit | 57 | 0.03 | Probability | Experiment | Double vs single brood | Survival | [18] |
| Parus major | Great tit | 145 | 0.00 | Amount | Experiment | Brood size | Survival | [19] |
| Phyllomorpha laciniata | Golden egg bug | 11 | 0.75 | Probability | Experiment1 | Care vs No care | Survival | [20] |
| Phyllomorpha laciniata | Golden egg bug | 50 | 0.54 | Amount | Experiment | Brood size | Survival | [21] |
| Poecile montanus | Willow tit | 104 | 0.01 | Amount | Experiment | Brood size | Future reproductive success | [22] |
| Poecile montanus | Willow tit | 192 | 0.07 | Amount | Experiment | Brood size | Survival | [22] |
| Pomatoschistus microps | Common goby | 27 | 0.49 | Amount | Experiment | Fanning | Future reproductive success | [23] |
| Pomatoschistus minutus | Sand goby | 48 | -0.40 | Amount | Observation | Fanning | Mating opportunity | [24] |
| Pomatoschistus minutus | Sand goby | 48 | 0.47 | Amount | Observation | Care duration | Mating opportunity | [24] |
| Porphyrio porphyrio | Pukeko | 6 | 0.77 | Amount | Observation | Incubation | Mating opportunity | [25] |
| Remiz pendulinus | Penduline tit | 78 | 0.26 | Probability | Observation | Care vs No care | Mating opportunity | [26] |
| Sialia mexicana | Western bluebird | 13 | 0.04 | Probability | Experiment | Care vs No care | Future reproductive success | [27] |
| Sialia mexicana | Western bluebird | 13 | 0.06 | Probability | Experiment | Care vs No care | Survival | [27] |
| Sialia sialis | Eastern bluebird | 26 | 0.41 | Amount | Experiment | Brood size | Future reproductive success | [28] |
| Sialia sialis | Eastern bluebird | 56 | 0.23 | Amount | Experiment | Brood size | Future reproductive success | [29] |
| Sialia sialis | Eastern bluebird | 38 | 0.11 | Amount | Experiment | Brood size | Future reproductive success | [29] |
| Sialia sialis | Eastern bluebird | 80 | -0.10 | Amount | Experiment | Brood size | Survival | [29] |
| Sturnus vulgaris | Starling | 9 | -0.54 | Amount | Experiment | Feeding | Mating opportunity | [30] |
| Sturnus vulgaris | Starling | 29 | 0.42 | Amount | Experiment | Incubation | Mating opportunity | [30] |
| Tachycineta bicolor | Tree swallow | 22 | 0.10 | Amount | Experiment | Brood size | Survival | [31] |
| Tachycineta bicolor | Tree swallow | 53 | 0.01 | Amount | Experiment | Brood size | Survival | [32] |
| ^1^ No data available for *Nicrophorus tomentosus*), which was used to measure rAdjust, but different species have been used interchangeably for studies on paternal care. | | | | | | | | |

**References for Table S2**

1. Manica A (2004) Parental fish change their cannibalistic behaviour in response to the cost-to-benefit ratio of parental care. Anim Behav 67: 1015–1021.

2. Nur N (1988) The consequences of brood size for breeding blue tits. III. Measuring the cost of reproduction: survival, future fecundity, and differential dispersal. Evolution 42: 351–362.

3. Pettifor R (1993) Brood-Manipulation Experiments. II. A Cost of Reproduction in Blue Tits (Parus caeruleus)? J Anim Ecol 62: 145–159.

4. Parejo D, Danchin E (2006) Brood size manipulation affects frequency of second clutches in the blue tit. Behav Ecol Sociobiol 60: 184–194.

5. Bryant D (1979) Reproductive costs in the house martin (Delichon urbica). J Anim Ecol 48: 655–675.

6. Yezerinac SD (1996) Cuckoldry and lack of parentage-dependent paternal care in yellow warblers: A cost-benefit approach. Anim Behav 52: 821–832.

7. Bouwman K, Lessells C, Komdeur J (2005) Male reed buntings do not adjust parental effort in relation to extrapair paternity. Behav Ecol 16: 499–506. doi:10.1093/beheco/ari021.

8. Gustafsson L, Qvarnstrom A, Sheldon B (1995) Trade-Offs Between Life-History Traits and a Secondary Sexual Character in Male Collared Flycatchers. Nature 375: 311–313.

9. Gustafsson L, Sutherland W (1988) The costs of reproduction in the collared flycatcher Ficedulaalbicollis. Nature 335: 813–815.

10. Askenmo C (1979) Reproductive effort and return rate of male pied flycatchers. Am Nat 114: 748–753.

11. Siikamäki P, Hovi M (1997) Low male return rate due to clutch enlargements in the pied flycatcher (*Ficedula hypoleuca*). Ecoscience: 24–28.

12. de Fraipont M, Fitzgerald G, Guderley H (1994) Investment in present versus future broods: Parental care tactics in three-spined sticklebacks. Ecoscience 1: 15–20.

13. Mitchell D, Dunn P, Whittingham L, Freeman-Gallant C (2007) Attractive males provide less parental care in two populations of the common yellowthroat. Anim Behav 73: 165–170.

14. Winking J, Kaplan H, Gurven M, Rucas S (2007) Why do men marry and why do they stray? Proceedings of the Royal Society B-Biological Sciences 274: 1643–1649.

15. Cargnelli L, Neff B (2006) Condition-dependent nesting in bluegill sunfish Lepomis macrochirus. J Anim Ecol 75: 627–633.

16. Ward RJ, Cotter SC, Kilner RM (2009) Current brood size and residual reproductive value predict offspring desertion in the burying beetle Nicrophorus vespilloides. Behav Ecol 20: 1274–1281.

17. Low M, Joy MK, Makan T (2006) Using regression trees to predict patterns of male provisioning in the stitchbird (hihi). Anim Behav 71: 1057–1068.

18. Verhulst S (1998) Multiple breeding in the Great Tit, II. The costs of rearing a second clutch. Funct Ecol 12: 132–140.

19. Hõrak P (2003) When to pay the cost of reproduction? A brood size manipulation experiment in great tits (Parus major). Behav Ecol Sociobiol 54: 105–112.

20. Reguera P, Gomendio M (1999) Predation costs associated with parental care in the golden egg bug Phyllomorpha laciniata (Heteroptera: Coreidae). Behav Ecol 10: 541.

21. Kaitala A, Espadaler X, Lehtonen R (2000) Ant predation and the cost of egg carrying in the golden egg bug: experiments in the field. Oikos 89: 254–258.

22. Orell M, Rytkönen S, Koivula K, Ronkainen M, Rahiala M (1996) Brood size manipulations within the natural range did not reveal intragenerational cost of reproduction in the Willow Tit Parus montanus. Ibis 138: 630–637.

23. Jones J, Reynolds J (1999) Costs of egg ventilation for male common gobies breeding in conditions of low dissolved oxygen. Anim Behav 57: 181–188.

24. Pampoulie C, Lindstrom K, St Mary C (2004) Have your cake and eat it too: male sand gobies show more parental care in the presence of female partners. Behav Ecol 15: 199.

25. Craig J, Jamieson I (1985) The relationship between presumed gamete contribution and parental investment in a communally breeding bird. Behav Ecol Sociobiol 17: 207–211.

26. Szentirmai I, Székely T, Komdeur J (2007) Sexual conflict over care: antagonistic effects of clutch desertion on reproductive success of male and female penduline tits. J Evol Biol 20: 1739–1744.

27. Dickinson J, Weathers W (1999) Replacement males in the western bluebird: opportunity for paternity, chick-feeding rules, and fitness consequences of male parental care. Behav Ecol Sociobiol 45: 201–209.

28. Siefferman L, Hill G (2005) Male eastern bluebirds trade future ornamentation for current reproductive investment. Biol Lett 1: 208–211.

29. Siefferman L, Hill G (2008) Sex‐specific costs of reproduction in Eastern Bluebirds Sialia sialis. Ibis 150: 32–39.

30. Smith H (1995) Experimental demonstration of a trade-off between mate attraction and parental care. Proc R Soc Lond B 260: 45–51.

31. Wheelwright N, Leary J, Fitzgerald C (1991) The costs of reproduction in tree swallows (Tachycineta bicolor). Can J Zool 69: 2540–2547.

32. Murphy MT, Armbrecth B, Vlamis E, Pierce A (2000) Is reproduction by tree swallows cost free? Auk 117: 902.
