# Supplementary material for: Why Do Cuckolded Males Provide Paternal Care?
Source: PLoS Biol. 2013 Mar 26;11(3):e1001520. doi: 10.1371/journal.pbio.1001520 (PMC3608547; doi:10.1371/journal.pbio.1001520)
Supplement: Table S3 — Data used for analysis of rBenefit. (DOCX) [file pbio.1001520.s007.docx]

**Table S3: Data used for analysis of r_Benefit_**

| Species | Common name | n | r benefit | Amount or Probability | Experiment or Observation | Offspring measure | Paternal care measure | Reference |
| --- | --- | --- | --- | --- | --- | --- | --- | --- |
| Agelaius phoeniceus | Red winged blackbird | 47 | 0.37 | Amount | Observation | Condition | Feeding effort | [1] |
| Agelaius phoeniceus | Red winged blackbird | 47 | 0.34 | Amount | Observation | Survival | Feeding effort | [1] |
| Agelaius phoeniceus | Red winged blackbird | 81 | 0.36 | Probability | Observation | Survival | Male Care vs No male care | [1] |
| Agelaius phoeniceus | Red winged blackbird | 14 | 0.14 | Probability | Observation | Survival | Male Care vs No male care | [2] |
| Agelaius phoeniceus | Red winged blackbird | 120 | 0.27 | Probability | Observation | Survival | Male Care vs No male care | [3] |
| Agelaius phoeniceus | Red winged blackbird | 116 | 0.32 | Probability | Observation | Male condition | Male Care vs No male care | [3] |
| Agelaius phoeniceus | Red winged blackbird | 116 | 0.31 | Probability | Observation | Female condition | Male Care vs No male care | [3] |
| Agelaius phoeniceus | Red winged blackbird | 16 | 0.41 | Amount | Experiment | Male condition | Pairs vs Bigamy | [4] |
| Agelaius phoeniceus | Red winged blackbird | 16 | 0.19 | Amount | Experiment | Female condition | Pairs vs Bigamy | [4] |
| Agelaius phoeniceus | Red winged blackbird | 40 | 0.27 | Amount | Experiment | Survival | Pairs vs Bigamy | [4] |
| Cyanistes caeruleus | Blue tit | 112 | 0.42 | Amount | Observation | Survival | Feeding effort | [5] |
| Cyanistes caeruleus | Blue tit | 51 | 0.34 | Probability | Observation | Condition | Male Care vs No male care | [6] |
| Cyanistes caeruleus | Blue tit | 51 | 0.64 | Probability | Observation | Survival | Male Care vs No male care | [6] |
| Dendroica caerulescens | Black-throated blue warbler | 182 | 0.20 | Amount | Observation | Condition | Feeding effort | [7] |
| Dendroica petechia | Yellow warbler | 29 | 0.71 | Amount | Experiment | Condition | Feeding effort | [8] |
| Dendroica petechia | Yellow warbler | 29 | 0.23 | Amount | Experiment | Survival | Feeding effort | [8] |
| Emberiza schoeniclus | Reed bunting | 32 | 0.04 | Amount | Observation | Condition | Feeding effort | [9] |
| Emberiza schoeniclus | Reed bunting | 43 | 0.27 | Amount | Observation | Condition | Feeding effort | [10] |
| Emberiza schoeniclus | Reed bunting | 48 | 0.29 | Amount | Observation | Survival | Feeding effort | [10] |
| Emberiza schoeniclus | Reed bunting | 54 | 0.28 | Probability | Observation | Survival | Male Care vs No male care | [10] |
| Emberiza schoeniclus | Reed bunting | 47 | 0.46 | Probability | Observation | Condition | Male Care vs No male care | [10] |
| Ficedula albicollis | Collared flycatcher | 113 | 0.20 | Amount | Observation | Condition | Feeding effort | [11,11] |
| Ficedula albicollis | Collared flycatcher | 152 | 0.21 | Amount | Observation | Recruitment | Feeding effort | [11] |
| Ficedula hypoleuca | Pied flycatcher | 52 | 0.67 | Probability | Experiment | Condition | Male Care vs No male care | [12] |
| Ficedula hypoleuca | Pied flycatcher | 123 | 0.41 | Probability | Experiment | Survival | Male Care vs No male care | [12] |
| Ficedula hypoleuca | Pied flycatcher | 26 | 0.02 | Amount | Observation | Condition | Feeding effort | [13] |
| Ficedula hypoleuca | Pied flycatcher | 30 | 0.35 | Amount | Observation | Condition | Feeding effort | [14] |
| Ficedula hypoleuca | Pied flycatcher | 1704 | 0.39 | Probability | Observation | Recruitment | Male Care vs No male care | [15] |
| Ficedula hypoleuca | Pied flycatcher | 1924 | 0.77 | Probability | Observation | Survival | Male Care vs No male care | [15] |
| Ficedula hypoleuca | Pied flycatcher | 70 | 0.15 | Amount | Observation | Condition | Feeding effort | [16] |
| Ficedula hypoleuca | Pied flycatcher | 70 | 0.31 | Amount | Observation | Survival | Feeding effort | [16] |
| Gasterosteus aculeatus | Three-spined stickleback | 12 | 0.95 | Amount | Experiment | Survival | Time present | [17] |
| Hirundo rustica | Barn swallow | 35 | 0.48 | Amount | Experiment | Condition | Feeding effort | [18] |
| Hirundo rustica | Barn swallow | 35 | 0.52 | Amount | Experiment | Survival | Feeding effort | [18] |
| Homo sapiens | Human | 18967 | 0.005 | Probability | Observation | Survival | Male Care vs No male care | [19] |
| Homo sapiens | Human | 298 | 0.21 | Probability | Observation | Survival | Male Care vs No male care | [20] |
| Homo sapiens | Human | 3936 | 0.03 | Probability | Observation | Survival | Male Care vs No male care | [21] |
| Homo sapiens | Human | 674 | 0.06 | Probability | Observation | Survival | Male Care vs No male care | [22] |
| Homo sapiens | Human | 26500 | 0.06 | Probability | Observation | Survival | Male Care vs No male care | [23] |
| Homo sapiens | Human | 11452 | 0.01 | Probability | Observation | Survival | Male Care vs No male care | [24] |
| Homo sapiens | Human | 2130 | 0.04 | Probability | Observation | Survival | Male Care vs No male care | [25] |
| Homo sapiens | Human | 18201 | 0.00 | Probability | Observation | Survival | Male Care vs No male care | [26] |
| Homo sapiens | Human | 2621 | 0.04 | Probability | Observation | Survival | Male Care vs No male care | [27] |
| Homo sapiens | Human | 50064 | 0.04 | Probability | Observation | Survival | Male Care vs No male care | [28] |
| Homo sapiens | Human | 17361 | 0.03 | Probability | Observation | Survival | Male Care vs No male care | [29] |
| Homo sapiens | Human | 785 | 0.04 | Probability | Observation | Survival | Male Care vs No male care | [30] |
| Homo sapiens | Human | 3720 | -0.03 | Probability | Observation | Survival | Male Care vs No male care | [31] |
| Homo sapiens | Human | 1354 | 0.02 | Probability | Observation | Survival | Male Care vs No male care | [32] |
| Homo sapiens | Human | 25043 | 0.01 | Probability | Observation | Survival | Male Care vs No male care | [33] |
| Lepomis gibbosus | Pumpkinseed sunfish | 16 | 0.06 | Amount | Observation | Survival | Fanning | [34] |
| Lepomis gibbosus | Pumpkinseed sunfish | 18 | 0.72 | Amount | Observation | Survival | Defence | [34] |
| Lepomis macrochirus | Bluegill sunfish | 77 | 0.48 | Probability | Experiment | Survival | Male Care vs No male care | [35] |
| Lepomis macrochirus | Bluegill sunfish | 57 | 0.99 | Probability | Observation | Survival | Male Care vs No male care | [36,37] |
| Malurus cyaneus | Superb fairy-wren | 12 | 0.09 | Amount | Experiment | Survival | Feeding effort | [38] |
| Nicrophorus orbicollis^1^ | Burying beetle | 117 | 0.27 | Probability | Observation | Condition | Male Care vs No male care | [39] |
| Nicrophorus orbicollis^1^ | Burying beetle | 245 | 0.03 | Probability | Observation | Survival | Male Care vs No male care | [39] |
| Nicrophorus orbicollis^1^ | Burying beetle | 56 | 0.52 | Probability | Experiment | Condition | Male Care vs No male care | [40] |
| Nicrophorus orbicollis^1^ | Burying beetle | 25 | 0.16 | Probability | Experiment | Survival | Male Care vs No male care | [41] |
| Nicrophorus orbicollis^1^ | Burying beetle | 56 | 0.49 | Probability | Experiment | Survival | Male Care vs No male care | [40] |
| Nicrophorus defodiens^1^ | Burying beetle | 95 | 0.06 | Probability | Experiment | Condition | Male Care vs No male care | [40] |
| Nicrophorus defodiens^1^ | Burying beetle | 95 | 0.17 | Probability | Experiment | Survival | Male Care vs No male care | [40] |
| Nicrophorus defodiens^1^ | Burying beetle | 82 | 0.29 | Probability | Experiment | Survival | Male Care vs No male care | [40] |
| Nicrophorus defodiens^1^ | Burying beetle | 69 | 0.16 | Probability | Experiment | Survival | Male Care vs No male care | [42] |
| Nicrophorus defodiens^1^ | Burying beetle | 72 | 0.33 | Probability | Experiment | Survival | Male Care vs No male care | [40] |
| Nicrophorus defodiens^1^ | Burying beetle | 46 | 0.17 | Probability | Experiment | Survival | Male Care vs No male care | [43] |
| Nicrophorus vespilloides^1^ | Burying beetle | 46 | 0.12 | Probability | Experiment | Condition | Male Care vs No male care | [43] |
| Nicrophorus vespilloides^1^ | Burying beetle | 40 | 0.32 | Probability | Experiment | Condition | Male Care vs No male care | [44] |
| Nicrophorus vespilloides^1^ | Burying beetle | 40 | 0.11 | Probability | Experiment | Condition | Male Care vs No male care | [44] |
| Nicrophorus vespilloides^1^ | Burying beetle | 46 | 0.09 | Probability | Experiment | Survival | Male Care vs No male care | [43] |
| Nicrophorus vespilloides^1^ | Burying beetle | 40 | -0.23 | Probability | Experiment | Survival | Male Care vs No male care | [44] |
| Notiomystis cincta | Stitchbird | 64 | 0.17 | Amount | Observation | Survival | Feeding effort | [45] |
| Papio cynocephalus | Yellow baboon | 71 | 0.24 | Amount | Observation | Condition | Time present | [46] |
| Papio cynocephalus | Yellow baboon | 40 | 0.57 | Amount | Observation | Condition | Time present | [46] |
| Papio ursinus | Chacma baboon | 80 | 0.29 | Amount | Observation | Survival | Defence | [47] |
| Paradoxornis webbianus | Vinous-throated parrotbill | 26 | 0.54 | Amount | Observation | Condition | Feeding effort | [48] |
| Parus major | Great tit | 30 | -0.17 | Amount | Observation | Condition | Feeding effort | [49] |
| Parus major | Great tit | 35 | -0.12 | Amount | Observation | Condition | Feeding effort | [49] |
| Parus major | Great tit | 35 | 0.02 | Amount | Observation | Survival | Feeding effort | [49] |
| Phyllomorpha laciniata | Golden egg bug | 69 | 0.30 | Probability | Observation | Survival | Male Care vs No male care | [50] |
| Pimephales promelas | Fathead minnow | 150 | 0.89 | Amount | Observation | Survival | Defence | [51] |
| Pimephales promelas | Fathead minnow | 150 | 0.93 | Amount | Observation | Survival | Defence | [51] |
| Poecile montanus | Willow tit | 31 | 0.10 | Amount | Observation | Condition | Feeding effort | [52] |
| Poecile montanus | Willow tit | 31 | 0.18 | Amount | Observation | Recruitment | Feeding effort | [52] |
| Pomatoschistus microps | Common goby | 50 | 0.99 | Probability | Observation | Survival | Male Care vs No male care | [53] |
| Pomatoschistus minutus | Sand goby | 48 | 0.15 | Amount | Observation | Survival | Fanning | [54,55] |
| Pomatoschistus minutus | Sand goby | 48 | 0.23 | Amount | Observation | Survival | Fanning | [54,55] |
| Prunella collaris | Alpine accentor | 48 | 0.29 | Probability | Observation | Survival | Male Care vs No male care | [56] |
| Prunella collaris | Alpine accentor | 66 | 0.55 | Probability | Observation | Condition | Male Care vs No male care | [56] |
| Prunella modularis | Dunnock | 145 | 0.55 | Probability | Experiment | Survival | Male Care vs No male care | [57] |
| Sialia mexicana | Western bluebird | 10 | 0.29 | Probability | Experiment | Condition | Male Care vs No male care | [58] |
| Sialia mexicana | Western bluebird | 18 | 0.54 | Probability | Experiment | Survival | Male Care vs No male care | [58] |
| Sialia sialis | Eastern bluebird | 69 | 0.21 | Probability | Experiment | Condition | Male Care vs No male care | [59] |
| Sialia sialis | Eastern bluebird | 26 | -0.25 | Probability | Experiment | Survival | Male Care vs No male care | [59] |
| Sialia sialis | Eastern bluebird | 36 | 0.10 | Probability | Experiment | Condition | Male Care vs No male care | [60] |
| Sialia sialis | Eastern bluebird | 46 | 0.49 | Probability | Experiment | Survival | Male Care vs No male care | [60] |
| Spinachia spinachia | Fifteen-spined stickleback | 17 | 0.58 | Amount | Observation | Survival | Fanning | [61] |
| Sturnus unicolor | Spotless starling | 16 | 0.75 | Amount | Observation | Survival | Feeding effort | [62] |
| Sturnus unicolor | Spotless starling | 17 | 0.01 | Amount | Observation | Condition | Feeding effort | [63] |
| Sturnus unicolor | Spotless starling | 27 | 0.04 | Amount | Observation | Condition | Feeding effort | [63] |
| Sturnus unicolor | Spotless starling | 24 | 0.17 | Amount | Observation | Condition | Feeding effort | [63] |
| Sturnus unicolor | Spotless starling | 17 | 0.39 | Amount | Observation | Condition | Feeding effort | [63] |
| Sturnus vulgaris | Starling | 34 | 0.39 | Amount | Observation | Survival | Pairs vs Bigamy | [64] |
| Sturnus vulgaris | Starling | 42 | 0.21 | Amount | Observation | Survival | Feeding effort | [65] |
| Sturnus vulgaris | Starling | 13 | 0.31 | Amount | Experiment | Survival | Feeding effort | [66] |
| Sula nebouxii | Blue footed Booby | 42 | 0.36 | Amount | Experiment | Condition | Feeding effort | [67] |
| Tachycineta bicolor | Tree swallow | 83 | 0.51 | Amount | Observation | Condition | Feeding effort | [68] |
| Wilsonia citrina | Hooded warbler | 34 | 0.20 | Amount | Observation | Survival | Feeding effort | [69] |
| ^1^ No data available for *Nicrophorus tomentosus*), which was used to measure rAdjust, but different species have been used interchangeably for studies on paternal care. | | | | | | | | |

**References for Table S3**

1. Muldal A, Moffatt J, Robertson R (1986) Parental care of nestlings by male red-winged blackbirds. Behav Ecol Sociobiol 19: 105–114.

2. Whittingham L (1989) An experimental study of paternal behavior in red-winged blackbirds. Behav Ecol Sociobiol 25: 73–80.

3. Whittingham L, Robertson R (1994) Food availability, parental care and male mating success in red-winged blackbirds (Agelaius phoeniceus). J Anim Ecol 63: 139–150.

4. Pribil S (2000) Experimental evidence for the cost of polygyny in the red-winged blackbird Agelaius phoeniceus. Behaviour 137: 1153–1173.

5. Kempenaers B (1994) Polygyny in the blue tit: unbalanced sex ratio and female aggression restrict mate choice. Anim Behav 47: 943–957.

6. Björklund M (1996) The effect of male presence on nestling growth and fluctuating asymmetry in the blue tit. Condor 98: 172–175.

7. Stodola KW, Linder E, Buehler B, Franzreb K, Kim D, et al. (2010) Relative influence of male and female care in determining nestling mass in a migratory songbird. J Avian Biol 41: 1–8.

8. Lozano G, Lemon R (1996) Male plumage, parental care and reproductive success in yellow warblers, Dendroica petechia. Anim Behav 51: 265–272.

9. Bouwman K, Lessells C, Komdeur J (2005) Male reed buntings do not adjust parental effort in relation to extrapair paternity. Behav Ecol 16: 499–506.

10. Suter SM, Bielanska J, Rothlin-Spillmann S, Strambini L, Meyer DR (2009) The cost of infidelity to female reed buntings. Behav Ecol 20: 601–608.

11. Sheldon B (2002) Relating paternity to paternal care. Philos Trans R Soc Lond B Biol Sci 357: 341–350.

12. Alatalo R, Lundberg A, Ståhlbrandt K (1982) Why do pied flycatcher females mate with already-mated males? Anim Behav 30: 585–593.

13. Moreno J, Potti J, Merino S (1997) Parental energy expenditure and offspring size in the pied flycatcher Ficedula hypoleuca. Oikos 79: 559–567.

14. Lifjeld J, Slagsvold T, Ellegren H (1998) Experimentally reduced paternity affects paternal effort and reproductive success in pied flycatchers. Anim Behav 55: 319–329.

15. Huk T, Winkel W (2006) Polygyny and its fitness consequences for primary and secondary female pied flycatchers. Proc R Soc Lond B Biol Sci 273: 1681.

16. Moreno J, Morales J, Lobato E, Merino S, Tomás G, et al. (2006) More colourful eggs induce a higher relative paternal investment in the pied flycatcher Ficedula hypoleuca: a cross-fostering experiment. J Avian Biol 37: 555–560.

17. Bakker T, Mazzi D, Kraak S (2006) Broods of attractive three‐spined stickleback males require greater paternal care. J Fish Biol 69: 1164–1177.

18. Møller A (1988) Paternity and parental care in the swallow, Hirundo rustica. Anim Behav 36: 996–1005.

19. Andersson T, Hogberg U, Åkerman S (1996) Survival of orphans in 19th century Sweden—the importance of remarriages. Acta Paediatr 85: 981–985.

20. Hill K, Hurtado AM (1996) Ache life history: The ecology and demography of a foraging people. New York: Aldine de Gruyter.

21. Beekink E, Poppel F, Liefbroer A (1999) Surviving the loss of the parent in a nineteenth-century Dutch provincial town. J Soc Hist 32: 641–643.

22. Breschi M, Manfredini M (2002) Parental loss and kin networks: Demographic repercussions in a rural Italian village. In: Derosas R, Oris M, editors. When dad died: Individuals and families coping with distress in past societies. Bern: Peter Lang. pp. 369–387.

23. Derosas R (2002) Fatherless families in 19th century Venice. In: Derosas R, Oris M, editors. When dad died: Individuals and families coping with distress in past societies. Bern: Peter Lang. pp. 421–452.

24. Campbell C, Lee JZ (2002) When husbands and parents die: Widowhood and orphanhood in late Imperial Liaoning, 1789–1909. In: Derosas R, Oris M, editors. When dad died: Individuals and families coping with distress in past societies. Bern: Peter Lang. pp. 301–322.

25. Sear R, Steele F, McGregor I, Mace R (2002) The effects of kin on child mortality in rural Gambia. Demography 39: 43–63.

26. Sorenson Jamison C, Cornell L, Jamison P, Nakazato H (2002) Are all grandmothers equal? A review and a preliminary test of the “grandmother hypothesis” in Tokugawa Japan. Am J Phys Anthropol 119: 67–76.

27. Tsuya NO, Kurosu S (2002) The mortality effects of adult male death on women and children in agrarian households in early modern Japan: Evidence from two Northeastern villages, 1716–1870. In: Derosas R, Oris M, editors. When dad died: Individuals and families coping with distress in past societies. Bern: Peter Lang. pp. 261–299.

28. Beise J (2005) The helping grandmother and the helpful grandmother: The role of maternal and paternal grandmothers in child mortality in the 17th and 18th century population of French settlers in Quebec, Canada. In: Voland E, Chasiotis A, Schiefenhoevel W, editors. Grandmotherhood: The evolutionary significance of the second half of the female life. New runswick: Rutgers University Press. pp. 215–238.

29. Winking J, Gurven M (2011) The Impact of Parents and Self-Selection on Child Survival among the Tsimane of Bolivia. Curr Anthropol 52: 277-284.

30. Borgerhoff Mulder M (2007) Hamilton's rule and kin competition: the Kipsigis case. Evol Hum Behav 28: 299–312.

31. Gibson M (2008) Does Investment in the Sexes Differ When Fathers Are Absent? Hum Nat 19: 263–276.

32. Sear R (2008) Kin and child survival in Malawi: are matrilineal kin always beneficial in a matrilineal society? Hum Nat 19: 277–293.

33. Tymicki K (2009) The correlates of infant and childhood mortality. Demogr Res 20:559-594.

34. Rios-Cardenas O (2005) Paternity and paternal effort in the pumpkinseed sunfish. Behav Ecol 16: 914–921.

35. Bain MB, Helfrich LA (1983) Role of Male Parental Care in Survival of Larval Bluegills. Trans Am Fish Soc 112: 47–52.

36. Magee SE, Neff BD (2006) Temporal Variation in Decisions about Parental Care in Bluegill, Lepomis macrochirus. Ethology 112: 1000–1007.

37. Neff B (2003) Decisions about parental care in response to perceived paternity. Nature 422: 716–719.

38. Peters A, Cockburn A, Cunningham R (2002) Testosterone treatment suppresses paternal care in superb fairy-wrens, Malurus cyaneus , despite their concurrent investment in courtship. Behav Ecol Sociobiol 51: 538–547.

39. Trumbo S (1991) Reproductive benefits and the duration of paternal care in a biparental burying beetle, Necrophorus orbicollis. Behaviour.

40. Trumbo S (1994) Interspecific competition, brood parasitism, and the evolution of biparental cooperation in burying beetles. Oikos 69: 241–249.

41. Robertson I (1993) Nest intrusions, infanticide, and parental care in the burying beetle, Nicrophorus orbicollis (Coleoptera: Silphidae). J Zool 231: 583–593.

42. Scott M (1994) The benefit of paternal assistance in intra-and interspecific competition for the burying beetle, Nicrophorus defodiens. Ethol Ecol Evol 6: 537–543.

43. Müller J, Eggert A, Sakaluk S (1998) Carcass maintenance and biparental brood care in burying beetles: are males redundant? Ecol Entomol 23: 195–200.

44. Smiseth P, Dawson C, Varley E, Moore A (2005) How do caring parents respond to mate loss? Differential response by males and females. Anim Behav 69: 551–559.

45. Low M. Pers. Comm.

46. Charpentier M, Van Horn R, Altmann J, Alberts S (2008) Paternal effects on offspring fitness in a multimale primate society. Proc Natl Acad Sci USA 105: 1988.

47. Anderson C (1992) Male investment under changing conditions among chacma baboons at Suikerbosrand. Am J Phys Anthropol 87: 479–496.

48. Lee J-W, Kim H-Y, Hatchwell BJ (2010) Parental provisioning behaviour in a flock-living passerine, the Vinous-throated Parrotbill Paradoxornis webbianus. J Ornithol 151: 483–490.

49. Hinde CA Pers. Comm.

50. Reguera P, Gomendio M (2002) Flexible oviposition behavior in the golden egg bug (Phyllomorpha laciniata) and its implications for offspring survival. Behav Ecol 13: 70.

51. Divino JN, Tonn WM (2008) Importance of Nest and Paternal Characteristics for Hatching Success in Fathead Minnow. Copeia 2008: 920–930.

52. Rytkönen S, Orell M, Koivula K, Soppela M (1995) Correlation between two components of parental investment: nest defence intensity and nestling provisioning effort of willow tits. Oecologia 104: 386–393.

53. Svensson O Pers. Comm.

54. Pampoulie C, Lindstrom K, St Mary C (2004) Have your cake and eat it too: male sand gobies show more parental care in the presence of female partners. Behav Ecol 15: 199.

55. Lindström K Pers. Comm.

56. Nakamura M (1998) Multiple mating and cooperative breeding in polygynandrous alppine accentors. I. Competition among females. Anim Behav 55: 259–275.

57. Davies N, Hatchwell B (1992) The value of male parental care and its influence on reproductive allocation by male and female dunnocks. J Anim Ecol 61: 259–272.

58. Dickinson J, Weathers W (1999) Replacement males in the western bluebird: opportunity for paternity, chick-feeding rules, and fitness consequences of male parental care. Behav Ecol Sociobiol 45: 201–209.

59. Gowaty P (1983) Male parental care and apparent monogamy among eastern bluebirds (Sialia sialis). Am Nat 121: 149–157.

60. Meek S, Robertson R (1994) Effects of male removal on the behaviour and reproductive success of female eastern bluebirds Sialia sialis. Ibis 136: 305–312.

61. Östlund S, Ahnesjo I (1998) Female fifteen-spined sticklebacks prefer better fathers. Anim Behav 56: 1177–1183.

62. Moreno J, Veiga J, Cordero P, Mínguez E (1999) Effects of paternal care on reproductive success in the polygynous spotless starling Sturnus unicolor. Behav Ecol Sociobiol 47: 47–53.

63. Veiga J, Moreno J, Arenas M, Sanchez S (2002) Reproductive consequences for males of paternal vs territorial strategies in the polygynous spotless starling under variable ecological. Behaviour 139: 677–693.

64. Pinxten R, Eens M (1994) Male feeding of nestlings in the facultatively polygynous european starling - allocation patterns and effect on female reproductive success. Behaviour 129: 113–140.

65. Sandell M, Smith H, Bruun M (1996) Paternal care in the European starling, Sturnus vulgaris: nestling provisioning. Behav Ecol Sociobiol 39: 301–309.

66. De Ridder E, Pinxten R, Eens M (2000) Experimental evidence of a testosterone-induced shift from paternal to mating behaviour in a facultatively polygynous songbird. Behav Ecol Sociobiol 49: 24–30.

67. Velando A, Alonso-Alvarez C (2003) Differential body condition regulation by males and females in response to experimental manipulations of brood size and parental effort in the blue-footed booby. J Anim Ecol 72: 846–856.

68. Ardia D (2007) Site-and sex-level differences in adult feeding behaviour and its consequences to offspring quality in tree swallows (Tachycineta bicolor) following brood-size manipulation. Can J Zool 85: 847–854.

69. Buehler DM, Norris DR, Stutchbury BJM, Kopysh NC (2002) Food supply and parental feeding rates of hooded warblers in forest fragments. Wilson Bull 114: 122–127.
