# Supplementary material for: Why Do Cuckolded Males Provide Paternal Care?
Source: PLoS Biol. 2013 Mar 26;11(3):e1001520. doi: 10.1371/journal.pbio.1001520 (PMC3608547; doi:10.1371/journal.pbio.1001520)
Supplement: Table S4 — Meta-analysis of benefit: methodological effects. (DOCX) [file pbio.1001520.s008.docx]

**Table S4. Meta-analysis of benefits of male care for offspring**

**4(a) Modeling summary**

| **Table** | **Model** | **Fixed effects** |  | **Variation explained by random effects (%)** | | | |
| --- | --- | --- | --- | --- | --- | --- | --- |
|  |  |  | **DIC** | **Class** | **Family** | **Species** | **Study** |
| S4(b) | 1 | Intercept only | 0.10 | 65.02 | 1.62 | 15.58 | 10.66 |
| S4(c) | 2 | Amount vs probability of care | 2.67 | 66.99 | 1.91 | 15.31 | 7.71 |
| S4(d) | 3 | Observation vs experiment | -0.95 | 62.74 | 1.69 | 16.34 | 11.21 |
| S4(e) | 4 | Measure of offspring fitness | -15.39 | 64.10 | 1.60 | 14.16 | 12.93 |
| S4(f) | 5 | Amount vs probability + observation/experiment + offspring fitness | -12.29 | 67.05 | 1.85 | 15.31 | 8.97 |
| N_datapoints_=109, N_studies_=70, N_species_=34, N_families_=21, N_classes_=4. | | | |  | | | |

**S4(b) Model 1**

| **Fixed effects** | **Posterior mean (SD)** | **Posterior mode** | **Lower CI** | **Upper CI** | **pMCMC** |
| --- | --- | --- | --- | --- | --- |
| Intercept (mean effect size) | 0.51 (0.44) | 0.51 | -0.28 | 1.32 | 0.15 |
| **Random effects** | **Posterior mean (SD)** | **Posterior mode** | **Lower CI** | **Upper CI** |  |
| Class | 0.72 (3.50) | 0.33 | 0.002 | 2.19 |  |
| Family | 0.007 (0.01) | 0.0009 | 0.0001 | 0.03 |  |
| Species | 0.07 (0.05) | 0.002 | 0.0003 | 0.17 |  |
| Study | 0.05 (0.03) | 0.002 | 0.0002 | 0.11 |  |
| Residual variance | 0.03 (0.01) | 0.03 | 0.01 | 0.06 |  |

**S4(c) Model 2**

| **Fixed effects** | **Posterior mean (SD)** | **Posterior mode** | **Lower CI** | **Upper CI** | **pMCMC** |
| --- | --- | --- | --- | --- | --- |
| Care: amount | 0.40 (0.46) | 0.40 | -0.49 | 1.25 | 0.24 |
| Care: probability | 0.61 (0.46) | 0.56 | -0.29 | 1.44 | 0.10 |
| probability - amount | 0.21 (0.10) | 0.22 | 0.02 | 0.40 | **0.03** |
| **Random effects** | **Posterior mean (SD)** | **Posterior mode** | **Lower CI** | **Upper CI** |  |
| Class | 0.83 (2.24) | 0.28 | 0.009 | 2.50 |  |
| Family | 0.009 (0.01) | 0.001 | 0.0002 | 0.03 |  |
| Species | 0.07 (0.05) | 0.002 | 0.0003 | 0.16 |  |
| Study | 0.04 (0.03) | 0.002 | 0.0002 | 0.09 |  |
| Residual variance | 0.04 (0.01) | 0.03 | 0.01 | 0.06 |  |

**S4(d) Model 3**

| **Fixed effects** | **Posterior mean (SD)** | **Posterior mode** | **Lower CI** | **Upper CI** | **pMCMC** |
| --- | --- | --- | --- | --- | --- |
| Data: experimental | 0.51 (0.43) | 0.46 | -0.32 | 1.24 | 0.13 |
| Data: observational | 0.48 (0.44) | 0.54 | -0.32 | 1.27 | 0.16 |
| Observational - experimental | -0.03 (0.11) | -0.03 | -0.26 | 0.19 | 0.77 |
| **Random effects** | **Posterior mean (SD)** | **Posterior mode** | **Lower CI** | **Upper CI** |  |
| Class | 0.76 (3.43) | 0.25 | 0.0007 | 2.21 |  |
| Family | 0.007 (0.01) | 0.001 | 0.0002 | 0.03 |  |
| Species | 0.07 (0.06) | 0.002 | 0.0003 | 0.18 |  |
| Study | 0.05 (0.03) | 0.001 | 0.0003 | 0.11 |  |
| Residual variance | 0.03 (0.01) | 0.03 | 0.01 | 0.06 |  |

S**4(e) Model 4**

| **Fixed effects** | **Posterior mean (SD)** | **Posterior mode** | **Lower CI** | **Upper CI** | **pMCMC** |
| --- | --- | --- | --- | --- | --- |
| Offspring measure: condition | 0.51 (0.42) | 0.50 | -0.25 | 1.34 | 0.13 |
| Offspring measure: recruitment | 0.33 (0.45) | 0.36 | -0.56 | 1.12 | 0.36 |
| Offspring measure: survival | 0.52 (0.42) | 0.55 | -0.33 | 1.27 | 0.13 |
| recruitment - condition | -0.19 (0.16) | -0.27 | -0.51 | 0.14 | 0.24 |
| survival - condition | -0.002 (0.06) | -0.02 | -0.12 | 0.13 | 0.97 |
| survival - recruitment | 0.19 (0.17) | 0.22 | -0.14 | 0.51 | 0.26 |
| **Random effects** | **Posterior mean (SD)** | **Posterior mode** | **Lower CI** | **Upper CI** |  |
| Class | 0.70 (2.87) | 0.25 | 0.001 | 2.10 |  |
| Family | 0.007 (0.01) | 0.001 | 0.0002 | 0.02 |  |
| Species | 0.06 (0.05) | 0.002 | 0.0003 | 0.16 |  |
| Study | 0.06 (0.03) | 0.05 | 0.0004 | 0.11 |  |
| Residual variance | 0.03 (0.01) | 0.02 | 0.009 | 0.06 |  |

**S4(f) Model 5**

| **Fixed effects** | **Posterior mean (SD)** | **Posterior mode** | **Lower CI** | **Upper CI** | **pMCMC** |
| --- | --- | --- | --- | --- | --- |
| Care: amount | 0.42 (0.50) | 0.39 | -0.45 | 1.31 | 0.22 |
| Care: probability | 0.65 (0.49) | 0.65 | -0.26 | 1.50 | 0.10 |
| probability - amount | 0.22 (0.10) | 0.24 | 0.03 | 0.43 | **0.03** |
| Data: experimental | 0.44 (0.47) | 0.45 | -0.50 | 1.30 | 0.22 |
| Data: observational | 0.37 (0.48) | 0.30 | -0.56 | 1.25 | 0.29 |
| Observational - experimental | -0.07 (0.11) | -0.06 | -0.27 | 0.17 | 0.52 |
| Offspring measure: condition | 0.43 (0.46) | 0.47 | -0.41 | 1.26 | 0.21 |
| Offspring measure: recruitment | 0.23 (0.48) | 0.21 | -0.69 | 1.11 | 0.52 |
| Offspring measure: survival | 0.42 (0.46) | 0.41 | -0.38 | 1.30 | 0.22 |
| recruitment - condition | -0.21 (0.16) | -0.27 | -0.52 | 0.10 | 0.21 |
| survival - condition | -0.01 (0.06) | -0.02 | -0.13 | 0.11 | 0.83 |
| survival - recruitment | 0.20 (0.16) | 0.25 | -0.11 | 0.53 | 0.21 |
| **Random effects** | **Posterior mean (SD)** | **Posterior mode** | **Lower CI** | **Upper CI** |  |
| Class | 0.76 (2.37) | 0.22 | 0.008 | 2.33 |  |
| Family | 0.009 (0.01) | 0.001 | 0.0002 | 0.03 |  |
| Species | 0.07 (0.05) | 0.002 | 0.0002 | 0.17 |  |
| Study | 0.04 (0.03) | 0.002 | 0.0003 | 0.10 |  |
| Residual variance | 0.03 (0.01) | 0.02 | 0.008 | 0.06 |  |
