# Supplementary material for: Why Do Cuckolded Males Provide Paternal Care?
Source: PLoS Biol. 2013 Mar 26;11(3):e1001520. doi: 10.1371/journal.pbio.1001520 (PMC3608547; doi:10.1371/journal.pbio.1001520)
Supplement: Table S5 — Meta-analysis of cost: methodological effects. (DOCX) [file pbio.1001520.s009.docx]

**Table S5. Meta-analysis of the costs to future reproductive success of male care**

**S5(a) Modeling summary**

| **Table** | **Model** | **Fixed effects** |  | **Variation explained by random effects (%)** | | | |
| --- | --- | --- | --- | --- | --- | --- | --- |
|  |  |  | **DIC** | **Class** | **Family** | **Species** | **Study** |
| 5(b) | 1 | Intercept only | -56.82 | 42.44 | 15.36 | 9.33 | 7.90 |
| 5(c) | 2 | Amount vs probability of care | -52.46 | 40.69 | 15.08 | 9.43 | 8.16 |
| 5(d) | 3 | Observation vs experiment | -53.14 | 42.46 | 15.06 | 9.61 | 7.62 |
| 5(e) | 4 | Measure of Cost | -71.88 | 49.46 | 15.27 | 8.99 | 6.47 |
| 5(f) | 5 | ZrBenefit | -64.03 | 50.76 | 15.06 | 7.55 | 6.46 |
| 5(g) | 6 | Proportion of male care | -56.07 | 49.76 | 13.71 | 8.16 | 6.73 |
| 5(h) | 7 | Amount vs probability + observation vs experiment + cost + ZrBenefit | -72.21 | 58.84 | 13.06 | 7.16 | 5.11 |
| N_datapoints_=45, N_studies_=31, N_species_=24, N_families_=17, N_classes_=4. | | | |  | | | |

**S5(b) Model 1**

| **Fixed effects** | **Posterior mean (SD)** | **Posterior mode** | **Lower CI** | **Upper CI** | **pMCMC** |
| --- | --- | --- | --- | --- | --- |
| Intercept (mean effect size) | 0.27 (0.14) | 0.22 | 0.02 | 0.55 | **0.04** |
| **Random effects** | **Posterior mean (SD)** | **Posterior mode** | **Lower CI** | **Upper CI** |  |
| Class | 0.06 (0.20) | 0.01 | 0.0002 | 0.23 |  |
| Family | 0.01 (0.01) | 0.001 | 0.0002 | 0.03 |  |
| Species | 0.01 (0.01) | 0.001 | 0.0002 | 0.02 |  |
| Study | 0.004 (0.005) | 0.001 | 0.0002 | 0.01 |  |
| Residual variance | 0.01 (0.01) | 0.004 | 0.0002 | 0.04 |  |

**S5(c) Model 2**

| **Fixed effects** | **Posterior mean (SD)** | **Posterior mode** | **Lower CI** | **Upper CI** | **pMCMC** |
| --- | --- | --- | --- | --- | --- |
| Care: amount | 0.28 (0.16) | 0.25 | 0.05 | 0.60 | **0.03** |
| Care: probability | 0.23 (0.18) | 0.22 | -0.07 | 0.55 | 0.11 |
| probability - amount | -0.06 (0.10) | -0.05 | -0.26 | 0.13 | 0.57 |
| **Random effects** | **Posterior mean (SD)** | **Posterior mode** | **Lower CI** | **Upper CI** |  |
| Class | 0.06 (0.39) | 0.001 | 0.0002 | 0.22 |  |
| Family | 0.01 (0.01) | 0.001 | 0.0001 | 0.03 |  |
| Species | 0.01 (0.01) | 0.001 | 0.0001 | 0.02 |  |
| Study | 0.004 (0.005) | 0.001 | 0.0002 | 0.01 |  |
| Residual variance | 0.02 (0.01) | 0.01 | 0.0002 | 0.04 |  |

**S5(d) Model 3**

| **Fixed effects** | **Posterior mean (SD)** | **Posterior mode** | **Lower CI** | **Upper CI** | **pMCMC** |
| --- | --- | --- | --- | --- | --- |
| Data: experimental | 0.30 (0.17) | 0.29 | 0.001 | 0.63 | **0.04** |
| Data: observational | 0.26 (0.16) | 0.24 | -0.01 | 0.59 | 0.06 |
| Observational - experimental | -0.04 (0.10) | -0.03 | -0.24 | 0.15 | 0.70 |
| **Random effects** | **Posterior mean (SD)** | **Posterior mode** | **Lower CI** | **Upper CI** |  |
| Class | 0.08 (0.67) | 0.002 | 0.0002 | 0.24 |  |
| Family | 0.01 (0.01) | 0.001 | 0.0002 | 0.03 |  |
| Species | 0.01 (0.01) | 0.001 | 0.0002 | 0.02 |  |
| Study | 0.004 (0.005) | 0.001 | 0.0002 | 0.01 |  |
| Residual variance | 0.02 (0.01) | 0.01 | 0.0003 | 0.04 |  |

S**5(e) Model 4**

| **Fixed effects** | **Posterior mean (SD)** | **Posterior mode** | **Lower CI** | **Upper CI** | **pMCMC** |
| --- | --- | --- | --- | --- | --- |
| Cost measure: reproductive success | 0.34 (0.17) | 0.29 | 0.06 | 0.68 | **0.03** |
| Cost measure: mating opportunity | 0.29 (0.18) | 0.27 | -0.02 | 0.63 | **0.05** |
| Cost measure: survival | 0.19 (0.17) | 0.20 | -0.11 | 0.50 | 0.17 |
| mating opportunity - reproductive success | -0.05 (0.12) | -0.05 | -0.28 | 0.18 | 0.66 |
| survival - reproductive success | -0.15 (0.07) | -0.14 | -0.29 | -0.01 | **0.04** |
| survival - mating opportunity | -0.10 (0.12) | -0.09 | -0.32 | 0.62 | 0.41 |
| **Random effects** | **Posterior mean (SD)** | **Posterior mode** | **Lower CI** | **Upper CI** |  |
| Class | 0.11 (1.44) | 0.002 | 0.0002 | 0.30 |  |
| Family | 0.01 (0.01) | 0.001 | 0.0002 | 0.03 |  |
| Species | 0.01 (0.01) | 0.001 | 0.0002 | 0.02 |  |
| Study | 0.004 (0.004) | 0.001 | 0.0002 | 0.01 |  |
| Residual variance | 0.01 (0.01) | 0.001 | 0.0002 | 0.03 |  |

**S5(f) Model 5**

| **Fixed effects** | **Posterior mean (SD)** | **Posterior mode** | **Lower CI** | **Upper CI** | **pMCMC** |
| --- | --- | --- | --- | --- | --- |
| ZrBenefit | 0.05 (0.05) | 0.06 | -0.04 | 0.15 | 0.29 |
| **Random effects** | **Posterior mean (SD)** | **Posterior mode** | **Lower CI** | **Upper CI** |  |
| Class | 0.10 (1.03) | 0.003 | 0.0001 | 0.33 |  |
| Family | 0.01 (0.01) | 0.001 | 0.0002 | 0.04 |  |
| Species | 0.005 (0.006) | 0.001 | 0.0002 | 0.02 |  |
| Study | 0.004 (0.004) | 0.001 | 0.0002 | 0.01 |  |
| Residual variance | 0.01 (0.01) | 0.005 | 0.0002 | 0.03 |  |

**S5(g) Model 6**

| **Fixed effects** | **Posterior mean (SD)** | **Posterior mode** | **Lower CI** | **Upper CI** | **pMCMC** |
| --- | --- | --- | --- | --- | --- |
| Proportion of male care | -0.02 (0.10) | -0.006 | -0.22 | 0.20 | 0.79 |
| **Random effects** | **Posterior mean (SD)** | **Posterior mode** | **Lower CI** | **Upper CI** |  |
| Class | 0.12 (0.61) | -0.002 | 0.0002 | 0.40 |  |
| Family | 0.01 (0.01) | 0.001 | 0.0002 | 0.03 |  |
| Species | 0.005 (0.007) | 0.001 | 0.0002 | 0.02 |  |
| Study | 0.004 (0.005) | 0.001 | 0.0002 | 0.01 |  |
| Residual variance | 0.01 (0.01) | 0.007 | 0.0003 | 0.04 |  |

**S5(h) Model 7**

| **Fixed effects** | **Posterior mean (SD)** | **Posterior mode** | **Lower CI** | **Upper CI** | **pMCMC** |
| --- | --- | --- | --- | --- | --- |
| Care: amount | 0.40 (0.22) | 0.41 | 0.007 | 0.84 | **0.05** |
| Care: probability | 0.36 (0.23) | 0.33 | -0.08 | 0.81 | 0.08 |
| probability - amount | -0.05 (0.10) | -0.05 | -0.25 | 0.16 | 0.63 |
| Data: experimental | 0.40 (0.24) | 0.41 | -0.01 | 0.80 | **0.05** |
| Data: observational | 0.33 (0.22) | 0.33 | -0.04 | 0.74 | 0.07 |
| Observational - experimental | -0.07 (0.11) | -0.08 | -0.29 | 0.14 | 0.51 |
| Cost measure: reproductive success | 0.40 (0.23) | 0.38 | 0.01 | 0.85 | **0.05** |
| Cost measure: mating opportunity | 0.35 (0.23) | 0.38 | -0.04 | 0.79 | 0.07 |
| Cost measure: survival | 0.25 (0.23) | 0.19 | -0.15 | 0.69 | 0.17 |
| mating opportunity - reproductive success | -0.05 (0.13) | -0.06 | -0.30 | 0.19 | 0.67 |
| survival - reproductive success | -0.15 (0.07) | -0.14 | -0.28 | -0.01 | **0.05** |
| survival - mating opportunity | -0.09 (0.13) | -0.06 | -0.35 | 0.16 | 0.48 |
| ZrBenefit | 0.06 (0.05) | 0.06 | -0.04 | 0.17 | 0.24 |
| **Random effects** | **Posterior mean (SD)** | **Posterior mode** | **Lower CI** | **Upper CI** |  |
| Class | 0.15 (1.05) | 0.0003 | 0.0003 | 0.49 |  |
| Family | 0.01 (0.01) | 0.0002 | 0.0002 | 0.04 |  |
| Species | 0.01 (0.01) | 0.0002 | 0.0002 | 0.02 |  |
| Study | 0.004 (0.004) | 0.0002 | 0.0002 | 0.01 |  |
| Residual variance | 0.01 (0.01) | 0.0002 | 0.0002 | 0.03 |  |
