# Supplementary material for: Why Do Cuckolded Males Provide Paternal Care?
Source: PLoS Biol. 2013 Mar 26;11(3):e1001520. doi: 10.1371/journal.pbio.1001520 (PMC3608547; doi:10.1371/journal.pbio.1001520)
Supplement: Table S6 — Meta-analysis of adjustment: methodological effects. (DOCX) [file pbio.1001520.s010.docx]

**Table S6. Meta-analysis of the adjustment of male care: methodological effects**

**S6(a) Modeling summary**

| **Table** | **Model** | **Fixed effects** |  | **Variation explained by random effects (%)** | | | |
| --- | --- | --- | --- | --- | --- | --- | --- |
|  |  |  | **DIC** | **Class** | **Family** | **Species** | **Study** |
| S6(b) | 1 | Intercept only | -571.69 | 34.78 | 17.99 | 9.17 | 36.15 |
| S6(c) | 2 | Amount vs probability of care | -570.44 | 28.76 | 19.76 | 9.98 | 39.31 |
| S6(d) | 3 | Observation vs experiment | -572.51 | 39.44 | 17.42 | 9.14 | 32.21 |
| S6(e) | 4 | Access to female (yes vs no)? | -571.83 | 40.90 | 16.06 | 9.23 | 31.96 |
| S6(f) | 5 | Competitors present (yes vs no)? | -571.03 | 34.50 | 17.96 | 8.97 | 36.64 |
| S6(g) | 6 | Paternity measured using genetic markers (yes vs no)? | -573.87 | 39.52 | 17.81 | 9.25 | 31.64 |
| **S6(h)** | **7** | **Adjustment tested within vs across males** | **-596.31** | **35.31** | **18.11** | **10.13** | **34.98** |
| S6(i) | 8 | Amount vs probability + observation/experiment + female access + competitor presence + genetic data +within vs across | -588.75 | 35.75 | 17.13 | 11.71 | 33.68 |
| N_datapoints_=192, N_studies_=62, N_species_=48, N_families_=29, N_classes_=5. | | | |  | | | |

**S6(b) Model 1**

| **Fixed effects** | **Posterior mean (SD)** | **Posterior mode** | **Lower CI** | **Upper CI** | **pMCMC** |
| --- | --- | --- | --- | --- | --- |
| Intercept (mean effect size) | 0.35 (0.15) | 0.29 | 0.10 | 0.68 | **0.02** |
| **Random effects** | **Posterior mean (SD)** | **Posterior mode** | **Lower CI** | **Upper CI** |  |
| Class | 0.08 (0.27) | 0.03 | 0.0002 | 0.28 |  |
| Family | 0.02 (0.02) | 0.001 | 0.0002 | 0.06 |  |
| Species | 0.01 (0.01) | 0.001 | 0.0002 | 0.034 |  |
| Study | 0.04 (0.02) | 0.03 | 0.009 | 0.07 |  |
| Residual variance | 0.002 (0.002) | 0.0008 | 0.0002 | 0.005 |  |

**S6(c) Model 2**

| **Fixed effects** | **Posterior mean (SD)** | **Posterior mode** | **Lower CI** | **Upper CI** | **pMCMC** |
| --- | --- | --- | --- | --- | --- |
| Care: amount | 0.29 (0.13) | 0.25 | 0.07 | 0.58 | **0.02** |
| Care: probability | 0.43 (0.14) | 0.37 | 0.18 | 0.72 | **0.008** |
| probability - amount | 0.14 (0.08) | 0.13 | -0.02 | 0.29 | 0.09 |
| **Random effects** | **Posterior mean (SD)** | **Posterior mode** | **Lower CI** | **Upper CI** |  |
| Class | 0.06 (0.21) | 0.02 | 0.0002 | 0.21 |  |
| Family | 0.02 (0.02) | 0.001 | 0.0002 | 0.05 |  |
| Species | 0.01 (0.01) | 0.001 | 0.0002 | 0.03 |  |
| Study | 0.04 (0.02) | 0.03 | 0.01 | 0.07 |  |
| Residual variance | 0.002 (0.002) | 0.0008 | 0.0002 | 0.005 |  |

**S6(d) Model 3**

| **Fixed effects** | **Posterior mean (SD)** | **Posterior mode** | **Lower CI** | **Upper CI** | **pMCMC** |
| --- | --- | --- | --- | --- | --- |
| Data: observational | 0.34 (0.16) | 0.28 | 0.06 | 0.70 | **0.03** |
| Data: experimental | 0.41 (0.18) | 0.34 | 0.10 | 0.79 | **0.02** |
| experimental - observational | 0.07 (0.09) | 0.10 | -0.09 | 0.25 | 0.38 |
| **Random effects** | **Posterior mean (SD)** | **Posterior mode** | **Lower CI** | **Upper CI** |  |
| Class | 0.10 (0.22) | 0.01 | 0.0002 | 0.35 |  |
| Family | 0.02 (0.02) | 0.001 | 0.0003 | 0.06 |  |
| Species | 0.01 (0.01) | 0.001 | 0.0002 | 0.04 |  |
| Study | 0.04 (0.02) | 0.03 | 0.008 | 0.07 |  |
| Residual variance | 0.002 (0.002) | 0.0008 | 0.0002 | 0.005 |  |

**S6(e) Model 4**

| **Fixed effects** | **Posterior mean (SD)** | **Posterior mode** | **Lower CI** | **Upper CI** | **pMCMC** |
| --- | --- | --- | --- | --- | --- |
| Female access: no | 0.34 (0.16) | 0.33 | 0.07 | 0.68 | **0.02** |
| Female access: yes | 0.46 (0.18) | 0.39 | 0.16 | 0.82 | **0.01** |
| yes - no | 0.12 (0.07) | 0.09 | -0.02 | 0.26 | 0.10 |
| **Random effects** | **Posterior mean (SD)** | **Posterior mode** | **Lower CI** | **Upper CI** |  |
| Class | 0.10 (0.36) | 0.03 | 0.0002 | 0.33 |  |
| Family | 0.02 (0.02) | 0.001 | 0.0002 | 0.06 |  |
| Species | 0.01 (0.01) | 0.001 | 0.0002 | 0.04 |  |
| Study | 0.04 (0.02) | 0.03 | 0.01 | 0.07 |  |
| Residual variance | 0.002 (0.002) | 0.0007 | 0.0002 | 0.005 |  |

**S6(f) Model 5**

| **Fixed effects** | **Posterior mean (SD)** | **Posterior mode** | **Lower CI** | **Upper CI** | **pMCMC** |
| --- | --- | --- | --- | --- | --- |
| Competitor presence: no | 0.36 (0.15) | 0.32 | 0.11 | 0.66 | **0.01** |
| Competitor presence: yes | 0.29 (0.17) | 0.25 | -0.001 | 0.64 | **0.05** |
| yes - no | -0.07 (0.10) | -0.09 | -0.27 | 0.13 | 0.49 |
| **Random effects** | **Posterior mean (SD)** | **Posterior mode** | **Lower CI** | **Upper CI** |  |
| Class | 0.08 (0.18) | 0.006 | 0.0001 | 0.29 |  |
| Family | 0.02 (0.02) | 0.002 | 0.0001 | 0.06 |  |
| Species | 0.01 (0.01) | 0.001 | 0.0002 | 0.03 |  |
| Study | 0.04 (0.02) | 0.04 | 0.01 | 0.07 |  |
| Residual variance | 0.002 (0.002) | 0.001 | 0.0002 | 0.005 |  |

**S6(g) Model 6**

| **Fixed effects** | **Posterior mean (SD)** | **Posterior mode** | **Lower CI** | **Upper CI** | **pMCMC** |
| --- | --- | --- | --- | --- | --- |
| Genetic data: no | 0.40 (0.17) | 0.35 | 0.14 | 0.78 | **0.02** |
| Genetic data: yes | 0.33 (0.16) | 0.29 | 0.05 | 0.67 | **0.03** |
| yes - no | -0.07 (0.06) | -0.06 | -0.19 | 0.05 | 0.25 |
| **Random effects** | **Posterior mean (SD)** | **Posterior mode** | **Lower CI** | **Upper CI** |  |
| Class | 0.10 (0.31) | 0.02 | 0.0002 | 0.34 |  |
| Family | 0.02 (0.02) | 0.001 | 0.0002 | 0.06 |  |
| Species | 0.01 (0.01) | 0.001 | 0.0001 | 0.03 |  |
| Study | 0.04 (0.02) | 0.03 | 0.007 | 0.07 |  |
| Residual variance | 0.002 (0.002) | 0.0006 | 0.0002 | 0.005 |  |

**S4(h) Model 7**

| **Fixed effects** | **Posterior mean (SD)** | **Posterior mode** | **Lower CI** | **Upper CI** | **pMCMC** |
| --- | --- | --- | --- | --- | --- |
| Data: within male tests | 0.75 (0.19) | 0.71 | 0.41 | 1.14 | **0.001** |
| Data: across male tests | 0.34 (0.16) | 0.29 | 0.07 | 0.65 | **0.02** |
| Within - Across | 0.41 (0.11) | 0.41 | 0.20 | 0.64 | **<0.0001** |
| **Random effects** | **Posterior mean (SD)** | **Posterior mode** | **Lower CI** | **Upper CI** |  |
| Class | 0.08 (0.23) | 0.02 | 0.0002 | 0.30 |  |
| Family | 0.02 (0.02) | 0.002 | 0.0002 | 0.06 |  |
| Species | 0.01 (0.01) | 0.001 | 0.0002 | 0.04 |  |
| Study | 0.04 (0.02) | 0.04 | 0.01 | 0.08 |  |
| Residual variance | 0.002 (0.001) | 0.0006 | 0.0002 | 0.004 |  |

**S6(i) Model 8**

| **Fixed effects** | **Posterior mean (SD)** | **Posterior mode** | **Lower CI** | **Upper CI** | **pMCMC** |
| --- | --- | --- | --- | --- | --- |
| Care: amount | 0.31 (0.17) | 0.31 | 0.0001 | 0.67 | **0.04** |
| Care: probability | 0.45 (0.17) | 0.43 | 0.13 | 0.81 | **0.001** |
| probability -amount | 0.14 (0.08) | 0.15 | -0.02 | 0.29 | 0.08 |
| Data: observational | 0.32 (0.17) | 0.34 | 0.01 | 0.63 | **0.04** |
| Data: experimental | 0.32 (0.18) | 0.31 | 0.01 | 0.69 | **0.04** |
| experimental - observational | 0.03 (0.10) | 0.04 | -0.18 | 0.21 | 0.78 |
| Female access: no | 0.31 (0.17) | 0.28 | 0.004 | 0.65 | **0.04** |
| Female access: yes | 0.42 (0.17) | 0.37 | 0.10 | 0.75 | **0.01** |
| yes - no | 0.11 (0.11) | 0.10 | -0.11 | 0.32 | 0.29 |
| Competitor presence: no | 0.31 (0.17) | 0.33 | 0.02 | 0.68 | **0.04** |
| Competitor presence: yes | 0.22 (0.18) | 0.16 | -0.11 | 0.57 | 0.15 |
| yes - no | -0.09 (0.11) | -0.06 | -0.30 | 0.14 | 0.42 |
| Genetic data: no | 0.31 (0.17) | 0.25 | 0.005 | 0.66 | **0.04** |
| Genetic data: yes | 0.29 (0.14) | 0.25 | 0.03 | 0.58 | **0.02** |
| yes - no | -0.03 (0.10) | -0.04 | -0.23 | 0.16 | 0.76 |
| Within Male Tests | 0.74 (0.22) | 0.75 | 0.36 | 1.17 | **0.003** |
| Across Male Tests | 0.31 (0.19) | 0.30 | 0.003 | 0.68 | **0.05** |
| Within - Across | 0.43 (0.11) | 0.41 | 0.20 | 0.65 | **<0.0001** |
| **Random effects** | **Posterior mean (SD)** | **Posterior mode** | **Lower CI** | **Upper CI** |  |
| Class | 0.08 (0.18) | 0.007 | 0.0002 | 0.28 |  |
| Family | 0.02 (0.02) | 0.002 | 0.0002 | 0.06 |  |
| Species | 0.01 (0.01) | 0.001 | 0.0002 | 0.04 |  |
| Study | 0.04 (0.02) | 0.02 | 0.006 | 0.07 |  |
| Residual variance | 0.002 (0.001) | 0.0007 | 0.0002 | 0.005 |  |
