# Supplementary material for: Why Do Cuckolded Males Provide Paternal Care?
Source: PLoS Biol. 2013 Mar 26;11(3):e1001520. doi: 10.1371/journal.pbio.1001520 (PMC3608547; doi:10.1371/journal.pbio.1001520)
Supplement: Table S7 — Meta-analysis of adjustment: biological effects. (DOCX) [file pbio.1001520.s011.docx]

**Table S7. Meta-analysis of the adjustment of male care: biological effects**

**S7(a) Modeling summary**

| **Table** | **Model** | **Fixed effects** |  | **Variation explained by random effects (%)** | | | |
| --- | --- | --- | --- | --- | --- | --- | --- |
|  |  |  | **DIC** | **Class** | **Family** | **Species** | **Study** |
| S7(b) | 1 | ZrBenefit | -572.60 | 34.06 | 17.21 | 9.56 | 37.30 |
|  | 2 | ZrBenefit + amount vs probability of care measured for ZrBenefit + ZrBenefit : amount vs probability of care measured for ZrBenefit | -571.01 | 28.22 | 17.57 | 10.61 | 41.57 |
| S7(c) | 3 | Proportion of male care | -571.01 | 40.16 | 16.67 | 8.64 | 32.78 |
| S7(d) | 4 | ZrCost | -570.37 | 23.37 | 18.88 | 11.55 | 43.89 |
|  | 5 | ZrCost + measure of cost + ZrCost : measure of cost | -574.13 | 27.25 | 12.25 | 12.32 | 46.02 |
| S7(e) | 6 | Multiple paternity | -567.79 | 29.06 | 17.91 | 12.40 | 38.53 |
| S7(f) | 7 | Multiple paternity + Multiple paternity^2^ | -570.09 | 26.78 | 17.34 | 13.97 | 39.81 |
| S7(g) | 8 | Within vs Across males + Amount vs probability of care for ZrAdjust + ZrBenefit + ZrCost + multiple paternity | -585.45 | 15.90 | 18.32 | 14.90 | 48.79 |
|  | 9 | Full model + ZrCost : ZrBenefit | -588.47 | 15.33 | 18.75 | 15.04 | 48.75 |
| **S7(h)** | **10** | **Full model + ZrCost : multiple paternity** | **-594.77** | **17.82** | **20.55** | **14.23** | **45.24** |
|  | 11 | Full model + ZrBenefit :multiple paternity | -593.49 | 16.08 | 17.76 | 17.62 | 46.56 |
|  | 12 | Full model + ZrCost : ZrBenefit + ZrCost : Zr multiple paternity | -589.40 | 17.03 | 20.55 | 14.86 | 45.38 |
|  | 13 | Full model + ZrCost : ZrBenefit + ZrBenefit : Zr multiple paternity | -588.32 | 15.13 | 18.77 | 17.62 | 46.42 |
|  | 14 | Full model + ZrCost : multiple paternity + ZrBenefit : multiple paternity | -589.12 | 17.88 | 20.12 | 16.29 | 43.60 |
|  | 15 | Full model + ZrCost : ZrBenefit + ZrCost : multiple paternity +  ZrBenefit : multiple paternity | -588.85 | 17.07 | 20.36 | 16.12 | 44.38 |
|  | 17 | Full model + ZrCost : ZrBenefit + ZrCost : multiple paternity +  ZrBenefit : multiple paternity + ZrCost : multiple paternity : ZrBenefit | -593.69 | 17.04 | 20.59 | 16.27 | 44.04 |
|  | 18 | Full model + Within vs Across males: ZrCost + Within vs Across males : multiple paternity + ZrCost : multiple paternity + Within vs Across males : ZrCost : multiple paternity | -590.65 | 17.53 | 22.90 | 13.83 | 43.51 |
| N_datapoints_=192, N_studies_=62, N_species_=48, N_families_=29, N_classes_=5. Full model = model 8. Bold = best model. | | | |  | | | |

**S7(b) Model 1**

| **Fixed effects** | **Posterior mean (SD)** | **Posterior mode** | **Lower CI** | **Upper CI** | **pMCMC** |
| --- | --- | --- | --- | --- | --- |
| ZrBenefit | -0.01 (0.05) | 0.006 | -0.10 | 0.08 | 0.78 |
| **Random effects** | **Posterior mean (SD)** | **Posterior mode** | **Lower CI** | **Upper CI** |  |
| Class | 0.08 (0.33) | 0.03 | 0.0002 | 0.27 |  |
| Family | 0.02 (0.02) | 0.002 | 0.0002 | 0.06 |  |
| Species | 0.01 (0.01) | 0.001 | 0.0002 | 0.03 |  |
| Study | 0.04 (0.2) | 0.04 | 0.01 | 0.07 |  |
| Residual variance | 0.002 (0.002) | 0.001 | 0.0002 | 0.005 |  |

**S7(c) Model 3**

| **Fixed effects** | **Posterior mean (SD)** | **Posterior mode** | **Lower CI** | **Upper CI** | **pMCMC** |
| --- | --- | --- | --- | --- | --- |
| Proportion of care | -0.003 (0.08) | -0.02 | -0.15 | 0.16 | 0.95 |
| **Random effects** | **Posterior mean (SD)** | **Posterior mode** | **Lower CI** | **Upper CI** |  |
| Class | 0.10 (0.30) | 0.03 | 0.0002 | 0.39 |  |
| Family | 0.02 (0.02) | 0.001 | 0.0002 | 0.06 |  |
| Species | 0.01 (0.01) | 0.001 | 0.0002 | 0.03 |  |
| Study | 0.04 (0.02) | 0.03 | 0.01 | 0.07 |  |
| Residual variance | 0.002 (0.002) | 0.001 | 0.0002 | 0.005 |  |

**S7(d) Model 4**

| **Fixed effects** | **Posterior mean (SD)** | **Posterior mode** | **Lower CI** | **Upper CI** | **pMCMC** |
| --- | --- | --- | --- | --- | --- |
| ZrCost | 0.09 (0.06) | 0.09 | -0.02 | 0.21 | 0.11 |
| **Random effects** | **Posterior mean (SD)** | **Posterior mode** | **Lower CI** | **Upper CI** |  |
| Class | 0.04 (0.15) | 0.005 | 0.0002 | 0.17 |  |
| Family | 0.02 (0.02) | 0.001 | 0.0003 | 0.05 |  |
| Species | 0.01 (0.01) | 0.001 | 0.0002 | 0.03 |  |
| Study | 0.04 (0.02) | 0.03 | 0.01 | 0.08 |  |
| Residual variance | 0.002 (0.002) | 0.001 | 0.0002 | 0.005 |  |

**S7(e) Model 6**

| **Fixed effects** | **Posterior mean (SD)** | **Posterior mode** | **Lower CI** | **Upper CI** | **pMCMC** |
| --- | --- | --- | --- | --- | --- |
| Multiple paternity | 0.05 (0.04) | 0.03 | -0.04 | 0.14 | 0.28 |
| **Random effects** | **Posterior mean (SD)** | **Posterior mode** | **Lower CI** | **Upper CI** |  |
| Class | 0.06 (0.15) | 0.007 | 0.0002 | 0.22 |  |
| Family | 0.02 (0.02) | 0.002 | 0.0002 | 0.06 |  |
| Species | 0.01 (0.01) | 0.002 | 0.0002 | 0.04 |  |
| Study | 0.04 (0.02) | 0.03 | 0.01 | 0.07 |  |
| Residual variance | 0.002 (0.002) | 0.0008 | 0.0002 | 0.005 |  |

**S7(f) Model 7**

| **Fixed effects** | **Posterior mean (SD)** | **Posterior mode** | **Lower CI** | **Upper CI** | **pMCMC** |
| --- | --- | --- | --- | --- | --- |
| Multiple paternity | 0.05 (0.04) | 0.05 | -0.03 | 0.14 | 0.25 |
| Multiple paternity^2^ | 0.05 (0.05) | 0.04 | -0.04 | 0.13 | 0.27 |
| **Random effects** | **Posterior mean (SD)** | **Posterior mode** | **Lower CI** | **Upper CI** |  |
| Class | 0.05 (0.15) | 0.009 | 0.0002 | 0.22 |  |
| Family | 0.02 (0.02) | 0.001 | 0.0002 | 0.05 |  |
| Species | 0.01 (0.01) | 0.001 | 0.0002 | 0.04 |  |
| Study | 0.04 (0.02) | 0.03 | 0.01 | 0.07 |  |
| Residual variance | 0.002 (0.002) | 0.001 | 0.0002 | 0.005 |  |

**S7(g) Model 8**

| **Fixed effects** | **Posterior mean (SD)** | **Posterior mode** | **Lower CI** | **Upper CI** | **pMCMC** |
| --- | --- | --- | --- | --- | --- |
| Care: amount | 0.24 (0.11) | 0.19 | 0.06 | 0.46 | **0.01** |
| Care: probability | 0.39 (0.12) | 0.39 | 0.17 | 0.61 | **0.004** |
| probability -amount | 0.15 (0.08) | 0.18 | -0.001 | 0.32 | 0.06 |
| Within Male Tests | 0.65 (0.15) | 0.65 | 0.35 | 0.94 | **<0.0001** |
| Across Male Tests | 0.23 (0.10) | 0.21 | 0.05 | 0.43 | **0.01** |
| Within vs Across Males | 0.42 (0.11) | 0.41 | 0.20 | 0.63 | **0.0004** |
| ZrBenefit | -0.04 (0.05) | -0.04 | -0.15 | 0.04 | 0.34 |
| ZrCost | 0.10 (0.06) | 0.11 | -0.01 | 0.22 | 0.10 |
| Multiple paternity | 0.05 (0.04) | 0.05 | -0.04 | 0.13 | 0.29 |
| **Random effects** | **Posterior mean (SD)** | **Posterior mode** | **Lower CI** | **Upper CI** |  |
| Class | 0.02 (0.09) | 0.008 | 0.0001 | 0.09 |  |
| Family | 0.02 (0.02) | 0.001 | 0.0002 | 0.05 |  |
| Species | 0.01 (0.01) | 0.001 | 0.0002 | 0.04 |  |
| Study | 0.04 (0.02) | 0.04 | 0.01 | 0.08 |  |
| Residual variance | 0.002 (0.001) | 0.0007 | 0.0002 | 0.004 |  |

**S7(h) Model 10**

| **Fixed effects** | **Posterior mean (SD)** | **Posterior mode** | **Lower CI** | **Upper CI** | **pMCMC** |
| --- | --- | --- | --- | --- | --- |
| Care: amount | 0.23 (0.10) | 0.23 | 0.06 | 0.44 | **0.01** |
| Care: probability | 0.36 (0.11) | 0.36 | 0.15 | 0.59 | **0.004** |
| probability -amount | 0.13 (0.08) | 0.15 | -0.03 | 0.28 | 0.10 |
| Within Male Tests | 0.66 (0.15) | 0.63 | 0.37 | 0.96 | **0.0004** |
| Across Male Tests | 0.23 (0.10) | 0.21 | 0.05 | 0.44 | **0.01** |
| Within vs Across Males | 0.42 (0.11) | 0.44 | 0.21 | 0.63 | **<0.0001** |
| ZrBenefit | -0.03 (0.05) | -0.02 | -0.12 | 0.07 | 0.57 |
| ZrCost | 0.02 (0.07) | 0.02 | -0.11 | 0.15 | 0.77 |
| Multiple paternity | 0.02 (0.04) | 0.03 | -0.07 | 0.10 | 0.62 |
| ZrCost : Multiple paternity | 0.11 (0.05) | 0.13 | 0.01 | 0.22 | **0.02** |
| **Random effects** | **Posterior mean (SD)** | **Posterior mode** | **Lower CI** | **Upper CI** |  |
| Class | 0.02 (0.07) | 0.003 | 0.0002 | 0.10 |  |
| Family | 0.02 (0.02) | 0.001 | 0.0003 | 0.05 |  |
| Species | 0.01 (0.01) | 0.002 | 0.0003 | 0.04 |  |
| Study | 0.04 (0.02) | 0.04 | 0.008 | 0.07 |  |
| Residual variance | 0.002 (0.001) | 0.0009 | 0.0002 | 0.004 |  |

**S7(i) Model 18**

| **Fixed effects** | **Posterior mean (SD)** | **Posterior mode** | **Lower CI** | **Upper CI** | **pMCMC** |
| --- | --- | --- | --- | --- | --- |
| Care: amount | 0.23 (0.10) | 0.23 | 0.06 | 0.44 | **0.01** |
| Care: probability | 0.36 (0.11) | 0.36 | 0.15 | 0.59 | **0.004** |
| probability -amount | 0.13 (0.08) | 0.15 | -0.03 | 0.28 | 0.10 |
| Within Male Tests | 0.66 (0.15) | 0.63 | 0.37 | 0.96 | **0.0004** |
| Across Male Tests | 0.23 (0.10) | 0.21 | 0.05 | 0.44 | **0.01** |
| Within vs Across Males | 0.42 (0.11) | 0.44 | 0.21 | 0.63 | **<0.0001** |
| ZrBenefit | -0.03 (0.05) | -0.02 | -0.12 | 0.07 | 0.57 |
| ZrCost | 0.02 (0.07) | 0.02 | -0.11 | 0.15 | 0.77 |
| Multiple paternity | 0.02 (0.04) | 0.03 | -0.07 | 0.10 | 0.62 |
| ZrCost : Multiple paternity | 0.11 (0.05) | 0.13 | 0.01 | 0.22 | **0.02** |
| ZrCost : Within vs Across | 0.30 (0.22) | 0.31 | -0.10 | 0.79 | 0.17 |
| Multiple paternity : Within vs Across | 0.39 (0.19) | 0.38 | -0.02 | 0.78 | 0.06 |
| ZrCost : Multiple paternity : Within vs Across | 5.58 (3.54) | 5.87 | -1.82 | 11.97 | 0.11 |
| **Random effects** | **Posterior mean (SD)** | **Posterior mode** | **Lower CI** | **Upper CI** |  |
| Class | 0.02 (0.09) | 0.007 | 0.0002 | 0.09 |  |
| Family | 0.02 (0.02) | 0.001 | 0.0002 | 0.05 |  |
| Species | 0.01 (0.01) | 0.002 | 0.0002 | 0.04 |  |
| Study | 0.03 (0.02) | 0.03 | 0.008 | 0.07 |  |
| Residual variance | 0.002 (0.001) | 0.0008 | 0.0008 | 0.004 |  |
