# Supplementary material for: Why Do Cuckolded Males Provide Paternal Care?
Source: PLoS Biol. 2013 Mar 26;11(3):e1001520. doi: 10.1371/journal.pbio.1001520 (PMC3608547; doi:10.1371/journal.pbio.1001520)
Supplement: Table S8 — Phylogenetic meta-analysis of adjustment of paternal care across just birds. (DOCX) [file pbio.1001520.s012.docx]

**Table S8. Meta-analysis of the adjustment of male care: phylogenetic analysis across birds**

| **Fixed effects** | **Posterior mean (SD)** | **Posterior mode** | **Lower CI** | **Upper CI** | **pMCMC** |
| --- | --- | --- | --- | --- | --- |
| Care: amount | 0.18 (0.12) | 0.20 | -0.07 | 0.41 | 0.10 |
| Care: probability | 0.32 (0.15) | 0.31 | 0.03 | 0.63 | **0.03** |
| probability -amount | 0.14 (0.11) | 0.13 | -0.06 | 0.36 | 0.19 |
| Within Male Tests | 0.62 (0.15) | 0.60 | 0.34 | 0.92 | **0.0004** |
| Across Male Tests | 0.17 (0.10) | 0.17 | -0.03 | 0.40 | 0.08 |
| Within vs Across Males | 0.44 (0.11) | 0.47 | 0.23 | 0.66 | **<0.0001** |
| ZrBenefit | 0.007 (0.15) | -0.02 | -0.30 | 0.31 | 0.96 |
| ZrCost | -0.07 (0.10) | -0.05 | -0.27 | 0.12 | 0.45 |
| Multiple paternity | 0.05 (0.06) | 0.02 | -0.08 | 0.18 | 0.44 |
| ZrCost : Multiple paternity | 0.17 (0.08) | 0.15 | 0.007 | 0.33 | **0.04** |
| **Random effects** | **Posterior mean (SD)** | **Posterior mode** | **Lower CI** | **Upper CI** | **% variation** |
| Phylogeny | 0.03 (0.04) | 0.001 | 0.0002 | 0.11 | 27.20 |
| Study | 0.06 (0.03) | 0.05 | 0.02 | 0.12 | 70.72 |
| Residual variance | 0.002 (0.001) | 0.001 | 0.0002 | 0.005 |  |
