# Supplementary material for: Why Do Cuckolded Males Provide Paternal Care?
Source: PLoS Biol. 2013 Mar 26;11(3):e1001520. doi: 10.1371/journal.pbio.1001520 (PMC3608547; doi:10.1371/journal.pbio.1001520)
Supplement: Text S1 — Supplementary results. (DOCX) [file pbio.1001520.s013.docx]

**Supplementary Results**

**1. The effect of cuckoldry on rAdjust**

We assume that variance in paternity increases with higher rates of multiple paternity in our analyses. When multiple paternity is 0% and 100% there is no variance in the risk of cuckoldry and so no potential benefit to gained from adjusting paternal care. At 0% the male is related to all offspring and at 100% there is no opportunity for males to gain higher paternity in alternative breeding attempts. However, when multiple paternity is 50% variance in the risk of cuckoldry is maximized and selection for adjustment is expected to be strongest. Our measure of multiple paternity (% of breeding attempts where a male was cuckolded) ranged from 6 to 92%, but not all offspring in these attempts were sired by other males. The number of offspring fathered by a male other than the caring male ranged from 5 to 71% (excluding superb fairy wren, range = 5 to 55%). However, to take into account the possibility that selection for adjustment would be reduced in species where the multiple paternity was greater than 50%, we tested for a quadratic effect of multiple paternity on rAdjust and found no relationship (Table S7f).

**2. The effect of different methodological approaches on estimates of rCost and rBenefit and how this influences relationships with rAdjust**

When quantifying rCost we found that studies that measured the influence of care on future reproductive success and mating opportunities had larger effect sizes than studies measuring survival (Table S5e). Survival can be very difficult to measure as it requires tracking individuals over long periods of time during which time mortality can be influenced by a variety of factors and individuals can disperse. In contrast, in our dataset the influence of male care on future reproductive success and mating opportunities was typically examined during the same or the following breeding season, which may minimize the influence of other factors that mask the costs of care when survival is measured. When calculating rBenefit we found that studies that measured the probability of care detected larger effects than studies that measured the amount of care males provided (Table S4c).

Given the differences in the effect sizes of rBenefit and rCost according to how they were measured we tested whether this influenced the relationships between rAdjust and rBenefit and rCost (Table S7). We found that models including the way rCost and rBenefit were measured did not explain more variation in rAdjust (did not have lower DIC values) than models just including averaged estimates of rCost and rBenefit (Table S7).

**3. The effect of phylogenetic history on rAdjust, rCost and rBenefit**

When estimating the mean effect size of rAdjust using models that only included taxonomic relationships between species it was clear that common ancestry between species, particular at the level of taxonomic class, was important in explaining variation in rAdjust (c.35% of residual variation explained by class: Table 6a). However, after accounting for variation in the risk of cuckoldry and the costs of care for males, the variation in rAdjust explained by taxanomic class was greatly reduced (16% of residual variation: Table S7). Furthermore, after accounting for all other effects (see Table 7h) differences between families and between species explained c.20% and c.10% of residual variation in rAdjust, respectively. This is consistent with the phylogenetic analysis across birds which indicated that 26% of variation in rAdjust was explained by phylogenetic history. Similarly, a large proportion of variation in rBenefit and rCost was explained by differences between taxonomic classes (Table S4: rBenefit 67%. Table S5: rCost = 50%), indicating that major transitions to different life forms (e.g aquatic versus terrestrial versus aerial) have had a major impact on the costs and benefits of paternal care.

Together these results indicate that a substantial amount of variation in the degree to which males adjust care in response to paternity and the costs and benefits of care are determined by factors that are reasonably well conserved across evolutionary time. It is likely that there are many life-history characteristics that we have not examined here that are shared among closely related species that influence the fitness payoffs of adjustment and providing paternal care. However, it is also worth noting that around 40% of unexplained variation in rAdjust was attributable to differences between studies indicating that the context in which adjustment is measured may have a pronounced effect on the extent to which cuckolded males reduce care.
